# Supplementary material for: Exploring failure of antimicrobial prophylaxis and pre-emptive therapy for transplant recipients: a systematic review
Source: BMJ Open. 2020 Jan 7;10(1):e034940. doi: 10.1136/bmjopen-2019-034940 (PMC6955515; doi:10.1136/bmjopen-2019-034940)
Supplement: Supplementary data [file bmjopen-2019-034940supp001.pdf]

Appendix 1. Study protocol (PROSPERO CRD42017077606)

PROSPERO International prospective register of systematic reviews

Review title and timescale

1 Review title

Exploring failure of antimicrobial prophylaxis and pre-emptive therapy for transplant recipients: a systematic review

2 Original language title

Not applicable

3 Anticipated or actual start date

01 October 2017

4 Anticipated completion date

09 February 2019

Review team details

5 Named contact

Anne-Grete Märtson

6 Named contact email

a.martson@umcg.nl

7 Named contact address

Dept. of Clinical Pharmacy and Pharmacology; EB 70 UMCG Hanzeplein 1, PO BOX 30001, 9700 RB Groningen the Netherlands

8 Named contact phone number

+37253325121

9 Organisational affiliation of the review

University Medical Center Groningen

Website address: umcg.nl

10 Review team members and their organisational affiliations

	Anne-Grete	Märtson	UMCG, the Netherlands
Dr	Martijn	Bakker	UMCG, the Netherlands
Prof.Dr.	Tjip.S.	van der Werf	UMCG, the Netherlands

Dr	Jan-Willem	Alffenaar	UMCG, the Netherlands

11 Funding sources/sponsors

This systematic review will be part of a PhD thesis that is funded by University Medical Centre Groningen and the European Union's Horizon 2020 research and innovation programme Under the Marie Skłodowska-Curie grant agreement 713660.

12 Conflicts of interest

There are no actual or potential conflicts of interest.

13 Collaborators

Title	First name	Last name	Organisation details
Dr	Hans	Blokzijl	UMCG, the Netherlands
Dr	Erik A.M.	Verschuuren	UMCG, the Netherlands
Dr	L.F.R.	Span	UMCG, the Netherlands
Dr	Stefan P.	Berger	UMCG, the Netherlands

Review methods

14 Review question(s)

It is widely known that solid organ and allogenic stem cell transplant patients are especially susceptible to opportunistic infections like Cytomegalovirus (CMV), *Pneumocystis jirovecii* pneumonia (PCP) and invasive fungal infections. In recent years, organ transplantation has developed greatly. Transplantation has become available for a bigger patient population and this has led to a wider range of infections after transplantation. The concern with appropriate prophylactic regimens remain as antifungal prophylaxis varies exceptionally among different transplant centres.

To prevent these infections from occurring different prophylactic strategies are used before and after transplantation like ganciclovir and valganciclovir for CMV and trimethoprim-sulfamethoxazole (TMP+SMX) for PCP. This has proven to be a beneficial approach, despite that there is still a proportion of patients whose prophylactic treatment fails as they develop breakthrough infections, suffer from adverse events or

simply do not tolerate medications.

This systematic review is aiming to summarise the main reasons why prophylaxis is failed for non-HIV immunocompromised patients looking at prospective studies done on this topic.

The objectives of this study are to review and analyse literature to determine which factors might contribute to failure of prophylaxis of opportunistic infections in transplant recipients.

The potential factors for failure are drug-drug interactions, microbiological susceptibility, drug pharmacokinetics, the dose of the medication, route of administration, transplant (specific organ or allogenic stem cell), status of the immunocompromised patient and medication compliance.

15 Searches

This systematic review will include prospective randomized controlled trials and prospective single-arm studies. There are no restrictions for the setting or language. Studies published from 1.01.2010 to present will be included to the systematic review.

To identify studies for this systematic review these databases will be searched: MEDLINE, EMBASE, Cochrane Central Register of Controlled Trials (CENTRAL) and PubMed.

16 URL to search strategy

PubMed search strategy

("Immunity, Cellular"[Mesh] OR "Immunocompromised Host"[Mesh] OR "Transplants"[Mesh] OR "Transplantation"[Mesh] OR immunocompromised [tiab] OR transplant*[tiab])

AND

("valganciclovir" [Supplementary Concept] OR "Ganciclovir"[Mesh] OR "Trimethoprim, Sulfamethoxazole Drug Combination"[Mesh] OR "Ciprofloxacin"[Mesh:NoExp] OR "posaconazole" [Supplementary Concept] OR "Voriconazole"[Mesh] OR "Fluconazole"[Mesh] OR valganciclovir [tiab] OR Ganciclovir [tiab] OR Trimethoprim [tiab] OR Sulfamethoxazole [tiab] OR Ciprofloxacin [tiab] OR posaconazole [tiab] OR Voriconazole [tiab] OR Fluconazole [tiab] OR cotrimoxazole [tiab)

NOT (HIV OR Human immunodeficiency virus)

AND

("Randomized Controlled Trial" [Publication Type] OR randomi*[tiab] OR randomly[tiab] OR trial[ti] OR prospective [tiab] OR single arm [tiab])

17 Condition or domain being studied

Prophylaxis for opportunistic infections in transplant recipients.

18 Participants/population

The study will include adult patients (16 years and older) who have received:

- allogeneic stem cell transplant
- lung transplant
- kidney transplant
- liver transplant
- heart transplant
- pancreas transplant
- small bowel transplant

19 Intervention(s), exposure(s)

All patients who have received prophylaxis or preemptive therapy for:

- PCP with TMP+SMX
- febrile neutropenia with ciprofloxacin
- cytomegalovirus and human herpesvirus type 6 with ganciclovir and/or valganciclovir
- invasive fungal infections with posaconazole and/or voriconazole and/or fluconazole and/or itraconazole

20 Comparator(s)/control

Placebo or active comparator.

21 Types of study to be included

This systematic review will include prospective randomized controlled trials and prospective single-arm studies.

22 Context

There are no restrictions for the setting.

23 Primary outcome(s)

Primary outcomes:

- adverse effects leading to stopping of treatment or switching medication or dose reduction

Märtson A-G, *et al. BMJ Open* 2020; 10:e034940. doi: 10.1136/bmjopen-2019-034940

- breakthrough infection

24 **Secondary outcomes**

Secondary outcomes:

- resistance to antimicrobials/antifungals/antivirals
- death

25 **Data extraction (selection and coding)**

The literature search and data extraction for inclusion and eligibility for this systematic review will be done according to the inclusion criteria by two authors independently. If there are discrepancies between the results these will be discussed and resolved with a third author.

If there is data missing or additional questions from the selected studies then the authors of these studies will be contacted directly.

A form will be developed to document the characteristics and quality of the included studies.

26 **Risk of bias (quality) assessment**

Risk of bias in individual studies will be assessed independently by the two researchers who did the study selection and data extraction. For assessing bias in individual studies Revised Cochrane risk-of-bias tool for randomized trials (RoB 2) will be used for RCTs and Risk of Bias in Non-randomized Studies of Interventions (ROBINS-I) will be used for prospective single-arm studies

If there are discrepancies between the authors these will be discussed and resolved with a third author.

27 **Strategy for data synthesis**

The criteria how the data will be quantitatively synthesised and if and how subgroup analysis will be done can be determined after the data is available.

The possibility of performing a meta-analysis and summary measures can also be determined after literature search and when we have done the data extraction.
After the preliminary searches we expect that there will not be sufficient data to conduct a meta-analysis.

28 **Analysis of subgroups or subsets**

The possibility and method for subgroup analysis can be determined after the data is available and

Märtson A-G, *et al. BMJ Open* 2020; 10:e034940. doi: 10.1136/bmjopen-2019-034940

extracted.

If subgroup analysis is applicable then the subgroups could be different transplant types.

Review general information

29 Type and method of review

Systematic review

30 Language

English

31 Country

The Netherlands

32 Other registration details

Not applicable

33 Reference and/or URL for published protocol

Not applicable

34 Dissemination plans

This systematic review will be submitted to a journal specific to this field.

35 Keywords

systematic review; immunocompromised patients; prophylaxis; antimicrobials; antifungals; antivirals; PCP; CMV; febrile neutropenia; invasive fungal infection; opportunistic infection; transplantation

36 Details of any existing review of the same topic by the same authors

There has not been a previous systematic review done on this topic before.

37 Current review status

Conpleted (not published)

38 Any additional information

This review is part of planning further retrospective or prospective studies on prophylactic regimens for immunocompromised patients.

39 Details of final report/publication(s)

Not applicable

Appendix 2. PRISMA 2009 Checklist

Section/topic	#	Checklist item	Reported on page #
**TITLE**			
Title	1	Identify the report as a systematic review, meta-analysis, or both.	1
**ABSTRACT**			
Structured summary	2	Provide a structured summary including, as applicable: background; objectives; data sources; study eligibility criteria, participants, and interventions; study appraisal and synthesis methods; results; limitations; conclusions and implications of key findings; systematic review registration number.	2
**INTRODUCTION**			
Rationale	3	Describe the rationale for the review in the context of what is already known.	3
Objectives	4	Provide an explicit statement of questions being addressed with reference to participants, interventions, comparisons, outcomes, and study design (PICOS).	3
**METHODS**			
Protocol and registration	5	Indicate if a review protocol exists, if and where it can be accessed (e.g., Web address), and, if available, provide registration information including registration number.	3
Eligibility criteria	6	Specify study characteristics (e.g., PICOS, length of follow-up) and report characteristics (e.g., years considered, language, publication status) used as criteria for eligibility, giving rationale.	4
Information sources	7	Describe all information sources (e.g., databases with dates of coverage, contact with study authors to identify additional studies) in the search and date last searched.	4
Search	8	Present full electronic search strategy for at least one database, including any limits used, such that it could be repeated.	4, 46-47
Study selection	9	State the process for selecting studies (i.e., screening, eligibility, included in systematic review, and, if applicable, included in the meta-analysis).	4
Data collection process	10	Describe method of data extraction from reports (e.g., piloted forms, independently, in duplicate) and any processes for obtaining and confirming data from investigators.	4-5
Data items	11	List and define all variables for which data were sought (e.g., PICOS, funding sources) and any assumptions and simplifications made.	4

Risk of bias in individual studies	12	Describe methods used for assessing risk of bias of individual studies (including specification of whether this was done at the study or outcome level), and how this information is to be used in any data synthesis.	5
Summary measures	13	State the principal summary measures (e.g., risk ratio, difference in means).	5
Synthesis of results	14	Describe the methods of handling data and combining results of studies, if done, including measures of consistency (e.g., I^2) for each meta-analysis.	NA

Section/topic	#	Checklist item	Reported on page #
Risk of bias across studies	15	Specify any assessment of risk of bias that may affect the cumulative evidence (e.g., publication bias, selective reporting within studies).	5
Additional analyses	16	Describe methods of additional analyses (e.g., sensitivity or subgroup analyses, meta-regression), if done, indicating which were pre-specified.	5
**RESULTS**			
Study selection	17	Give numbers of studies screened, assessed for eligibility, and included in the review, with reasons for exclusions at each stage, ideally with a flow diagram.	23
Study characteristics	18	For each study, present characteristics for which data were extracted (e.g., study size, PICOS, follow-up period) and provide the citations.	27-31
Risk of bias within studies	19	Present data on risk of bias of each study and, if available, any outcome level assessment (see item 12).	7, 35-36
Results of individual studies	20	For all outcomes considered (benefits or harms), present, for each study: (a) simple summary data for each intervention group (b) effect estimates and confidence intervals, ideally with a forest plot.	33-36
Synthesis of results	21	Present results of each meta-analysis done, including confidence intervals and measures of consistency.	NA
Risk of bias across studies	22	Present results of any assessment of risk of bias across studies (see Item 15).	35-36
Additional analysis	23	Give results of additional analyses, if done (e.g., sensitivity or subgroup analyses, meta-regression [see Item 16]).	5-7
**DISCUSSION**			
Summary of evidence	24	Summarize the main findings including the strength of evidence for each main outcome; consider their relevance to key groups (e.g., healthcare providers, users, and policy makers).	7-10
Limitations	25	Discuss limitations at study and outcome level (e.g., risk of bias), and at review-level (e.g., incomplete retrieval of identified research, reporting bias).	9-10
Conclusions	26	Provide a general interpretation of the results in the context of other evidence, and implications for future research.	10

**FUNDING**			
Funding	27	Describe sources of funding for the systematic review and other support (e.g., supply of data); role of funders for the systematic review.	10

From: Moher D, Liberati A, Tetzlaff J, Altman DG, The PRISMA Group (2009). Preferred Reporting Items for Systematic Reviews and Meta-Analyses: The PRISMA Statement. PLoS Med 6(7): e1000097. doi:10.1371/journal.pmed1000097

For more information, visit: **www.prisma-statement.org**.

Appendix 3. Search strategy of EMBASE and CENTRAL

MEDLINE (PubMed) search strategy

("Immunity, Cellular"[Mesh] OR "Immunocompromised Host"[Mesh] OR "Transplants"[Mesh] OR "Transplantation"[Mesh] OR "Transplant Recipients"[Mesh] OR immunocompromised [tiab] OR kidney transplant* [tiab] OR heart transplant* [tiab] OR pancreas transplant* [tiab] OR lung transplant* [tiab] OR stem cell transplant* [tiab] OR small bowel transplant* [tiab] OR liver transplant* [tiab] OR small-bowel transplant* [tiab] OR allogeneic stem cell transplant* [tiab] OR transplant*[tiab])

AND

("valganciclovir" [Supplementary Concept] OR "Antibiotic Prophylaxis"[Mesh] OR "Ganciclovir"[Mesh] OR "Trimethoprim, Sulfamethoxazole Drug Combination"[Mesh] OR "Ciprofloxacin"[Mesh:NoExp] OR "posaconazole" [Supplementary Concept] OR "Voriconazole"[Mesh] OR "Fluconazole"[Mesh] OR "Anti-Infective Agents"[Mesh] OR valganciclovir [tiab] OR Ganciclovir [tiab] OR Trimethoprim [tiab] OR Sulfamethoxazole [tiab] OR Ciprofloxacin [tiab] OR posaconazole [tiab] OR Voriconazole [tiab] OR Fluconazole [tiab] OR bactrim [tiab] OR biseptol [tiab] OR ciprinol [tiab] OR antimicrobial [tiab] OR antibacterial [tiab] OR anti-microbial [tiab] anti-bacterial [tiab] OR anti-fungal [tiab] OR antifungal [tiab] OR antiviral [tiab] OR anti-viral [tiab] OR itraconazole [tiab] OR cotrimoxazole [tiab) NOT (HIV OR Human immunodeficiency virus)

AND

("Randomized Controlled Trial" [Publication Type] OR "Randomized Controlled Trials as Topic"[Mesh] OR randomi*[tiab] OR randomly[tiab] OR trial[tiab] OR prospective [tiab] OR single arm [tiab).

EMBASE search strategy

('immunocompromised patient'/exp OR 'transplantation'/exp OR transplant*:ab,ti OR 'graft recipient'/exp OR immunocompromised:ab,ti OR ‘kidney transplant*’:ab,ti OR ‘heart

transplant*’:ab,ti OR ‘pancreas transplant*’:ab,ti OR ‘lung transplant*’:ab,ti OR ‘small bowel transplant*’:ab,ti OR ‘liver transplant*’:ab,ti OR ‘small-bowel transplant*’:ab,ti OR ‘allogeneic stem cell transplant*’:ab,ti OR ‘stem cell transplant*’:ab,ti)

AND

('valganciclovir'/exp OR 'ganciclovir'/exp OR 'cotrimoxazole'/exp OR 'ciprofloxacin'/exp OR 'posaconazole'/exp OR 'voriconazole'/exp OR 'fluconazole'/exp OR 'itraconazole'/exp OR valganciclovir:ab,ti OR ganciclovir:ab,ti OR trimethoprim:ab,ti OR sulfamethoxazole:ab,ti OR ciprofloxacin:ab,ti OR posaconazole:ab,ti OR voriconazole:ab,ti OR itraconazole:ab,ti OR fluconazole:ab,ti OR bactrim:ab,ti OR biseptol:ab,ti OR ciprinol:ab,ti) NOT (HIV OR Human immunodeficiency virus) AND

('randomized controlled trial'/exp OR 'prospective study'/exp OR randomi*:ab,ti OR randomly:ab,ti OR trial:ab,ti OR prospective:ab,ti OR ‘single arm’:ab,ti)

CENTRAL search strategy

(Immunocompromised Host OR Transplants OR Transplantation OR Transplant Recipients OR immunocompromised OR transplant* OR kidney transplant* OR heart transplant* OR pancreas transplant* OR lung transplant* OR stem cell transplant* OR small bowel transplant* OR liver transplant* OR small-bowel transplant* OR allogeneic stem cell transplant*)

AND

(valganciclovir OR Antibiotic Prophylaxis OR Ganciclovir OR Trimethoprim, Sulfamethoxazole Drug Combination OR Ciprofloxacin OR posaconazole OR Voriconazole OR Fluconazole OR Anti-Infective Agents OR Trimethoprim OR Sulfamethoxazole OR bactrim OR biseptol OR ciprinol OR itraconazole OR antimicrobial OR antibacterial OR anti-microbial or anti-bacterial OR anti-fungal OR antifungal OR antiviral OR anti-viral OR cotrimoxazole) NOT (HIV OR Human immunodeficiency virus)

AND (Randomized Controlled Trial OR randomi* OR randomly OR trial OR prospective OR single arm)

Appendix 4. Data extraction form

- Reference (First author / Year / Title / Journal / Country)

- Study design
  - RCT
  - Single-arm study

- Sample size
  - Number of subjects started study
  - Number of subjects finished study

- Duration of study
  - Prophylaxis/preemptive therapy duration
  - Follow-up duration

- Study population
  - Age range
  - Transplanted organ
  - Underlying diseases

- Intervention
  - Medication used for prohylaxis
  - Comparison if applicable

- Main outcome measures
  - Primary outcomes
  - Secondary outcomes

- Failure of prophylaxis

- Risk of bias
  - RoB or ROBINS risk of bias score – low/high/some concerns

- Strengths and limitations of study

- Key conclusions of authors

Appendix 5. Background of detection of failure in the included studies

Table 1. RCT study reporting

							Reference	RCT								
	**Humar 2010[1,2]**	**Halim 2016, Gheith 2017**	**Witzke 2012, 2018**	**Reischig 2012**	**Reichig 2011, 2015**	**Togashi 2011**	**Padulles 2016**	**Palmer 2010 Finlen Copeland 2011**	**Chawla 2011**	**Boeckh 2015**	**Kim 2010**	**Parrella 2012**	**Wintston 2014**	**Chaftari 2012**	**Huang 2012**	**Park 2016**
**Description of high risk patients**	*	*	*	*	*		*	*	*	*	*	*	*	*		*
**Patient adherence reported**	*	*	*	*	*	*	*	*		*					*	*
**AE/toxicity described/ reported**	*	*	*	*	*	*	*	*	*	*	*		*	*#	*	
**Definition of breakthrough infection**	*	*	*	*	*	*	*	*	PT	PT	PT	*	*	*	*	*
**Breakthrough infection reported**	*	*	*	*	*	*	*	*	PT	PT	PT		*	*	*	*
**Insufficient reporting of topics above mentioned in limitations**						*										

PT – pre-emptive therapy, not applicable

# - toxicity explicitly defined, measurement described

Märtson A-G, *et al. BMJ Open* 2020; 10:e034940. doi: 10.1136/bmjopen-2019-034940

Table 2. Single-arm study reporting

Topic	Montejo 2010	Nanmoku 2018	Perrottet 2009 Manuel 2010	Reference McGillicuddy 2010	Single-arm Takenaka 2012	Park 2012	Mitsani 2012	Cordonnier 2010
**Description of high risk patients**	*	*	*	*	*	*		
**Patient adherence reported**								*
**AE/toxicity described/ reported**	*		*#	*	*	*		*
**Definition of breakthrough infection**	*	*	*	PT	PT	PT	*	*
**Breakthrough infection reported**	*			PT	PT	PT	*	*
**Insufficient reporting of failure mentioned in limitations**								

PT – pre-emptive therapy, not applicable

# - toxicity explicitly defined, measurement described

Märtson A-G, *et al. BMJ Open* 2020; 10:e034940. doi: 10.1136/bmjopen-2019-034940
