# Supplementary material for: Exploring failure of antimicrobial prophylaxis and pre-emptive therapy for transplant recipients: a systematic review
Source: BMJ Open. 2020 Jan 7;10(1):e034940. doi: 10.1136/bmjopen-2019-034940 (PMC6955515; doi:10.1136/bmjopen-2019-034940)
Supplement: Author's manuscript [file bmjopen-2019-034940.draft_revisions.pdf]

BMJ Open is committed to open peer review. As part of this commitment we make the peer review history of every article we publish publicly available.

When an article is published we post the peer reviewers' comments and the authors' responses online. We also post the versions of the paper that were used during peer review. These are the versions that the peer review comments apply to.

The versions of the paper that follow are the versions that were submitted during the peer review process. They are not the versions of record or the final published versions. They should not be cited or distributed as the published version of this manuscript.

BMJ Open is an open access journal and the full, final, typeset and author-corrected version of record of the manuscript is available on our site with no access controls, subscription charges or pay-per-view fees (http://bmjopen.bmj.com).

If you have any questions on BMJ Open's open peer review process please email info.bmjopen@bmj.com

BMJ Open

Exploring failure of antimicrobial prophylaxis and pre-emptive therapy for transplant recipients: a systematic review

Journal:	*BMJ Open*
Manuscript ID	bmjopen-2019-034940
Article Type:	Original research
Date Submitted by the Author:	14-Oct-2019
Complete List of Authors:	Märtson, Anne-Grete; UMCG, Clinical Pharmacy and Pharmacology<br>Bakker, Martijn; UMCG, Hematology<br>Blokzijl, Hans; Universitair Medisch Centrum Groningen, Gastroenterology and Hepatology<br>Verschuuren, Erik; Universitair Medisch Centrum Groningen, Pulmonary Disease and Tuberculosis<br>Berger, Stefan; Universitair Medisch Centrum Groningen, Nephrology<br>Span, Lambert; UMCG, Hematology<br>van der Werf, Tjip S.; University Medical Center Groningen, Department of Pulmonary Diseases & Tuberculosis<br>Alffenaar, Jan-Willem; Rijksuniversiteit Groningen,
Keywords:	Transplant medicine < INTERNAL MEDICINE, INFECTIOUS DISEASES, Infection control < INFECTIOUS DISEASES

SCHOLARONE™
Manuscripts

Exploring failure of antimicrobial prophylaxis and pre-emptive therapy for transplant recipients: a systematic review

Running title: Failure of antimicrobial prophylaxis

Anne-Grete Märtson[1], Martijn Bakker[2], Hans Blokzijl[3], Erik A. M. Verschuuren[4], Stefan P. Berger[5], Lambert F. R. Span[2], Tjip S. van der Werf[4,5], Jan-Willem C. Alffenaar[1,6]*

[1.] University of Groningen, University Medical Center Groningen, Department of Clinical Pharmacy and Pharmacology, Groningen, The Netherlands.

[2.] University of Groningen, University Medical Center Groningen, Department of Hematology, Groningen, The Netherlands.

[3.] University of Groningen, University Medical Center Groningen, Department of Gastroenterology and Hepatology, Groningen, The Netherlands.

[4.] University of Groningen, University Medical Center Groningen, Department of Pulmonary Diseases and Tuberculosis, Groningen, The Netherlands.

[5.] University of Groningen, University Medical Center Groningen, Department of Internal Medicine, Groningen, The Netherlands.

[6.] University of Sydney, Faculty of Medicine and Health, Sydney Pharmacy School, and Westmead Hospital, Sydney Australia

Keywords: antimicrobial prophylaxis, pre-emptive therapy, transplant recipients, opportunistic infections, failure

*Address of correspondence:

Jan-Willem C. Alffenaar
University of Groningen, University Medical Center Groningen
Department of Clinical Pharmacy and Pharmacology
Po box 30.001
9700 RB Groningen
The Netherlands
Email: j.w.c.alffenaar@umcg.nl
Tel: +31 503614070
Fax: +31 503614087

Word count: 2763

For peer review only

Abstract

Objectives

Infections remain a threat for solid organ and stem cell transplant recipients. Antimicrobial prophylaxis and pre-emptive therapy have improved survival of these patients however the failure rates of prophylaxis are not negligible. The aim of this systematic review is to explore the reasons behind failure of antimicrobial prophylaxis and pre-emptive therapy.

Setting

This systematic review included prospective randomized controlled trials (RCT) and prospective single-arm studies.

Participants

The studies included were on prophylaxis and pre-emptive therapy of opportunistic infections in transplant recipients. Studies were included from databases MEDLINE, CENTRAL and Embase published until Oct. 1, 2018.

Primary and secondary outcome measures

Primary outcome measures were breakthrough infections, adverse events leading to stopping of treatment, switching medication or dose reduction. Secondary outcome measures were acquired resistance to antimicrobials, antifungals or antivirals and death.

Results

From 3317 identified records, 30 records from 24 studies with 2851 patients were included in the systematic review. Seventeen focused on prophylactic and pre-emptive treatment of cytomegalovirus (CMV) and seven studies on invasive fungal infection (IFI). The main reasons for failure of prophylaxis and pre-emptive therapy were adverse events and breakthrough infections, which were described in 50% and 38% of the included studies respectively. In 25% of the studies a detailed description of patients that experienced failure of prophylaxis or pre-emptive therapy was unclear or lacking.

Conclusions

For peer review only

Our results show that although failure is reported in the studies the level of detail prohibits a detailed analysis of failure of prophylaxis and pre-emptive therapy. Clearly reporting on patients with a negative outcome should be improved. We have provided guidance how to detect failure early in a clinical setting in accordance to the results from this systematic review.

Study registration: PROSPERO registration number CRD42017077606.

Strengths and limitations of this study

- To reduce selection bias, all the studies were independently reviewed and risk of bias was assessed by two authors and disagreements solved by including third reviewer.
- Studies were included that recruited only adult patients.
- Prospective single-arm studies were included as these studies should record failure of prophylaxis, however these can cause bias.
- To reach a broad scope for the systematic review three databases, MEDLINE, Embase and CENTRAL, were searched.
- The systematic review was reported according to PRISMA guidelines.

For peer review only

INTRODUCTION

In spite of novel immunosuppressive regimens and antimicrobial prophylaxis, infectious complications remain a threat for solid organ and stem cell transplant recipients [1–5]. These patients are especially susceptible to opportunistic infections like cytomegalovirus (CMV), *Pneumocystis jirovecii* pneumonia (PCP), febrile neutropenia, human herpesvirus 6 (HHV-6) and invasive fungal infections (IFI) [1–5]. Graft failure is a major risk of these opportunistic infections [6–8].

Antimicrobial prophylaxis and pre-emptive therapy are used as preventive measures, however these vary notably among different transplant centers [9,10]. This can be explained by differences in local setting and lack of high level evidence in antifungal, antimicrobial and antiviral prophylaxis guidelines and fairly dated evidence for some infections like PCP and febrile neutropenia [5,11–14]. Although prophylaxis has proven to be a beneficial strategy, breakthrough infections and adverse events resulting in stop of prophylaxis occur [15–17]. The rates of failure of prophylaxis have remained around 10-20% for opportunistic infections [15,18–20]. In order to be able to optimize prophylaxis it is important to understand underlying causes for failure. It would also be helpful to explore adverse events in patients that stopped taking the drug.

This systematic review aimed to summarize the main reasons why prophylaxis has failed in solid organ and allogeneic stem cell transplant recipients and how failure is reported in prospective studies.

METHODS

Definition of failure

In this systematic review we have defined *failure of prophylaxis and pre-emptive therapy* as stopping or changing the therapy during the study period for any stated reason. For example, failure could be a breakthrough infection, non-adherence, adverse events leading to stopping of therapy etc. Even if some of the side- effects could be anticipated, if this leads to stopping of therapy, it was defined as failure. We did not look into infections in the post-prophylactic period.

Search strategy and selection criteria

For peer review only - http://bmjopen.bmj.com/site/about/guidelines.xhtml

Methods of the analysis and inclusion criteria were specified in advance and documented in a protocol (PROSPERO ID: CRD42017077606), which is available online: https://www.crd.york.ac.uk/prospero/display_record.php?RecordID=77606 (**Appendix 1**). This report follows the PRISMA checklist (**Appendix 2**) [21]. This systematic review included prospective randomized controlled trials and prospective single-arm studies from Jan 1. 2010 to Oct 1. 2018. There were no limitations for the patient setting, publication status and language. We analysed the failure during prophylaxis and pre-emptive therapy thus the follow-up time varied. The review included patients (16 years and older) who had received either allogeneic stem cell, lung, kidney, liver, heart, pancreas or small bowel transplantation. Moreover, these patients were receiving trimethoprim/sulfamethoxazole (TMP+SMX) for prophylaxis of PCP, ciprofloxacin for prophylaxis of febrile neutropenia, ganciclovir and/or valganciclovir for prophylaxis or preemptive-therapy of CMV and human herpesvirus 6 (HHV-6); or posaconazole or voriconazole or fluconazole or itraconazole for prophylaxis of invasive fungal infections (IFI). Primary outcome measures were breakthrough infections, adverse events leading to stopping of treatment, switching medication or dose reduction. Secondary outcome measures were acquired resistance to antimicrobials, antifungals or antivirals and death.

To identify studies for this systematic review the following databases were searched: MEDLINE (PubMed), EMBASE and Cochrane Central Register of Controlled Trials (CENTRAL). The search was performed on Oct 1. 2018. The search strategy included MeSH terms and variations of transplant types, medications and prophylaxis ("Antibiotic Prophylaxis"[Mesh] OR antimicrobial OR anti-microbial) AND (Host OR Transplants OR Transplantation OR Transplant Recipients OR immunocompromised OR transplant* OR kidney transplant*). The full search strategies of all databases can be found in **Appendix 3.** The searches were done by AGM and MB. For screening we used Covidence software (www.covidence.org).

The literature search and data extraction for inclusion and eligibility for this systematic review were done according to the inclusion criteria by AGM and MB independently. If there were discrepancies between the results these were discussed and resolved with JWCA or with consensus. If there was data missing or additional questions from the selected studies, then the authors of these studies were contacted. We excluded conference proceedings, retrospective studies, reviews, editorials and letters to the editor.

For peer review only

Outcomes, data extraction and quality

Data was extracted by AGM and independently checked by MB. Disagreement between reviewers was resolved by discussion with a third reviewer JWCA. Data was extracted from each included trial on: characteristics of patients, type of intervention, study design, study population, outcome measurement, reasons for failure of prophylaxis or pre-emptive therapy (stopping of prophylaxis/pre-emptive therapy), main conclusions by authors, strengths and limitations (see **Appendix 4**).

We considered performing a meta-analysis for our systematic review, however because the patient cohorts include different transplantations and varied interventions the studies were too heterogeneous, we decided to do a qualitative systematic review. For every RCT for outcomes adverse events, breakthrough infections and death odds ratios were calculated. The studies were divided into CMV and IFI prophylaxis and pre-emptive treatment groups.

Risk of bias in individual studies was assessed independently by the AGM and MB. For assessing bias in individual studies Revised Cochrane risk-of-bias tool for randomized trials (RoB 2) was used for RCTs and Risk of Bias in Non-randomized Studies of Interventions (ROBINS-I) was used for prospective single-arm studies [22,23].

RESULTS

The search identified 3318 records for inclusion in the review. In total 603 duplicate records were removed, and 2543 records were excluded after screening of title and abstract. Full text screening of 171 articles resulted in 24 studies (in 30 articles) (PRISMA flow diagram in **Figure 1**) to be included in the final review. We decided to exclude one full text in Japanese as it would not likely change the outcome of our systematic review. We contacted corresponding authors of 24 studies (30 articles) for further information about reasons for preliminary stopping of prophylaxis and overall adherence to treatment, 7 responded and 3 of those sent pre-specified protocols, however none provided additional information about failure of prophylaxis or pre-emptive treatment. The individual study characteristics and risk of bias are presented in **Table 1**.

For peer review only

We identified 24 studies including 2851 patients. Seventeen studies (11 RCTs [15,24,33–38,25–32] and 6 single-arm [39–46]) with 1952 subjects focused on CMV prophylaxis and seven studies (5 RCTs [17,19,47–49] and 2 single-arm [44,50]) with 899 subjects focused on IFI prophylaxis.

Of the 17 CMV studies eight included only valganciclovir, two only ganciclovir, five included both valganciclovir and ganciclovir, two valaciclovir and valganciclovir. From all 17 CMV studies seven focused only on prophylaxis, six only on preemptive therapy, three both on prophylaxis and pre-emptive therapy and one on prophylaxis and therapy.

The IFI studies varied with regards to study medication and patient group. Three studies evaluated fluconazole (comparison amphotericin B, anidulafungin and micafungin), one posaconazole (comparison amphotericin B lipid complex), one itraconazole (comparison micafungin) and two single-arm studies had voriconazole as study medication.

Failure of prophylaxis and pre-emptive therapy

No specific information about failure during prophylaxis or pre-emptive therapy was given in 25% (6 studies - 4 being RCTs) of the included studies [19,33,36,40,42,47,51]. Four of these studies did record follow-up infections or long-term failure of prophylaxis therapy after cessation of prophylaxis [19,40,42,47] and for one RCT the time-point was not specified[46].

The most common reasons for failure of CMV prophylaxis (1524 study subjects) and CMV pre-emptive therapy (428 study subjects) were adverse events [15,30,35,37,38,42,46] and breakthrough CMV [15,37,41,42,46]. For IFI prophylaxis (899 study subjects), it was adverse events [19,44,48–50] and IFI [17,19,44,49]. Overall the adverse events and breakthrough infections were described in 50% and 38% of the studies respectively. In **Table 2** the reasons for stopping prophylaxis are described in detail. The detailed information about failure in the CMV (1506 study subjects) and IFI (761 study subjects) RCT groups are summarized in **Figure 2** and **3**.

Not all identified cases of failure could be clarified even after contact with the authors of the studies. The reasons for stopping prophylaxis and pre-emptive therapy, being for *patient reasons* or *physician or sponsor decision* [35,37], were not explained in any of these studies. Moreover, in two studies[24,35] it was stated that prophylaxis was stopped because of *other* reasons. Adverse events, breakthrough infections and causes for death were mostly described in more detail in the included studies [15,16,46,48–50,17,25,29,35,37,38,42,44], however the

For peer review only - http://bmjopen.bmj.com/site/about/guidelines.xhtml

reason 'other' [15,35] for stopping or physician/investigator [17,35,37,49] and patients discretion [15] were grouped together in studies and not described in detail.

Results of individual studies

For this systematic review we defined outcomes of failure of prophylaxis – breakthrough infections, adverse events and death. Results are reported in **Table 3**. With regards to our defined outcomes the heterogeneity is high in these studies.

Risk of bias across studies

Risk of bias was assessed using five domains for RCTs: randomization, assignment and adherence to intervention, measurement of outcome, missing data, and selection of the reported results. For single-arm/observational studies seven domains were used: confounding, selection of participants, classification of interventions, missing data, measurement of outcomes and selection of the reported result. We concluded that all 16 RCTs and six single-arm had low risk of bias, three had some concerns and two had high risk of bias. Risk of bias in RCTs and single-arm studies is presented in **Tables 4** and **5**.

DISCUSSION

We aimed to explore the reasons why prophylaxis and pre-emptive therapy failed in transplant recipients. Twenty-four studies were included into this systematic review. We concluded that the main reason for stopping prophylaxis was adverse events for both CMV and IFI prophylaxis and CMV pre-emptive therapy. We did not observe notable differences between the prophylaxis and pre-emptive therapy groups. This result was expected as cessation of ganciclovir therapy is often described due to debilitating side-effects, especially bone marrow suppression [15], which is even more problematic in hematological patients [36]. Different antifungal agents have a diverse safety profile. Our results were also in line with the common side-effects of antifungals as we observed nausea and vomiting in azoles and nephrotoxicity in Amphotericin B as reasons for discontinuation of therapy [43,46,48].

Adverse events are known to be underreported so the numbers that we summarized in this systematic review might be underestimated [52,53]. A review by Golder et al [53] looked into different study designs and reporting of adverse events. The authors outlined that there is wide underreporting of adverse events across different study designs and even more in the

For peer review only - http://bmjopen.bmj.com/site/about/guidelines.xhtml

unpublished data. Unfortunately contacting the authors of the studies included in this systematic review did not result in additional information about the reported adverse events. Meta-analysis was not done as the studies were too heterogeneous.

Surprisingly, reporting of failure was insufficient in 25% of RCTs and 50% of prospective single-arm studies. One fourth of the studies did not report on preliminarily cessation of prophylaxis nor underlying reason. More worrisome was the fact that discontinuation of prophylaxis was mostly explained as adverse events and breakthrough infections, while patient and physicians' discretion as reasons were grouped together. On the other hand, we observed that only five studies did record infections that occurred after the prophylaxis had been stopped. This is concerning, as in a clinical setting and in developing guidelines it is important to make a distinction of breakthrough infections during and after prophylaxis and pre-emptive therapy [54]. Also, it makes it more difficult to compare the efficacy of different medications, for example when using ganciclovir and letermovir [54]. Furthermore, one of the included RCTs [17] reported early discharge from hospital as the main argument for stopping of prophylaxis. In addition, some reasons were not clarified, for example physician and patient discretion were combined in two of the included studies. A systematic review published in 2018 explored the efficacy and safety of CMV prophylaxis; adverse events and breakthrough infections were addressed, however the authors did not explicitly report additional information why patients stopped within these studies [55]. Similarly, in a PCP prophylaxis systematic review the authors described adverse events as reason for discontinuation while not mentioning adherence, resistance or patients' choice [14].

A systematic review about quality of reporting RCTs in medical oncology described that 79% of the adverse events in studies are reported according to CONSORT criteria, although the description of participants and preliminary stopping in each stage of the study was done correctly in only 59% of the studies [56]. It has been argued that perhaps, poor reporting is deliberate to mask the shortcomings in study design [56,57]. One may expect that in a prospective study the patients are recruited and analyzed prospectively thus the data about failure of prophylaxis like adverse events should be readily obtainable. Thus, registering study protocols in clinical trial registers and following reporting guidelines helps consistent and more straightforward reporting of results [58,59]. Goldacre and colleagues have looked into reporting of clinical trials specifically with regards to outcomes. They concluded that most of

For peer review only - http://bmjopen.bmj.com/site/about/guidelines.xhtml

the studies did not have sufficient reporting – either outcomes were added or prespecified outcomes were not reported. [60] These results raise concern to also whether failure is reported as per protocol. In our case, we faced a substantial quantity of missing data on failure and it is not always clear to us how studies were conducted with respect to exclusion of patients.

According to our search criteria we also planned to include studies looking into prophylaxis and pre-emptive therapy of PCP, HHV-6 virus and febrile neutropenia. Surprisingly, we were not able to identify suitable studies looking into prophylaxis of these infections for our systematic review. Current guidelines for the treatment of these infections use case reports, retrospective studies, surveillance studies, and outdated literature to give recommendations [5,61,62]. For instance, a systematic review focusing on PCP prophylaxis in non-HIV immunocompromised patients is used as main guidance of prophylaxis in this patient group, the studies included in this review date back from 1974 to 2008 (13 in the range 1977-1990) [14]. Moreover, the prophylaxis of febrile neutropenia is widely supported by a systematic review that included studies with quinolones published from 1980-2010 (10 in the range 1980-1997) [13]. Half of the studies in these reviews were published more than 20 years ago. Certainly, the evidence from these reviews are relevant to the field, however the landscape of treatment of transplant recipients has changed – notably, with the emergence of resistant pathogens [63–66] and new data in todays' setting is needed to aid the update of clinical guidelines [62].

As mentioned before, detailed data about failure of prophylaxis and pre-emptive therapy was lacking in some studies and no additional information was obtained when reaching out to the authors. Furthermore, one more limitation of our study was the fact that we included studies from year 2010 onwards, however, as mentioned the approach to management of different infections has changed in the past years thus we planned to include the most recent evidence [11,12]. Another limitation is that we did not include studies about children. On the other hand, included studies already showed a heterogeneous variety of patient populations (e.g. autologous and allogeneic SCT recipients [19,48]), various types of infections and prophylaxis and pre-emptive therapy studies; we therefore believe that adding studies on children would have made it more difficult to summarize findings.

For peer review only - http://bmjopen.bmj.com/site/about/guidelines.xhtml

There still remains a variety of different practices between centers that complicates transfer of patients between different hospitals [9,67]. We believe that having information about the discontinuation of failure of prophylaxis and pre-emptive therapy could provide valuable information for guideline committees, medical practitioners and researchers conducting studies with these medications. Without this information similar errors could be repeated in different studies.

There are several ways in to predict failure in a clinical setting. Detecting adverse events and avoiding breakthrough infections can be done through approaching the patient or the treating physician. For example using mobile applications for reporting adverse events[68], defining the high-risk patients [69,70] and therapeutic drug monitoring of potentially toxic medications [71].

In **Figure 4** we have summarized guidance how to detect failure early. As mentioned above there are multiple studies that do not report the true breakthrough infections, only post-prophylaxis infections are reported. In addition, we believe that defining the potential adverse events before and describing the method of measurement of these could increase adherence. Other supporting background from the current systematic review for this figure is presented in **Appendix 5**.

For peer review only

Conclusions

In general, RCTs and prospective single-arm studies about prophylaxis and pre-emptive therapy of opportunistic infections should provide more in-depth information about failure. The main reason why prophylaxis or pre-emptive therapy is stopped are adverse events, however these might well be underreported. Thus, the management and reporting of adverse events is critically important and should be improved in clinical studies. In addition, our results also suggest partially biased approach in the publication of clinical studies and therefore there are insufficient data to support evidence-based decision-making in prophylaxis of PCP, HHV-6 and febrile neutropenia.

Conflicts of interests

There are no competing interests to declare.

Funding

For peer review only - http://bmjopen.bmj.com/site/about/guidelines.xhtml

AGM was funded by Marie Skłodowska-Curie Actions, Grant Agreement number: 713660 — PRONKJEWAIL — H2020-MSCA-COFUND-2015.

Acknowledgements

The authors would like to thank Mariska M.G. Leeflang for her suggestions and help while analyzing and compiling the data of this systematic review.

Author statement

AG Märtson did the screening, writing, data analysis, risk of bias analysis, searches and planning, M Bakker did the screening, searches, data analysis, writing, H Blokzijl, EAM Verschuuren, SP, Berger, LFR Span, TS van der Werf did the planning, writing, reviewing of manuscript, JWC Alffenaar did the writing, analysis, planning.

Data availability statement

Unpublished data is available on request from the authors.

Patient and Public Involvement

This research was done without patient involvement. Patients were not invited to comment on the study design and were not consulted to develop patient relevant outcomes or interpret the results. Patients were not invited to contribute to the writing or editing of this document for readability or accuracy.

For peer review only - http://bmjopen.bmj.com/site/about/guidelines.xhtml

References

1. Ullmann AJ, Schmidt-Hieber M, Bertz H, et al. Infectious diseases in allogeneic haematopoietic stem cell transplantation: prevention and prophylaxis strategy guidelines 2016. *Ann Hematol*. 2016;95(9):1435-1455. doi:10.1007/s00277-016-2711-1

2. Menzin J, Meyers JL, Friedman M, et al. The economic costs to United States hospitals of invasive fungal infections in transplant patients. *Am J Infect Control*. 2011;39(4):e15-e20. doi:https://doi.org/10.1016/j.ajic.2010.06.009

3. Hakimi Z, Aballea S, Ferchichi S, et al. Burden of cytomegalovirus disease in solid organ transplant recipients: a national matched cohort study in an inpatient setting. *Transpl Infect Dis*. 2017;19(5). doi:10.1111/tid.12732

4. Iriart X, Bouar M Le, Kamar N, Berry A. Pneumocystis Pneumonia in Solid-Organ Transplant Recipients. *J fungi (Basel, Switzerland)*. 2015;1(3):293-331. doi:10.3390/jof1030293

5. Taplitz RA, Kennedy EB, Bow EJ, et al. Antimicrobial Prophylaxis for Adult Patients With Cancer-Related Immunosuppression: ASCO and IDSA Clinical Practice Guideline Update. *J Clin Oncol*. September 2018:JCO1800374. doi:10.1200/JCO.18.00374

6. Alsaeed M, Husain S. Infections in Heart and Lung Transplant Recipients. *Crit Care Clin*. 2019;35(1):75-93. doi:10.1016/j.ccc.2018.08.010

7. Freeman RBJ. The “indirect” effects of cytomegalovirus infection. *Am J Transplant*. 2009;9(11):2453-2458. doi:10.1111/j.1600-6143.2009.02824.x

8. Fishman JA. Infection in solid-organ transplant recipients. *N Engl J Med*. 2007;357(25):2601-2614. doi:10.1056/NEJMra064928

9. Manuel O, Kralidis G, Mueller NJ, et al. Impact of antiviral preventive strategies on the incidence and outcomes of cytomegalovirus disease in solid organ transplant recipients. *Am J Transplant*. 2013;13(9):2402-2410. doi:10.1111/ajt.12388

10. Navarro D, San-Juan R, Manuel O, et al. Cytomegalovirus infection management in

For peer review only

For peer review only - http://bmjopen.bmj.com/site/about/guidelines.xhtml

solid organ transplant recipients across European centers in the time of molecular diagnostics: An ESGICH survey. *Transpl Infect Dis*. 2017;19(6). doi:10.1111/tid.12773

11. Patterson TF, Thompson GR 3rd, Denning DW, et al. Practice Guidelines for the Diagnosis and Management of Aspergillosis: 2016 Update by the Infectious Diseases Society of America. *Clin Infect Dis*. 2016;63(4):e1-e60. doi:10.1093/cid/ciw326

12. Kotton CN, Kumar D, Caliendo AM, et al. The Third International Consensus Guidelines on the Management of Cytomegalovirus in Solid Organ Transplantation. *Transplantation*. March 2018. doi:10.1097/TP.0000000000002191

13. Gafter-Gvili A, Fraser A, Paul M, et al. Antibiotic prophylaxis for bacterial infections in afebrile neutropenic patients following chemotherapy. *Cochrane database Syst Rev*. 2012;1:CD004386. doi:10.1002/14651858.CD004386.pub3

14. Stern A, Green H, Paul M, Vidal L, Leibovici L. Prophylaxis for Pneumocystis pneumonia (PCP) in non-HIV immunocompromised patients. *Cochrane database Syst Rev*. 2014;(10):CD005590. doi:10.1002/14651858.CD005590.pub3

15. Humar A, Lebranchu Y, Vincenti F, et al. The efficacy and safety of 200 days valganciclovir cytomegalovirus prophylaxis in high-risk kidney transplant recipients. *Am J Transplant*. 2010;10(5):1228-1237. doi:10.1111/j.1600-6143.2010.03074.x

16. Liu, Amy W, Jutivorakool, Kamonwan, Fisher, Cynthia E., Rakita, Robert M., Reyes, Jorge D., Bhattacharya, Renuka B., Jerome, Keith R., Limaye, Ajit P. Comparison of preemptive therapy and antiviral prophylaxis for prevention of cytomegalovirus in seropositive liver transplant recipients. *Transplantation*. 2018;102(4):632-639. doi:10.1097/TP.0000000000002029 LK - http://rug.on.worldcat.org/atoztitles/link/?sid=EMBASE&issn=00411337&id=doi:10.1097%2FTP.0000000000002029&atitle=Comparison+of+preemptive+therapy+and+antiviral+prophylaxis+for+prevention+of+cytomegalovirus+in+seropositive+liver+transplant+recipients&stitle=Transplantation&title=Transplantation&volume=102&issue=4&spage=632&epage=639&aulast=Liu&aufirst=Amy+W.&auinit=A.W.&aufull=Liu+A.W.&coden=TRPLA&isbn=&pages=632-639&date=2018&auinit1=A&auinitm=W.

17. Winston DJ, Limaye AP, Pelletier S, et al. Randomized, double-blind trial of

For peer review only

anidulafungin versus fluconazole for prophylaxis of invasive fungal infections in high-risk liver transplant recipients. *Am J*. 2014;14(12):2758-2764. doi:10.1111/ajt.12963

18. van der Beek MT, Berger SP, Vossen ACTM, et al. Preemptive versus sequential prophylactic-preemptive treatment regimens for cytomegalovirus in renal transplantation: comparison of treatment failure and antiviral resistance. *Transplantation*. 2010;89(3):320-326. doi:10.1097/TP.0b013e3181bc0301

19. Huang X, Chen H, Han M, et al. Multicenter, Randomized, Open-Label Study Comparing the Efficacy and Safety of Micafungin versus Itraconazole for Prophylaxis of Invasive Fungal Infections in Patients undergoing Hematopoietic Stem Cell Transplant. *Biol Blood Marrow Transplant*. 2012;18(10):1509-1516. doi:10.1016/j.bbmt.2012.03.014

20. Helfrich M, Dorschner P, Thomas K, Stosor V, Ison MG. A retrospective study to describe the epidemiology and outcomes of opportunistic infections after abdominal organ transplantation. *Transpl Infect Dis*. 2017;19(3):e12691. doi:10.1111/tid.12691

21. Liberati A, Altman DG, Tetzlaff J, et al. The PRISMA Statement for Reporting Systematic Reviews and Meta-Analyses of Studies That Evaluate Health Care Interventions: Explanation and ElaborationPRISMA: Explanation and Elaboration. *Ann Intern Med*. 2009;151(4):W-65-W-94. doi:10.7326/0003-4819-151-4-200908180-00136

22. Higgins JPT, Sterne JAC, Savović J, Page MJ, Hróbjartsson A, Boutron I, Reeves B ES. A revised tool for assessing risk of bias in randomized trials In: Chandler J, McKenzie J, Boutron I, Welch V (editors). Cochrane Methods. *Cochrane Database Syst Rev*. 2016;(10 (Suppl 1)).

23. Sterne JAC, Hernán MA, Reeves BC, et al. ROBINS-I: a tool for assessing risk of bias in non-randomised studies of interventions. *BMJ*. 2016;355:i4919. doi:10.1136/bmj.i4919

24. Humar A, Limaye AP, Blumberg EA, et al. Extended valganciclovir prophylaxis in D+/R- kidney transplant recipients is associated with long-term reduction in cytomegalovirus disease: two-year results of the IMPACT study. *Transplantation*. 2010;90(12):1427-

For peer review only

1431.

25. Halim M, Al-Otaibi T, Gheith O, et al. Efficacy and safety of low-dose versus standard-dose valganciclovir for prevention of cytomegalovirus disease in intermediate-risk kidney transplant recipients. *Exp Clin Transplant*. 2016;14(5):526-534. doi:10.6002/ect.2015.0305

26. Gheith O, Halim MA, Al-Otaibi T, et al. Successful cost-effective prevention of cytomegalovirus disease in kidney transplant recipients using low-dose valganciclovir. *Exp Clin Transplant*. 2017;15:156-163. doi:10.6002/ect.mesot2016.P34

27. Witzke O, Hauser I, Bartels M, Wolf G, Wolters H, Nitschke M. Valganciclovir Prophylaxis Versus Preemptive Therapy in Cytomegalovirus-Positive Renal Allograft Recipients: 1-Year Results of a Randomized Clinical Trial. *Transplantation*. 2012;93(1):61-68. doi:10.1097/TP.0b013e318238dab3

28. Witzke O, Nitschke M, Bartels M, et al. Valganciclovir Prophylaxis Versus Preemptive Therapy in Cytomegalovirus-Positive Renal Allograft Recipients: long-term Results after 7 Years of a Randomized Clinical Trial. *Transplantation*. 2018;102(5):876-882. doi:10.1097/TP.0000000000002024

29. Reischig T, Hribova P, Jindra P, et al. Long-term outcomes of pre-emptive valganciclovir compared with valacyclovir prophylaxis for prevention of cytomegalovirus in renal transplantation. *J Am Soc Nephrol*. 2012;23(9):1588-1597. doi:10.1681/ASN.2012010100

30. Reischig T, Kacer M, Jindra P, Hes O, Lysak D, Bouda M. Randomized trial of valganciclovir versus valacyclovir prophylaxis for prevention of cytomegalovirus in renal transplantation. *Clin J Am Soc Nephrol*. 2015;10(2):294-304. doi:10.2215/CJN.07020714

31. Reischig T, Prucha M, Sedlackova L, et al. Valganciclovir prophylaxis against cytomegalovirus impairs lymphocyte proliferation and activation in renal transplant recipients. *Antivir Ther*. 2011;16(8):1227-1235. doi:10.3851/IMP1879

32. Togashi J, Sugawara Y, Hashimoto M, et al. Oral valganciclovir versus intravenous

For peer review only

ganciclovir as preemptive treatment for cytomegalovirus infection after living donor liver transplantation: a randomized trial. *Biosci Trends*. 2011;5(5):217-222. http://onlinelibrary.wiley.com/o/cochrane/clcentral/articles/974/CN-00881974/frame.html.

33. Padulles A, Colom H, Bestard O, et al. Contribution of population pharmacokinetics to dose optimization of ganciclovir-valganciclovir in solid-organ transplant patients. *Antimicrob Agents Chemother*. 2016;60(4):1992-2002. doi:10.1128/AAC.02130-15

34. Finlen CC, Davis W, Snyder L, et al. Long-term efficacy and safety of 12 months of valganciclovir prophylaxis compared with 3 months after lung transplantation: A single-center, long-term follow-up analysis from a randomized, controlled cytomegalovirus prevention trial. *J Hear lung Transplant*. 2011;30(9):990-996. doi:10.1016/j.healun.2011.02.017

35. Palmer S, Limaye A, Banks M, et al. Extended valganciclovir prophylaxis to prevent cytomegalovirus after lung transplantation: a randomized, controlled trial. *Ann Intern Med*. 2010;152(12):761-769. doi:10.7326/0003-4819-152-12-201006150-00003

36. Chawla J, Ghobadi A, Mosley J, et al. Oral valganciclovir versus ganciclovir as delayed pre-emptive therapy for patients after allogeneic hematopoietic stem cell transplant: a pilot trial (04-0274) and review of the literature. *Transpl Infect Dis*. 2012;14(3):259-267. doi:10.1111/j.1399-3062.2011.00689.x

37. Boeckh M, Nichols W, Chemaly R, et al. Valganciclovir for the prevention of complications of late cytomegalovirus infection after allogeneic hematopoietic cell transplantation: A randomized trial. *Ann Intern Med*. 2015;162(1):1-10. doi:10.7326/M13-2729

38. Kim S, Lee M, Kim S, et al. A randomized trial of preemptive therapy for prevention of cytomegalovirus disease after allogeneic hematopoietic stem cell transplantation. *Int J Hematol*. 2010;91(5):886-891. doi:10.1007/s12185-010-0580-2

39. Montejo, M, Porto, M, Carmon, O, Zárraga, S, Gainza, J, Amenabar, JJ, Goikoetxea, J, Bereciartua E LI. Prophylactic therapy with valganciclovir in high-risk (cytomegalovirus D+/R-) kidney transplant recipients: A single-center experience. *Proceedings*.

For peer review only

For peer review only - http://bmjopen.bmj.com/site/about/guidelines.xhtml

2010;42(8):2947-2949. doi:10.1016/j.transproceed.2010.08.017

40. Nanmoku, K, Shinzato, T, Kubo, T, Shimizu, T, Kimura, T, Yagisawa T. Prevention of Late-Onset Cytomegalovirus Infection and Disease in Donor-Positive/Recipient-Negative Kidney Transplant Recipients Using Low-Dose Valganciclovir. *Transplant Proc*. 2018;50(1):124-129. doi:10.1016/j.transproceed.2017.12.025 LK - http://rug.on.worldcat.org/atoztitles/link/?sid=EMBASE&issn=18732623&id=doi:10.1016%2Fj.transproceed.2017.12.025&atitle=Prevention+of+Late-Onset+Cytomegalovirus+Infection+and+Disease+in+Donor-Positive%2FRecipient-Negative+Kidney+Transplant+Recipients+Using+Low-Dose+Valganciclovir&stitle=Transplant.+Proc.&title=Transplantation+Proceedings&volume=50&issue=1&spage=124&epage=129&aulast=Nanmoku&aufirst=K.&auinit=K.&aufull=Nanmoku+K.&coden=TRPPA&isbn=&pages=124

41. Perrottet N, Csajka C, Pascual M, et al. Population Pharmacokinetics of Ganciclovir in Solid-Organ Transplant Recipients Receiving Oral Valganciclovir. *Antimicrob Agents Chemother*. 2009;53(7):3017 LP - 3023. doi:10.1128/AAC.00836-08

42. McGillicuddy JW, Weimert NA, Taber DJ, et al. Can preemptive cytomegalovirus monitoring be as effective as universal prophylaxis when implemented as the standard of care in patients at moderate risk? *Transplantation*. 2010;89(10):1218-1223. doi:10.1097/TP.0b013e3181d54ba6

43. Mitsani D, Nguyen MH, Shields RK, et al. A prospective, observational study of voriconazole therapeutic drug monitoring among lung transplant recipients receiving prophylaxis: Factors impacting levels and associations between serum troughs, efficacy and toxicity. *Antimicrob Agents Chemother*. 2012;56. doi:10.1128/AAC.05219-11

44. Cordonnier C, Rovira M, Maertens J, et al. Voriconazole for secondary prophylaxis of invasive fungal infections in allogeneic stem cell transplant recipients: results of the VOSIFI study. *Haematologica*. 2010;95(10):1762-1768. doi:10.3324/haematol.2009.020073

45. Takenaka, K, Nagafuji, K, Takase, K, Kamimura, T, Mori, Y, Ito, Y, Nishi, Y, Henzan, H,

For peer review only

For peer review only - http://bmjopen.bmj.com/site/about/guidelines.xhtml

Kato, K, Harada, N, Eto, T, Miyamoto, T, Teshima, T, Akashi K. Initial low-dose valganciclovir as a preemptive therapy is effective for cytomegalovirus infection in allogeneic hematopoietic stem cell transplant recipients. *Int J Hematol*. 2012;96(1):94-100. doi:10.1007/s12185-012-1087-9

46. Park S-Y, Lee S-O, Choi S-H, et al. Efficacy and safety of low-dose ganciclovir preemptive therapy in allogeneic haematopoietic stem cell transplant recipients compared with conventional-dose ganciclovir: a prospective observational study. *J Antimicrob Chemother*. 2012;67(6):1486-1492. doi:10.1093/jac/dks043

47. Perrella A, Esposito C, Pisaniello D, et al. Role of liposomal amphotericin B prophylaxis after liver transplantation compared with fluconazole for high-risk patients. impact on infections and mortality within one year. *Transplant Proc*. 2012;44(7):1977-1981. doi:10.1016/j.transproceed.2012.06.013

48. Chaftari A-M, Hachem RY, Ramos E, et al. Comparison of posaconazole versus weekly amphotericin B lipid complex for the prevention of invasive fungal infections in hematopoietic stem-cell transplantation. *Transplantation*. 2012;94(3):302-308. doi:10.1097/TP.0b013e3182577485

49. Park S, Kim K, Jang JH, et al. Randomized trial of micafungin versus fluconazole as prophylaxis against invasive fungal infections in hematopoietic stem cell transplant recipients. *J Infect*. 2016;73(5):496-505. doi:10.1016/j.jinf.2016.06.011

50. Mitsani D, Nguyen MH, Shields RK, et al. Prospective, observational study of voriconazole therapeutic drug monitoring among lung transplant recipients receiving prophylaxis: Factors impacting levels of and associations between serum troughs, efficacy, and toxicity. *Antimicrob Agents Chemother*. 2012;56(5):2371-2377. doi:10.1128/AAC.05219-11

51. Manuel O, Kralidis G, Mueller NJ, et al. Impact of antiviral preventive strategies on the incidence and outcomes of cytomegalovirus disease in solid organ transplant recipients. *Am J Transplant*. 2013;13(9):2402-2410. doi:10.1111/ajt.12388

52. Hazell L, Shakir SAW. Under-Reporting of Adverse Drug Reactions. *Drug Saf*. 2006;29(5):385-396. doi:10.2165/00002018-200629050-00003

For peer review only

For peer review only - http://bmjopen.bmj.com/site/about/guidelines.xhtml

53. Golder S, Loke YK, Wright K, Norman G. Reporting of Adverse Events in Published and Unpublished Studies of Health Care Interventions: A Systematic Review. *PLoS Med*. 2016;13(9):e1002127-e1002127. doi:10.1371/journal.pmed.1002127

54. Chen K, Cheng MP, Hammond SP, Einsele H, Marty FM. Antiviral prophylaxis for cytomegalovirus infection in allogeneic hematopoietic cell transplantation. *Blood Adv*. 2018;2(16):2159-2175. doi:10.1182/bloodadvances.2018016493

55. Gagelmann N, Ljungman P, Styczynski J, Kroger N. Comparative Efficacy and Safety of Different Antiviral Agents for Cytomegalovirus Prophylaxis in Allogeneic Hematopoietic Cell Transplantation: A Systematic Review and Meta-Analysis. *Biol Blood Marrow Transplant*. May 2018. doi:10.1016/j.bbmt.2018.05.017

56. You B, Maillet D, Chen EX, et al. Quality of Reporting of Modern Randomized Controlled Trials in Medical Oncology: A Systematic Review. *JNCI J Natl Cancer Inst*. 2012;104(13):982-989. doi:10.1093/jnci/djs259

57. Huwiler-Muntener K, Juni P, Junker C, Egger M. Quality of reporting of randomized trials as a measure of methodologic quality. *JAMA*. 2002;287(21):2801-2804.

58. Alley AB, Seo J-W, Hong S-T. Reporting results of research involving human subjects: an ethical obligation. *J Korean Med Sci*. 2015;30(6):673-675. doi:10.3346/jkms.2015.30.6.673

59. Schulz KF, Altman DG, Moher D. CONSORT 2010 Statement: updated guidelines for reporting parallel group randomised trials. *BMJ*. 2010;340:c332. doi:10.1136/bmj.c332

60. Ben Goldacre, Henry Drysdale, Anna Powell-Smith, Aaron Dale, Ioan Milosevic, Eirion Slade, Philip Hartley, Cicely Marston, Kamal Mahtani CH. The COMPare Trials Project.

61. Betts BC, Young J-AH, Ustun C, Cao Q, Weisdorf DJ. Human Herpesvirus 6 Infection after Hematopoietic Cell Transplantation: Is Routine Surveillance Necessary? *Biol Blood Marrow Transplant*. 2011;17(10):1562-1568. doi:10.1016/j.bbmt.2011.04.004

62. Cooley L, Dendle C, Wolf J, et al. Consensus guidelines for diagnosis, prophylaxis and management of Pneumocystis jirovecii pneumonia in patients with haematological

For peer review only

578 and solid malignancies, 2014. *Intern Med J*. 2014;44(12b):1350-1363.
579 doi:10.1111/imj.12599

580 63. Ivulich S, Westall G, Dooley M, Snell G. The Evolution of Lung Transplant
581 Immunosuppression. *Drugs*. 2018;78(10):965-982. doi:10.1007/s40265-018-0930-6

582 64. Krisl JC, Doan VP. Chemotherapy and Transplantation: The Role of
583 Immunosuppression in Malignancy and a Review of Antineoplastic Agents in Solid
584 Organ Transplant Recipients. *Am J Transplant*. 2017;17(8):1974-1991.
585 doi:10.1111/ajt.14238

586 65. Soderlund C, Radegran G. Immunosuppressive therapies after heart transplantation--
587 The balance between under- and over-immunosuppression. *Transplant Rev (Orlando)*.
588 2015;29(3):181-189. doi:10.1016/j.trre.2015.02.005

589 66. Baron D, Giral M, Brouard S. Reconsidering the detection of tolerance to individualize
590 immunosuppression minimization and to improve long-term kidney graft outcomes.
591 *Transpl Int*. 2015;28(8):938-959. doi:10.1111/tri.12578

592 67. Hand J. Strategies for Antimicrobial Stewardship in Solid Organ Transplant Recipients.
593 *Infect Dis Clin North Am*. 2018;32(3):535-550. doi:10.1016/j.idc.2018.04.003

594 68. de Vries ST, Denig P, Lasheras Ruiz C, et al. Interest in a Mobile App for Two-Way Risk
595 Communication: A Survey Study Among European Healthcare Professionals and
596 Patients. *Drug Saf*. 2018;41(7):697-712. doi:10.1007/s40264-018-0648-0

597 69. Vallejo C, Vazquez L, Cabrera Martin JR, et al. Treatment of invasive fungal infections
598 in high-risk haematological patients: what have we learnt in the past 10 years? *Rev*
599 *Esp Quimioter*. 2013;26(4):378-386.

600 70. Freeman RB, Cohen JT. Transplantation Risks and the Real World: What Does 'High
601 Risk' Really Mean? *Am J Transplant*. 2009;9(1):23-30. doi:10.1111/j.1600-
602 6143.2008.02476.x

603 71. Roberts JA, Abdul-Aziz MH, Lipman J, et al. Individualised antibiotic dosing for patients
604 who are critically ill: challenges and potential solutions. *Lancet Infect Dis*.
605 2014;14(6):498-509. doi:https://doi.org/10.1016/S1473-3099(14)70036-2

For peer review only

72. Larizgoitia I, Bouesseau M-C, Kelley E. WHO Efforts to Promote Reporting of Adverse Events and Global Learning. *J Public health Res*. 2013;2(3):e29-e29. doi:10.4081/jphr.2013.e29

73. Razonable RR, Hayden RT. Clinical utility of viral load in management of cytomegalovirus infection after solid organ transplantation. *Clin Microbiol Rev*. 2013;26(4):703-727. doi:10.1128/CMR.00015-13

74. Boch T, Spiess B, Cornely O, et al. Diagnosis of invasive fungal infections in haematological patients by combined use of galactomannan, 1,3-beta-D-glucan, Aspergillus PCR, multifungal DNA-microarray, and Aspergillus azole resistance PCRs in blood and bronchoalveolar lavage samples: results. *Clin Microbiol Infect*. 2016;22(10):862-868. doi:10.1016/j.cmi.2016.06.021

75. Patzer RE, Serper M, Reese PP, et al. Medication understanding, non-adherence, and clinical outcomes among adult kidney transplant recipients. *Clin Transplant*. 2016;30(10):1294-1305. doi:10.1111/ctr.12821

76. Zanetti-Yabur A, Rizzo A, Hayde N, Watkins AC, Rocca JP, Graham JA. Exploring the usage of a mobile phone application in transplanted patients to encourage medication compliance and education. *Am J Surg*. 2017;214(4):743-747. doi:https://doi.org/10.1016/j.amjsurg.2017.01.026

For peer review only

Figure 1. PRISMA diagram

Figure 2. Failure of CMV prophylaxis in 11 RCTs (y-axis represents number of subjects and x-axis represents transplant).

Figure 3. Failure of IFI prophylaxis in 5 RCTs (y-axis represents number of subjects and x-axis represents transplant).

Figure 4. Detecting failure in a clinical setting [68–76] (Appendix 5)

For peer review only

648 **Table 1.** Study characteristics

Author, year	Country	Study design	Population	Preemptive therapy/ prophylaxis	Prophylaxis/preemptive medication	Comparison	Sample size	Duration	Follow-up	Summary assessment of risk of bias
**CYTOMEGALVIRUS PROPHYLAXIS/PREEMPTIVE THERAPY**										
**Humar et al, 2010[1], 2010[2]**	Australia, Belgium, Canada, Brazil, France, Germany, the UK, Italy, Poland, Romania, Spain, USA	RCT	Kidney transplant recipients aged 17-77 years	Prophylaxis	Valganciclovir	Valganciclovir different duration and placebo	320 patients	100 and 200 days	12 months	Low risk
**Halim et al, 2016 Gheith et al, 2017**	Kuwait, Egypt	RCT	Kidney transplant recipients with mean age of 42.4 years	Prophylaxis	Valganciclovir	Valganciclovir different duration	201 patients	98.1 days (mean)	12 months	Some concerns
**Witzke et al 2012, 2018**	Germany, Austria	RCT	Kidney transplant recipients with mean age of 52.7 years	Prophylaxis and pre-emptive therapy	Valganciclovir	Valganciclovir as preemptive treatment	296 patients	100 days (14 days preemptive until 100 days)	7 years	Some concerns
**Reischig et al 2012**	Czeck Republic	RCT	Kidney transplant	Prophylaxis and pre-	Valganciclovir	Valacyclovir	70 patients	3 months	2 years	Some concerns

For peer review only - http://bmjopen.bmj.com/site/about/guidelines.xhtml

			recipients with mean age of 49 years	emptive therapy						
**Reischig et al 2011, 2015**	Czeck Republic	RCT	Kidney transplant recipients with mean age of 49 years	Prophylaxis	Valganciclovir	Valacyclovir	119 patients	3 months	1 year	Some concerns
**Togashi et al 2011**	Japan	RCT	Liver transplant recipients with mean age of 52.1 years	Preemptive therapy	Valganciclovir	Ganciclovir	22 patients	2 weeks	1 year	Some concerns
**Padulles et al 2016**	Spain	RCT	Kidney, liver and heart transplant recipients with mean age of 53.9 years	Prophylaxis and treatment	Valganciclovir/ Ganciclovir	Valganciclovir /Ganciclovir different dosing	53 patients	NA (not reported)	6 months	Low risk
**Palmer et al 2010 Finlen Copeland et al 2011**	USA	RCT	Lung transplant recipients with median age of 55.5 years	Prophylaxis	Valganciclovir	Placebo	136 patients	12 months	3.9 years (mean)	Some concerns
**Chawla et al 2011**	USA	RCT	Allogeneic transplant recipients aged 18-64	Preemptive therapy	Valganciclovir	Ganciclovir	37 patients	28 days	4 weeks	High risk
**Boeckh et al 2015**	USA	RCT	Allogeneic transplant recipients	Preemptive therapy	Valganciclovir	Placebo	184 patients	150/120 days valganciclo	640 days	Some concerns

For peer review only - http://bmjopen.bmj.com/site/about/guidelines.xhtml

			aged 16-70					vir/placebo (medians)		
**Kim et al 2010**	South Korea	RCT	Allogeneic transplant recipients aged 16-49	Preemptive therapy	Ganciclovir	Ganciclovir different dosing	68 patients	14 days (median)	42 months (median)	Some concerns
**Montejo et al 2010**	Spain	Single-arm	Kidney transplant recipients with median age 47 years	Prophylaxis	Valganciclovir	NA	34 patients	3 months	1 year	Critical risk
**Nanmoku et al 2018**	Japan	Single-arm	Kidney transplant recipients with mean age 48.7 years	Prophylaxis	Valganciclovir	NA	100 patients	6 months	1 year	Serious risk
**Perrottet et al 2009 Manuel et al 2010**	France	Single-arm	Kidney , heart, lung, liver transplant recipients aged 18-70	Prophylaxis	Valganciclovir and Ganciclovir	NA	65 patients	3 months	1 year	Serious risk
**McGillicudy et al 2010**	USA	Single-arm	Kidney and pancreas transplant recipients mean age 53.5 years	Preemptive therapy and prophylaxis	Valganciclovir/ ganciclovir	Valganciclovir /ganciclovir preemptive therapy	130 patients	90 days	1 year	Serious risk
**Takenaka et al 2012**	Japan	Single-arm	Allogeneic transplant recipients aged 25-68	Preemptive therapy	Valganciclovir	NA	20 patients	2 weeks	10 weeks	Serious risk
**Park et al 2012**	South Korea	Single-arm	Allogeneic transplant	Preemptive therapy	Ganciclovir	Ganciclovir different	97 patients	14 days (median)	Not same for all	Moderate risk

For peer review only - http://bmjopen.bmj.com/site/about/guidelines.xhtml

			recipients aged 16-70			dosing			patients	
**INVASIVE FUNGAL INFECTION PROPHYLAXIS**										
**Parrella et al 2012**	Italy	RCT	Liver transplant recipients (age NI)	Prophylaxis	Fluconazole	Amphotericin B	43 patients	7-14 days	1 year	Some concerns
**Winston et al 2014**	USA	RCT	Liver transplant recipients aged 19-75 years	Prophylaxis	Fluconazole	Anidulafungin	200 patients	21 days (median)	90 days	Low risk
**Chaftari et al 2012**	USA	RCT	Allogeneic transplant recipients aged 20-69	Prophylaxis	Posaconazole	Amphotherici n B lipid complex	40 patients	6 weeks	2 weeks	Some concerns
**Huang et al 2012**	China	RCT	Allogeneic and autologous transplant recipients with mean age 32.7 years	Prophylaxis	Itraconazole	Micafungin	228 patients	42 days	4 weeks	Some concerns
**Park et al 2016**	South Korea	RCT	Allogeneic and autologous transplant recipients aged 20-64	Prophylaxis	Fluconazole	Micafungin	250 patients	21 days	100 days	Some concerns
**Mitsani et al 2012**	USA	Single-arm	Lung transplant recipients aged 20-74 years	Prophylaxis	Voriconazole	NA	93 patients	3 months	NI	Moderate risk

For peer review only - http://bmjopen.bmj.com/site/about/guidelines.xhtml

**Cordonnier et al 2010**	France, Spain, Belgium, UK, Germany, Switzerland, Portugal, Sweden	Single-arm	Allogeneic transplant recipients aged 22-72 years	Prophylaxis	Voriconazole	NA	45 patients	94 days (median)	1 year	Critical risk

RCT – randomized controlled trial, NA – not applicable, NI – no information

For peer review only

For peer review only - http://bmjopen.bmj.com/site/about/guidelines.xhtml

Table 2. Reasons for stopping prophylaxis or pre-emptive therapy

**Reasons for stopping/failing prophylaxis or pre-emptive therapy**	
**Cytomegalovirus prophylaxis/pre-emptive therapy**	
Humar et al, 2010	27 AE,<br>51 development of CMV disease<br>11 refused treatment,<br>15 other reasons
Halim et al, 2016	3 died (acute myocardial infarction, recurrent oxalosis, severe rejection and renal vein thrombosis)
Witzke et al 2012	NI (12-month tolerability described)
Reischig et al 2012	Valganciclovir:<br>10 lost the graft,<br>2 died
Reischig et al 2015	Valganciclovir:<br>4 lost the graft,<br>3 died,<br>1 moved to another country,<br>13 AE (leukopenia and neutropenia)
Togashi et al 2011	All patients completed treatment protocol.
Padulles et al 2016	No breakthrough infections reported<br>1 AE
Palmer et al 2010	3 withdrew consent,<br>7 died,<br>17 AE,<br>15 physician's discretion,<br>3 other reasons
Chawla et al 2011	NI
Boeckh et al 2015	33 CMV breakthrough infection,<br>2 CMV disease,<br>44 adverse events,<br>20 investigator/sponsor discretion
Kim et al 2010	1 AE,<br>2 died (neutropenic infection)
Montejo et al 2010	3 CMV infection

peer review

Nanmoku et al 2018	NI
Perrottet et al 2009	3 CMV viremia (<100 copies/ML)
McGillicudy et al 2010	3 AE (leukopenia) during prophylaxis,<br>1 CMV colitis in pre-emptive group<br>NI for during prophylaxis
Takenaka et al 2012	All patients completed treatment protocol, no dosage changes were made.
Park et al 2012	8 AE (neutropenia, nephrotoxicity),<br>12 dose reductions,<br>6 CMV disease (time-point not confirmed),<br>37 deaths (time-point not confirmed)
**Invasive fungal infection prophylaxis**	
Perrella et al 2012	NI
Winston et al 2014	Fluconazole<br>36 discharge from hospital (preliminary stopping of prophylaxis),<br>1 died,<br>2 physician's discretion,<br>2 IFIs
Chaftari et al 2012	Posaconazole<br>8 AE
Huang et al 2012	Itraconazole (allogeneic and autologous SCT):<br>14 IFIs,<br>2 AE (hemolysis, gastrointestinal disorders),<br>9 investigator's decision
Park et al 2016	Fluconazole (allogeneic and autologous SCT)<br>3 IFIs,<br>2 AE (diarrhoea, bilirubin elevation),<br>3 investigator's discretion
Mitsani et al 2012	25 AE (nausea/vomiting, hepatotoxicity, central nervous system events)
Cordonnier et al 2010	2 AE (hepatotoxicity),<br>3 IFIs (during 6 months – not all during prophylaxis),<br>11 died (time-point not confirmed)

For peer review only - http://bmjopen.bmj.com/site/about/guidelines.xhtml

AE – adverse event, NI – no information (not reported), IFI – invasive fungal infection, CMV - cytomegalovirus

Table 3. RCTs with defined failures

Study	Events	Total	Events comparator	Total	Odds ratio [95% CI]
**1. Adverse events**					
1.1 CMV					
Palmer et al 2010	6	66	11	70	0.54 [0.19, 1.54]
Humar et al 2010	17	156	10	164	1.90 [0.84, 4.28]
Boeckh et al 2015	29	95	15	89	2.17 [1.07, 4.39]
Reischig et al 2015 (Valgan/Valacy)	13	60	5	59	2.99 [0.99, 9.00]
Kim et al 2010	1	32	0	36	3.48 [0.14, 88.40]
1.2 IFI					
Chaftari et al 2012 (Posa/Amph B)	8	21	15	19	0.16 [0.04, 0.67]
Park et al 2016 (Fluc/Mica)	2	85	3	165	1.30 [0.21, 7.94]
**2. Breakthrough infections**					
2.1 CMV					
Boeckh et al 2015	6	95	29	89	0.14 [0.05, 0.36]
Humar et al 2010	4	156	47	164	0.07 [0.02, 0.19]
2.2 IFI					
Winston et al 2014 (Fluc/Anid, ITT population)	5	99	2	98	2.55 [0.48, 13.49]
Park et al 2016 (Fluc/Mica)	3	85	6	165	0.97 [0.24, 3.98]
**3. Death**					
3.1 CMV					
Palmer et al 2010	3	66	4	70	0.79 [0.17, 3.65]

For peer review only - http://bmjopen.bmj.com/site/about/guidelines.xhtml

Reischig et al 2015 (Valgan/Valacy)	0	60	3	59	0.13 [0.01, 2.64]
Reischig et al 2012	0	36	2	34	0.18 [0.01, 3.85]
Halim et al 2016	1	100	2	101	0.50 [0.04, 5.60]
Kim et al 2010	2	32	0	36	5.98 [0.28, 129.44]
3.2 IFI					
Winston et al 2014 (Fluc/Anid)	3	100	4	100	0.74 [0.16, 3.41]

Table 4. Risk of bias in RCTs using RoB tool

	Randomization process	Assignment of intervention	Adhering to intervention	Measurement of outcomes	Missing outcome data	Selection of the reported result
Humar et al 2010	+	+	+	+	+	?
Halim et al 2016	?	+	+	?	+	?
Witzke et al 2012	+	+	+	?	+	?
Reischig et al 2012	+	+	+	?	+	?
Reischig et al 2015	+	+	+	?	+	?
Togashi et al 2011	?	+	+	?	+	?
Padulles et al 2016	+	+	+	+	+	?
Palmer et al 2010	+	+	?	+	+	?
Chawla et al 2011	?	?	?	?	+	?
Boeckh et al 2015	+	+	?	+	+	?
Kim et al 2010	?	+	+	?	+	?
Perrella et al 2012	?	?	?	?	+	?
Winston et al 2014	+	+	+	+	+	?
Chaftari et al 2012	+	?	?	?	+	?
Huang et al 2012	+	+	?	?	+	?
Park et al 2016	?	+	+	?	+	?

+ low risk, - high risk, ? some concerns

For peer review only

Table 5. Risk of bias in single-arm studies using ROBINs tool

	Confounding	Selection of participants	Classification of interventions	Deviations from intended interventions	Missing data	Measurement of outcomes	Selection of the reported result
Montejo et al 2010	-	?	+	NI	-	?	NI
Nanmoku et al 2018	*	+	+	+	+	?	NI
Perrottett et al 2009	*	?	+	?	*	?	NI
McGillicuddy et al 2010	*	+	+	*	*	?	NI
Takenaka et al 2012	*	?	+	?	*	?	NI
Park et al 2012	?	+	+	?	+	?	NI
Mitsani et al 2012	*	?	+	?	?	?	NI
Cordonnier et al 2010	*	*	+	?	?	?	NI

+ low risk,? moderate risk, *serious risk, - critical risk

NI = no information

For peer review only

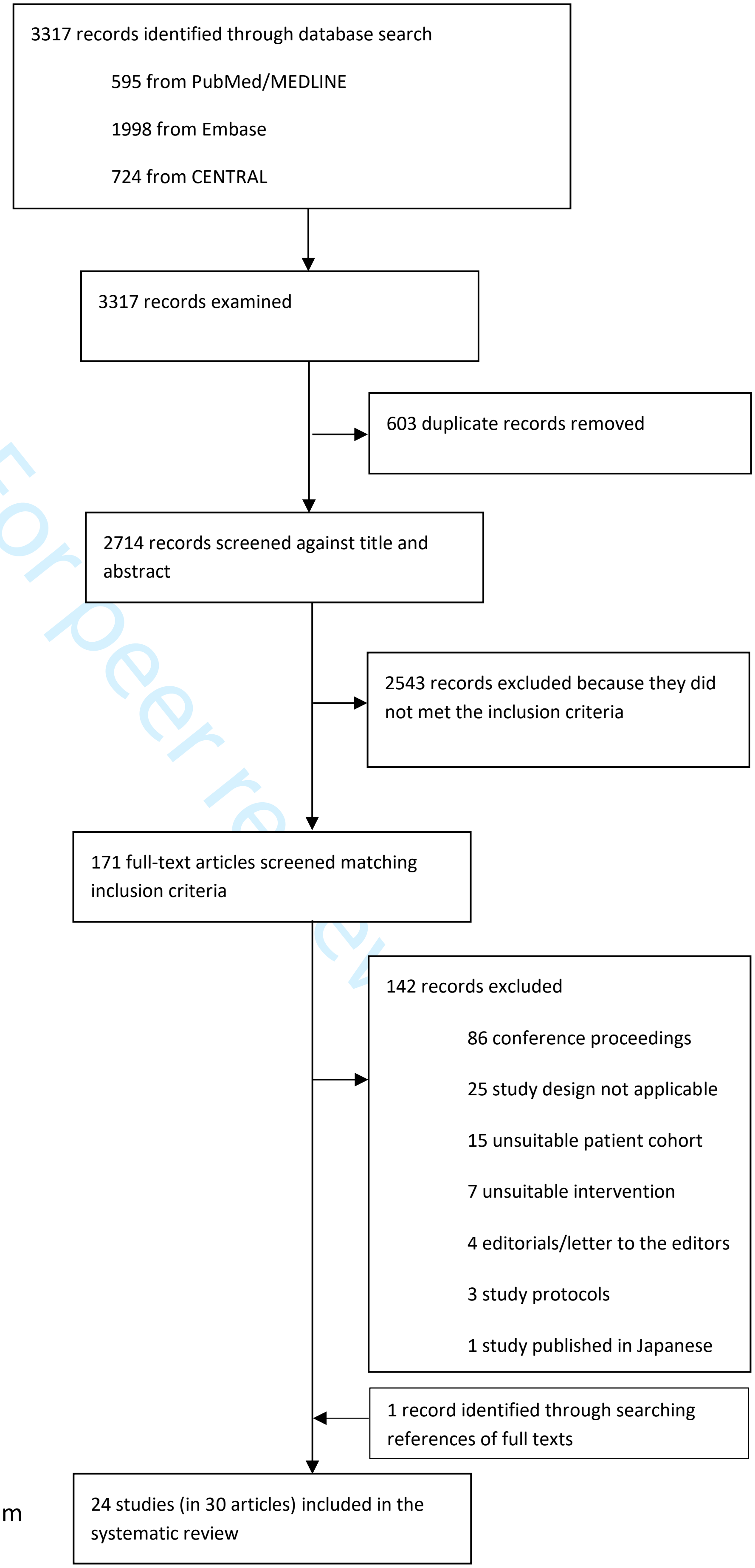

Figure 1. PRISMA diagram

For peer review only - http://bmjopen.bmj.com/site/about/guidelines.xhtml

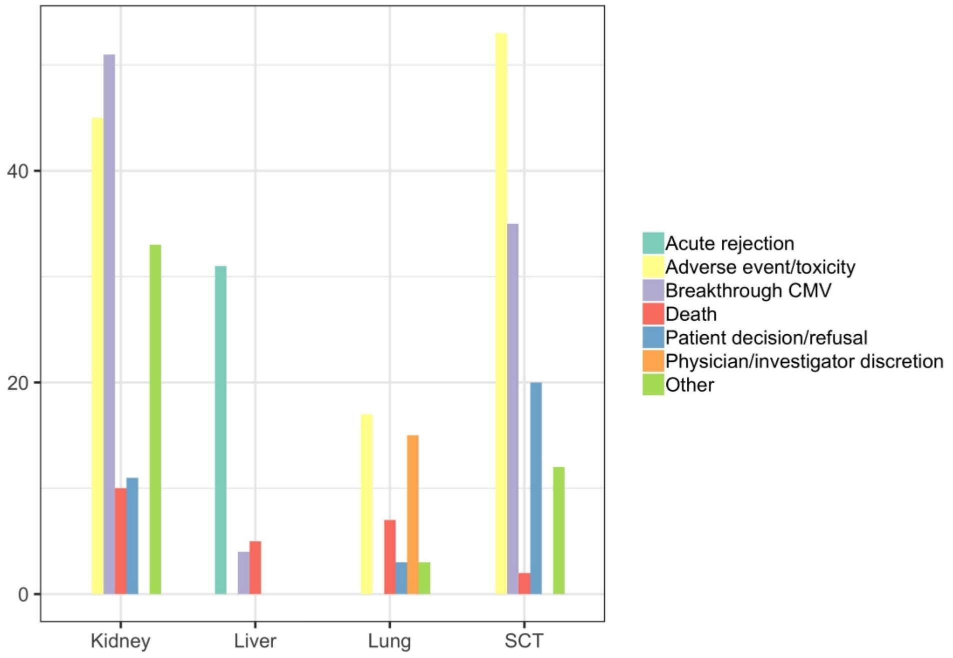

Figure 2. Failure of CMV prophylaxis in 11 RCTs (y-axis represents number of subjects and x-axis represents transplant).

810x563mm (96 x 96 DPI)

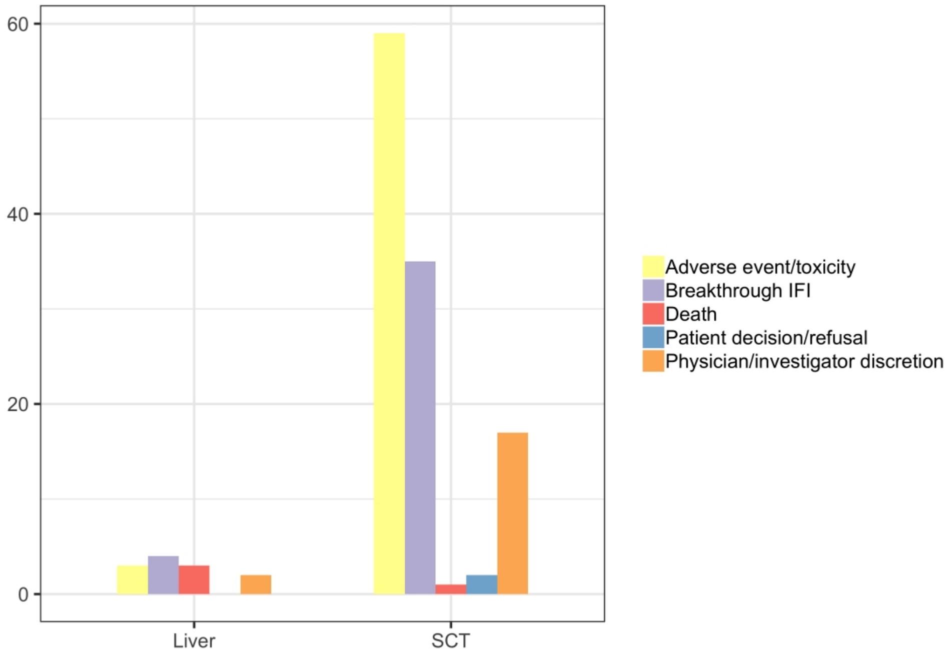

Figure 3. Failure of IFI prophylaxis in 5 RCTs (y-axis represents number of subjects and x-axis represents transplant).

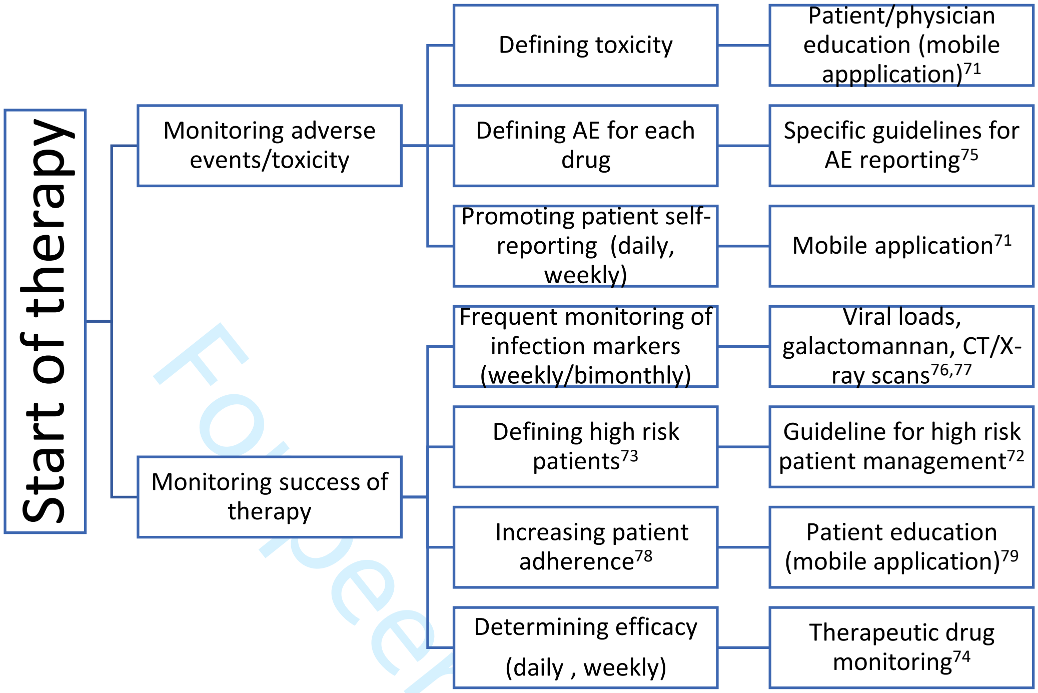

Figure 4. Detecting failure in a clinical setting [68–76] (Appendix 5)

For peer review only

724 **Appendix 1. Study protocol (PROSPERO CRD42017077606)**

PROSPERO International prospective register of systematic reviews

Review title and timescale

1 Review title

Exploring failure of antimicrobial prophylaxis and pre-emptive therapy for transplant recipients: a systematic review

2 Original language title

Not applicable

3 Anticipated or actual start date

01 October 2017

4 Anticipated completion date

09 February 2019

Review team details

5 Named contact

Anne-Grete Märtson

6 Named contact email

a.martson@umcg.nl

7 Named contact address

Dept. of Clinical Pharmacy and Pharmacology; EB 70 UMCG Hanzeplein 1, PO BOX 30001, 9700 RB Groningen the Netherlands

8 Named contact phone number

+37253325121

9 Organisational affiliation of the review

University Medical Center Groningen

Website address: umcg.nl

10 Review team members and their organisational affiliations

	Anne-Grete	Märtson	UMCG, the Netherlands
Dr	Martijn	Bakker	UMCG, the Netherlands
Prof.Dr.	Tjip.S.	van der Werf	UMCG, the Netherlands

For peer review only

Dr	Jan-Willem	Alffenaar	UMCG, the Netherlands

11 Funding sources/sponsors

This systematic review will be part of a PhD thesis that is funded by University Medical Centre Groningen and the European Union’s Horizon 2020 research and innovation programme Under the Marie Skłodowska-Curie grant agreement 713660.

12 Conflicts of interest

There are no actual or potential conflicts of interest.

13 Collaborators

Title	First name	Last name	Organisation details
Dr	Hans	Blokzijl	UMCG, the Netherlands
Dr	Erik A.M.	Verschuuren	UMCG, the Netherlands
Dr	L.F.R.	Span	UMCG, the Netherlands
Dr	Stefan P.	Berger	UMCG, the Netherlands

Review methods

14 Review question(s)

It is widely known that solid organ and allogenic stem cell transplant patients are especially susceptible to opportunistic infections like Cytomegalovirus (CMV), *Pneumocystis jirovecii* pneumonia (PCP) and invasive fungal infections. In recent years, organ transplantation has developed greatly. Transplantation has become available for a bigger patient population and this has led to a wider range of infections after transplantation. The concern with appropriate prophylactic regimens remain as antifungal prophylaxis varies exceptionally among different transplant centres.

To prevent these infections from occurring different prophylactic strategies are used before and after transplantation like ganciclovir and valganciclovir for CMV and trimethoprim-sulfamethoxazole (TMP+SMX) for PCP. This has proven to be a beneficial approach, despite that there is still a proportion of patients whose prophylactic treatment fails as they develop breakthrough infections, suffer from adverse events or

For peer review only

simply do not tolerate medications.

This systematic review is aiming to summarise the main reasons why prophylaxis is failed for non-HIV immunocompromised patients looking at prospective studies done on this topic.

The objectives of this study are to review and analyse literature to determine which factors might contribute to failure of prophylaxis of opportunistic infections in transplant recipients.

The potential factors for failure are drug-drug interactions, microbiological susceptibility, drug pharmacokinetics, the dose of the medication, route of administration, transplant (specific organ or allogenic stem cell), status of the immunocompromised patient and medication compliance.

15 Searches

This systematic review will include prospective randomized controlled trials and prospective single-arm studies. There are no restrictions for the setting or language. Studies published from 1.01.2010 to present will be included to the systematic review.

To identify studies for this systematic review these databases will be searched: MEDLINE, EMBASE, Cochrane Central Register of Controlled Trials (CENTRAL) and PubMed.

16 URL to search strategy

PubMed search strategy

("Immunity, Cellular"[Mesh] OR "Immunocompromised Host"[Mesh] OR "Transplants"[Mesh] OR "Transplantation"[Mesh] OR immunocompromised [tiab] OR transplant*[tiab])

AND

("valganciclovir" [Supplementary Concept] OR "Ganciclovir"[Mesh] OR "Trimethoprim, Sulfamethoxazole Drug Combination"[Mesh] OR "Ciprofloxacin"[Mesh:NoExp] OR "posaconazole" [Supplementary Concept] OR "Voriconazole"[Mesh] OR "Fluconazole"[Mesh] OR valganciclovir [tiab] OR Ganciclovir [tiab] OR Trimethoprim [tiab] OR Sulfamethoxazole [tiab] OR Ciprofloxacin [tiab] OR posaconazole [tiab] OR Voriconazole [tiab] OR Fluconazole [tiab] OR cotrimoxazole [tiab)

NOT (HIV OR Human immunodeficiency virus)

AND

("Randomized Controlled Trial" [Publication Type] OR randomi*[tiab] OR randomly[tiab] OR trial[ti] OR prospective [tiab] OR single arm [tiab])

For peer review only - http://bmjopen.bmj.com/site/about/guidelines.xhtml

17 Condition or domain being studied

Prophylaxis for opportunistic infections in transplant recipients.

18 Participants/population

The study will include adult patients (16 years and older) who have received:

- allogeneic stem cell transplant
- lung transplant
- kidney transplant
- liver transplant
- heart transplant
- pancreas transplant
- small bowel transplant

19 Intervention(s), exposure(s)

All patients who have received prophylaxis or preemptive therapy for:

- PCP with TMP+SMX
- febrile neutropenia with ciprofloxacin
- cytomegalovirus and human herpesvirus type 6 with ganciclovir and/or valganciclovir
- invasive fungal infections with posaconazole and/or voriconazole and/or fluconazole and/or itraconazole

20 Comparator(s)/control

Placebo or active comparator.

21 Types of study to be included

This systematic review will include prospective randomized controlled trials and prospective single-arm studies.

22 Context

There are no restrictions for the setting.

23 Primary outcome(s)

Primary outcomes:

- adverse effects leading to stopping of treatment or switching medication or dose reduction

For peer review only

- breakthrough infection

24 Secondary outcomes

Secondary outcomes:

- resistance to antimicrobials/antifungals/antivirals
- death

25 Data extraction (selection and coding)

The literature search and data extraction for inclusion and eligibility for this systematic review will be done according to the inclusion criteria by two authors independently. If there are discrepancies between the results these will be discussed and resolved with a third author.

If there is data missing or additional questions from the selected studies then the authors of these studies will be contacted directly.

A form will be developed to document the characteristics and quality of the included studies.

26 Risk of bias (quality) assessment

Risk of bias in individual studies will be assessed independently by the two researchers who did the study selection and data extraction. For assessing bias in individual studies Revised Cochrane risk-of-bias tool for randomized trials (RoB 2) will be used for RCTs and Risk of Bias in Non-randomized Studies of Interventions (ROBINS-I) will be used for prospective single-arm studies

If there are discrepancies between the authors these will be discussed and resolved with a third author.

27 Strategy for data synthesis

The criteria how the data will be quantitatively synthesised and if and how subgroup analysis will be done can be determined after the data is available.

The possibility of performing a meta-analysis and summary measures can also be determined after literature search and when we have done the data extraction.
After the preliminary searches we expect that there will not be sufficient data to conduct a meta-analysis.

28 Analysis of subgroups or subsets

The possibility and method for subgroup analysis can be determined after the data is available and

For peer review only

extracted.

If subgroup analysis is applicable then the subgroups could be different transplant types.

Review general information

29 Type and method of review

Systematic review

30 Language

English

31 Country

The Netherlands

32 Other registration details

Not applicable

33 Reference and/or URL for published protocol

Not applicable

34 Dissemination plans

This systematic review will be submitted to a journal specific to this field.

35 Keywords

systematic review; immunocompromised patients; prophylaxis; antimicrobials; antifungals; antivirals; PCP; CMV; febrile neutropenia; invasive fungal infection; opportunistic infection; transplantation

36 Details of any existing review of the same topic by the same authors

There has not been a previous systematic review done on this topic before.

37 Current review status

Conpleted (not published)

38 Any additional information

This review is part of planning further retrospective or prospective studies on prophylactic regimens for immunocompromised patients.

39 Details of final report/publication(s)

Not applicable

For peer review only

Appendix 2. PRISMA 2009 Checklist

Section/topic	#	Checklist item	Reported on page #
**TITLE**			
Title	1	Identify the report as a systematic review, meta-analysis, or both.	1
**ABSTRACT**			
Structured summary	2	Provide a structured summary including, as applicable: background; objectives; data sources; study eligibility criteria, participants, and interventions; study appraisal and synthesis methods; results; limitations; conclusions and implications of key findings; systematic review registration number.	2
**INTRODUCTION**			
Rationale	3	Describe the rationale for the review in the context of what is already known.	3
Objectives	4	Provide an explicit statement of questions being addressed with reference to participants, interventions, comparisons, outcomes, and study design (PICOS).	3
**METHODS**			
Protocol and registration	5	Indicate if a review protocol exists, if and where it can be accessed (e.g., Web address), and, if available, provide registration information including registration number.	3
Eligibility criteria	6	Specify study characteristics (e.g., PICOS, length of follow-up) and report characteristics (e.g., years considered, language, publication status) used as criteria for eligibility, giving rationale.	4
Information sources	7	Describe all information sources (e.g., databases with dates of coverage, contact with study authors to identify additional studies) in the search and date last searched.	4
Search	8	Present full electronic search strategy for at least one database, including any limits used, such that it could be repeated.	4, 46-47
Study selection	9	State the process for selecting studies (i.e., screening, eligibility, included in systematic review, and, if applicable, included in the meta-analysis).	4
Data collection process	10	Describe method of data extraction from reports (e.g., piloted forms, independently, in duplicate) and any processes for obtaining and confirming data from investigators.	4-5
Data items	11	List and define all variables for which data were sought (e.g., PICOS, funding sources) and any assumptions and simplifications made.	4

For peer review only

Risk of bias in individual studies	12	Describe methods used for assessing risk of bias of individual studies (including specification of whether this was done at the study or outcome level), and how this information is to be used in any data synthesis.	5
Summary measures	13	State the principal summary measures (e.g., risk ratio, difference in means).	5
Synthesis of results	14	Describe the methods of handling data and combining results of studies, if done, including measures of consistency (e.g., I^2) for each meta-analysis.	NA

Section/topic	#	Checklist item	Reported on page #
Risk of bias across studies	15	Specify any assessment of risk of bias that may affect the cumulative evidence (e.g., publication bias, selective reporting within studies).	5
Additional analyses	16	Describe methods of additional analyses (e.g., sensitivity or subgroup analyses, meta-regression), if done, indicating which were pre-specified.	5
**RESULTS**			
Study selection	17	Give numbers of studies screened, assessed for eligibility, and included in the review, with reasons for exclusions at each stage, ideally with a flow diagram.	23
Study characteristics	18	For each study, present characteristics for which data were extracted (e.g., study size, PICOS, follow-up period) and provide the citations.	27-31
Risk of bias within studies	19	Present data on risk of bias of each study and, if available, any outcome level assessment (see item 12).	7, 35-36
Results of individual studies	20	For all outcomes considered (benefits or harms), present, for each study: (a) simple summary data for each intervention group (b) effect estimates and confidence intervals, ideally with a forest plot.	33-36
Synthesis of results	21	Present results of each meta-analysis done, including confidence intervals and measures of consistency.	NA
Risk of bias across studies	22	Present results of any assessment of risk of bias across studies (see Item 15).	35-36
Additional analysis	23	Give results of additional analyses, if done (e.g., sensitivity or subgroup analyses, meta-regression [see Item 16]).	5-7
**DISCUSSION**			
Summary of evidence	24	Summarize the main findings including the strength of evidence for each main outcome; consider their relevance to key groups (e.g., healthcare providers, users, and policy makers).	7-10
Limitations	25	Discuss limitations at study and outcome level (e.g., risk of bias), and at review-level (e.g., incomplete retrieval of identified research, reporting bias).	9-10
Conclusions	26	Provide a general interpretation of the results in the context of other evidence, and implications for future research.	10

For peer review only

FUNDING			
Funding	27	Describe sources of funding for the systematic review and other support (e.g., supply of data); role of funders for the systematic review.	10

From: Moher D, Liberati A, Tetzlaff J, Altman DG, The PRISMA Group (2009). Preferred Reporting Items for Systematic Reviews and Meta-Analyses: The PRISMA Statement. PLoS Med 6(7): e1000097. doi:10.1371/journal.pmed1000097

For more information, visit: **www.prisma-statement.org**.

For peer review only

Appendix 3. Search strategy of EMBASE and CENTRAL

MEDLINE (PubMed) search strategy

("Immunity, Cellular"[Mesh] OR "Immunocompromised Host"[Mesh] OR "Transplants"[Mesh] OR "Transplantation"[Mesh] OR "Transplant Recipients"[Mesh] OR immunocompromised [tiab] OR kidney transplant* [tiab] OR heart transplant* [tiab] OR pancreas transplant* [tiab] OR lung transplant* [tiab] OR stem cell transplant* [tiab] OR small bowel transplant* [tiab] OR liver transplant* [tiab] OR small-bowel transplant* [tiab] OR allogeneic stem cell transplant* [tiab] OR transplant*[tiab])

AND

("valganciclovir" [Supplementary Concept] OR "Antibiotic Prophylaxis"[Mesh] OR "Ganciclovir"[Mesh] OR "Trimethoprim, Sulfamethoxazole Drug Combination"[Mesh] OR "Ciprofloxacin"[Mesh:NoExp] OR "posaconazole" [Supplementary Concept] OR "Voriconazole"[Mesh] OR "Fluconazole"[Mesh] OR "Anti-Infective Agents"[Mesh] OR valganciclovir [tiab] OR Ganciclovir [tiab] OR Trimethoprim [tiab] OR Sulfamethoxazole [tiab] OR Ciprofloxacin [tiab] OR posaconazole [tiab] OR Voriconazole [tiab] OR Fluconazole [tiab] OR bactrim [tiab] OR biseptol [tiab] OR ciprinol [tiab] OR antimicrobial [tiab] OR antibacterial [tiab] OR anti-microbial [tiab] anti-bacterial [tiab] OR anti-fungal [tiab] OR antifungal [tiab] OR antiviral [tiab] OR anti-viral [tiab] OR itraconazole [tiab] OR cotrimoxazole [tiab) NOT (HIV OR Human immunodeficiency virus)

AND

("Randomized Controlled Trial" [Publication Type] OR "Randomized Controlled Trials as Topic"[Mesh] OR randomi*[tiab] OR randomly[tiab] OR trial[tiab] OR prospective [tiab] OR single arm [tiab).

EMBASE search strategy

('immunocompromised patient'/exp OR 'transplantation'/exp OR transplant*:ab,ti OR 'graft recipient'/exp OR immunocompromised:ab,ti OR ‘kidney transplant*’:ab,ti OR ‘heart

For peer review only

transplant*’:ab,ti OR ‘pancreas transplant*’:ab,ti OR ‘lung transplant*’:ab,ti OR ‘small bowel transplant*’:ab,ti OR ‘liver transplant*’:ab,ti OR ‘small-bowel transplant*’:ab,ti OR ‘allogeneic stem cell transplant*’:ab,ti OR ‘stem cell transplant*’:ab,ti)

AND

('valganciclovir'/exp OR 'ganciclovir'/exp OR 'cotrimoxazole'/exp OR 'ciprofloxacin'/exp OR 'posaconazole'/exp OR 'voriconazole'/exp OR 'fluconazole'/exp OR 'itraconazole'/exp OR valganciclovir:ab,ti OR ganciclovir:ab,ti OR trimethoprim:ab,ti OR sulfamethoxazole:ab,ti OR ciprofloxacin:ab,ti OR posaconazole:ab,ti OR voriconazole:ab,ti OR itraconazole:ab,ti OR fluconazole:ab,ti OR bactrim:ab,ti OR biseptol:ab,ti OR ciprinol:ab,ti) NOT (HIV OR Human immunodeficiency virus) AND

('randomized controlled trial'/exp OR 'prospective study'/exp OR randomi*:ab,ti OR randomly:ab,ti OR trial:ab,ti OR prospective:ab,ti OR ‘single arm’:ab,ti)

CENTRAL search strategy

(Immunocompromised Host OR Transplants OR Transplantation OR Transplant Recipients OR immunocompromised OR transplant* OR kidney transplant* OR heart transplant* OR pancreas transplant* OR lung transplant* OR stem cell transplant* OR small bowel transplant* OR liver transplant* OR small-bowel transplant* OR allogeneic stem cell transplant*)

AND

(valganciclovir OR Antibiotic Prophylaxis OR Ganciclovir OR Trimethoprim, Sulfamethoxazole Drug Combination OR Ciprofloxacin OR posaconazole OR Voriconazole OR Fluconazole OR Anti-Infective Agents OR Trimethoprim OR Sulfamethoxazole OR bactrim OR biseptol OR ciprinol OR itraconazole OR antimicrobial OR antibacterial OR anti-microbial or anti-bacterial OR anti-fungal OR antifungal OR antiviral OR anti-viral OR cotrimoxazole) NOT (HIV OR Human immunodeficiency virus)

AND (Randomized Controlled Trial OR randomi* OR randomly OR trial OR prospective OR single arm)

For peer review only

Appendix 4. Data extraction form

- Reference (First author / Year / Title / Journal / Country)

- Study design
  - RCT
  - Single-arm study

- Sample size
  - Number of subjects started study
  - Number of subjects finished study

- Duration of study
  - Prophylaxis/preemptive therapy duration
  - Follow-up duration

- Study population
  - Age range
  - Transplanted organ
  - Underlying diseases

- Intervention
  - Medication used for prohylaxis
  - Comparison if applicable

- Main outcome measures
  - Primary outcomes
  - Secondary outcomes

- Failure of prophylaxis

- Risk of bias
  - RoB or ROBINS risk of bias score – low/high/some concerns

- Strengths and limitations of study

- Key conclusions of authors

For peer review only

Appendix 5. Background of detection of failure in the included studies

Table 1. RCT study reporting

	Humar 2010[1,2]	Halim 2016, Gheith 2017	Witzke 2012, 2018	Reischig 2012	Reichig 2011, 2015	Togashi 2011	Reference<br>Padulles 2016	RCT<br>Palmer 2010 Finlen Copeland 2011	Chawla 2011	Boeckh 2015	Kim 2010	Parrella 2012	Wintston 2014	Chaftari 2012	Huang 2012	Park 2016
**Description of high risk patients**	*	*	*	*	*		*	*	*	*	*	*	*	*		*
**Patient adherence reported**	*	*	*	*	*	*	*	*		*					*	*
**AE/toxicity described/ reported**	*	*	*	*	*	*	*	*	*	*	*		*	*#	*	
**Definition of breakthrough infection**	*	*	*	*	*	*	*	*	PT	PT	PT	*	*	*	*	*
**Breakthrough infection reported**	*	*	*	*	*	*	*	*	PT	PT	PT		*	*	*	*
**Insufficient reporting of topics above mentioned in limitations**						*										

PT – pre-emptive therapy, not applicable

# - toxicity explicitly defined, measurement described

For peer review only - http://bmjopen.bmj.com/site/about/guidelines.xhtml

Table 2. Single-arm study reporting

Topic				Reference	Single-arm			
	Montejo 2010	Nanmoku 2018	Perrottet 2009 Manuel 2010	McGillicuddy 2010	Takenaka 2012	Park 2012	Mitsani 2012	Cordonnier 2010
Description of high risk patients	*	*	*	*	*	*		
Patient adherence reported								*
AE/toxicity described/ reported	*		*#	*	*	*		*
Definition of breakthrough infection	*	*	*	PT	PT	PT	*	*
Breakthrough infection reported	*			PT	PT	PT	*	*
Insufficient reporting of failure mentioned in limitations								

PT – pre-emptive therapy, not applicable

# - toxicity explicitly defined, measurement described

For peer review only - http://bmjopen.bmj.com/site/about/guidelines.xhtml

PRISMA 2009 Checklist

Section/topic	#	Checklist item	Reported on page #
**TITLE**			
Title	1	Identify the report as a systematic review, meta-analysis, or both.	1
**ABSTRACT**			
Structured summary	2	Provide a structured summary including, as applicable: background; objectives; data sources; study eligibility criteria, participants, and interventions; study appraisal and synthesis methods; results; limitations; conclusions and implications of key findings; systematic review registration number.	2
**INTRODUCTION**			
Rationale	3	Describe the rationale for the review in the context of what is already known.	3
Objectives	4	Provide an explicit statement of questions being addressed with reference to participants, interventions, comparisons, outcomes, and study design (PICOS).	3
**METHODS**			
Protocol and registration	5	Indicate if a review protocol exists, if and where it can be accessed (e.g., Web address), and, if available, provide registration information including registration number.	3
Eligibility criteria	6	Specify study characteristics (e.g., PICOS, length of follow-up) and report characteristics (e.g., years considered, language, publication status) used as criteria for eligibility, giving rationale.	4
Information sources	7	Describe all information sources (e.g., databases with dates of coverage, contact with study authors to identify additional studies) in the search and date last searched.	4
Search	8	Present full electronic search strategy for at least one database, including any limits used, such that it could be repeated.	4, 45-46
Study selection	9	State the process for selecting studies (i.e., screening, eligibility, included in systematic review, and, if applicable, included in the meta-analysis).	4
Data collection process	10	Describe method of data extraction from reports (e.g., piloted forms, independently, in duplicate) and any processes for obtaining and confirming data from investigators.	4-5
Data items	11	List and define all variables for which data were sought (e.g., PICOS, funding sources) and any assumptions and simplifications made.	4
Risk of bias in individual studies	12	Describe methods used for assessing risk of bias of individual studies (including specification of whether this was done at the study or outcome level), and how this information is to be used in any data synthesis.	5
Summary measures	13	State the principal summary measures (e.g., risk ratio, difference in means).	5
Synthesis of results	14	Describe the methods of handling data and combining results of studies, if done, including measures of consistency (e.g., I^2) for each meta-analysis.	NA

For peer review only - http://bmjopen.bmj.com/site/about/guidelines.xhtml

PRISMA 2009 Checklist

Section/topic	#	Checklist item	Reported on page #
Risk of bias across studies	15	Specify any assessment of risk of bias that may affect the cumulative evidence (e.g., publication bias, selective reporting within studies).	5
Additional analyses	16	Describe methods of additional analyses (e.g., sensitivity or subgroup analyses, meta-regression), if done, indicating which were pre-specified.	5
**RESULTS**			
Study selection	17	Give numbers of studies screened, assessed for eligibility, and included in the review, with reasons for exclusions at each stage, ideally with a flow diagram.	23
Study characteristics	18	For each study, present characteristics for which data were extracted (e.g., study size, PICOS, follow-up period) and provide the citations.	27-31
Risk of bias within studies	19	Present data on risk of bias of each study and, if available, any outcome level assessment (see item 12).	7, 35-36
Results of individual studies	20	For all outcomes considered (benefits or harms), present, for each study: (a) simple summary data for each intervention group (b) effect estimates and confidence intervals, ideally with a forest plot.	33-36
Synthesis of results	21	Present results of each meta-analysis done, including confidence intervals and measures of consistency.	NA
Risk of bias across studies	22	Present results of any assessment of risk of bias across studies (see Item 15).	35-36
Additional analysis	23	Give results of additional analyses, if done (e.g., sensitivity or subgroup analyses, meta-regression [see Item 16]).	5-7
**DISCUSSION**			
Summary of evidence	24	Summarize the main findings including the strength of evidence for each main outcome; consider their relevance to key groups (e.g., healthcare providers, users, and policy makers).	7-10
Limitations	25	Discuss limitations at study and outcome level (e.g., risk of bias), and at review-level (e.g., incomplete retrieval of identified research, reporting bias).	9-10
Conclusions	26	Provide a general interpretation of the results in the context of other evidence, and implications for future research.	10
**FUNDING**			
Funding	27	Describe sources of funding for the systematic review and other support (e.g., supply of data); role of funders for the systematic review.	10

From: Moher D, Liberati A, Tetzlaff J, Altman DG, The PRISMA Group (2009). Preferred Reporting Items for Systematic Reviews and Meta-Analyses: The PRISMA Statement. PLoS Med 6(7): e1000097. doi:10.1371/journal.pmed1000097

For more information, visit: **www.prisma-statement.org**.

For peer review only - http://bmjopen.bmj.com/site/about/guidelines.xhtml

BMJ Open

Exploring failure of antimicrobial prophylaxis and pre-emptive therapy for transplant recipients: a systematic review

Journal:	*BMJ Open*
Manuscript ID	bmjopen-2019-034940.R1
Article Type:	Original research
Date Submitted by the Author:	29-Nov-2019
Complete List of Authors:	Märtson, Anne-Grete; UMCG, Clinical Pharmacy and Pharmacology<br>Bakker, Martijn; UMCG, Hematology<br>Blokzijl, Hans; Universitair Medisch Centrum Groningen, Gastroenterology and Hepatology<br>Verschuuren, Erik; Universitair Medisch Centrum Groningen, Pulmonary Disease and Tuberculosis<br>Berger, Stefan; Universitair Medisch Centrum Groningen, Nephrology<br>Span, Lambert; UMCG, Hematology<br>van der Werf, Tjip S.; University Medical Center Groningen, Department of Pulmonary Diseases & Tuberculosis<br>Alffenaar, Jan-Willem; Rijksuniversiteit Groningen,
**Primary Subject Heading**:	Infectious diseases
Secondary Subject Heading:	Medical publishing and peer review
Keywords:	Transplant medicine < INTERNAL MEDICINE, INFECTIOUS DISEASES, Infection control < INFECTIOUS DISEASES

SCHOLARONE™
Manuscripts

Exploring failure of antimicrobial prophylaxis and pre-emptive therapy for transplant recipients: a systematic review

Running title: Failure of antimicrobial prophylaxis

Anne-Grete Märtson[1], Martijn Bakker[2], Hans Blokzijl[3], Erik A. M. Verschuuren[4], Stefan P. Berger[5], Lambert F. R. Span[2], Tjip S. van der Werf[4,5], Jan-Willem C. Alffenaar[1,6]*

[1.] University of Groningen, University Medical Center Groningen, Department of Clinical Pharmacy and Pharmacology, Groningen, The Netherlands.

[2.] University of Groningen, University Medical Center Groningen, Department of Hematology, Groningen, The Netherlands.

[3.] University of Groningen, University Medical Center Groningen, Department of Gastroenterology and Hepatology, Groningen, The Netherlands.

[4.] University of Groningen, University Medical Center Groningen, Department of Pulmonary Diseases and Tuberculosis, Groningen, The Netherlands.

[5.] University of Groningen, University Medical Center Groningen, Department of Internal Medicine, Groningen, The Netherlands.

[6.] University of Sydney, Faculty of Medicine and Health, Sydney Pharmacy School, and Westmead Hospital, Sydney Australia

Keywords: antimicrobial prophylaxis, pre-emptive therapy, transplant recipients, opportunistic infections, failure

*Address of correspondence:

Jan-Willem C. Alffenaar
University of Groningen, University Medical Center Groningen
Department of Clinical Pharmacy and Pharmacology
Po box 30.001
9700 RB Groningen
The Netherlands
Email: j.w.c.alffenaar@umcg.nl
Tel: +31 503614070
Fax: +31 503614087

Word count: 3090

For peer review only

Abstract

Objectives

Infections remain a threat for solid organ and stem cell transplant recipients. Antimicrobial prophylaxis and pre-emptive therapy have improved survival of these patients however the failure rates of prophylaxis are not negligible. The aim of this systematic review is to explore the reasons behind failure of antimicrobial prophylaxis and pre-emptive therapy.

Setting

This systematic review included prospective randomized controlled trials (RCT) and prospective single-arm studies.

Participants

The studies included were on prophylaxis and pre-emptive therapy of opportunistic infections in transplant recipients. Studies were included from databases MEDLINE, CENTRAL and Embase published until October 1st 2018.

Primary and secondary outcome measures

Primary outcome measures were breakthrough infections, adverse events leading to stopping of treatment, switching medication or dose reduction. Secondary outcome measures were acquired resistance to antimicrobials, antifungals or antivirals and death.

Results

From 3317 identified records, 30 records from 24 studies with 2851 patients were included in the systematic review. Seventeen focused on prophylactic and pre-emptive treatment of cytomegalovirus (CMV) and seven studies on invasive fungal infection (IFI). The main reasons for failure of prophylaxis and pre-emptive therapy were adverse events and breakthrough infections, which were described in 54% (13 studies) and 38% (9 studies) of the included studies respectively. In 25%, 6 of the studies, a detailed description of patients that experienced failure of prophylaxis or pre-emptive therapy was unclear or lacking.

Conclusions

For peer review only

Our results show that although failure is reported in the studies the level of detail prohibits a detailed analysis of failure of prophylaxis and pre-emptive therapy. Clearly reporting on patients with a negative outcome should be improved. We have provided guidance on how to detect failure early in a clinical setting in accordance to the results from this systematic review.

Study registration: PROSPERO registration number CRD42017077606.

Strengths and limitations of this study

- To reduce selection bias, all the studies were independently reviewed and risk of bias was assessed by two authors and disagreements solved by including a third reviewer.
- One limitation of this systematic review is that the included studies were recruiting only adult patients.
- Inclusion of single-arm studies could be a potential limitation as these can cause bias in the systematic review.
- To reach a broad scope for the systematic review three databases, MEDLINE, Embase and CENTRAL, were searched.
- The systematic review was reported according to PRISMA guidelines.

For peer review only

INTRODUCTION

In spite of novel immunosuppressive regimens and antimicrobial prophylaxis, infectious complications remain a threat for solid organ and stem cell transplant recipients [1–5]. These patients are especially susceptible to opportunistic infections like cytomegalovirus (CMV), *Pneumocystis jirovecii* pneumonia (PCP), febrile neutropenia, human herpesvirus 6 (HHV-6) and invasive fungal infections (IFI) [1–5]. Graft failure is a major risk of these opportunistic infections [6–8]. In recent years, organ transplantation and immunosuppressive regimes have developed greatly and thus become available for a wider patient population. This requires adequate antimicrobial prophylaxis guidelines and studies supporting the scientific evidence.

Antimicrobial prophylaxis and pre-emptive therapy are used as preventive measures, however these vary notably among different transplant centers [9,10]. This can be explained by differences in local setting and lack of high level evidence in antifungal, antimicrobial and antiviral prophylaxis guidelines and fairly dated evidence for some infections like PCP and febrile neutropenia [5,11–14]. Although prophylaxis has proven to be a beneficial strategy, breakthrough infections and adverse events resulting in discontinuation of prophylaxis occur [15–17]. The rates of failure of prophylaxis have remained around 10-20% for opportunistic infections [15,18–20]. In order to be able to optimize prophylaxis it is important to understand underlying causes for failure. This systematic review will identify 1) causes of failure of prophylactic treatment, 2) factors that might contribute to failure of prophylaxis and 3) different approaches for administering prophylaxis.

This systematic review aimed to summarize the main reasons why prophylaxis and pre-emptive therapy has failed in solid organ and allogeneic stem cell transplant recipients and how failure is reported in prospective studies. In this review we focus on failure of prophylactic therapy, during the treatment window.

METHODS

Definition of failure

In this systematic review we have defined *failure of prophylaxis and pre-emptive therapy* as stopping or changing the therapy during the study period for any stated reason. For example,

For peer review only - http://bmjopen.bmj.com/site/about/guidelines.xhtml

failure could be a breakthrough infection, non-adherence, adverse events leading to stopping of therapy etc. Even if some of the side- effects could be anticipated, if this leads to stopping of therapy, it was defined as failure. We did not look into infections in the post-prophylactic period.

Search strategy and selection criteria

Methods of the analysis and inclusion criteria were specified in advance and documented in a protocol (PROSPERO ID: CRD42017077606), which is available online: https://www.crd.york.ac.uk/prospero/display_record.php?RecordID=77606 (**Appendix 1**). This report follows the PRISMA checklist (**Appendix 2**) [21]. This systematic review included prospective randomized controlled trials and prospective single-arm studies from January 1st 2010 to October 1st 2018. The starting date was January 1st 2010 due to the changes in management of different infections in recent decades and to include the most recent evidence[11,12]. There were no limitations for the patient setting, publication status and language. We analysed the failure during prophylaxis and pre-emptive therapy thus the follow-up time varied. The review included patients (16 years and older) who had received either allogeneic stem cell, lung, kidney, liver, heart, pancreas or small bowel transplantation. Studies done on children under 16 years were not included as this would have introduced more variability and require a separate analysis, thus were out with the scope of this review. Moreover, these patients were receiving trimethoprim/sulfamethoxazole (TMP+SMX) for prophylaxis of PCP, ciprofloxacin for prophylaxis of febrile neutropenia, ganciclovir and/or valganciclovir for prophylaxis or preemptive-therapy of CMV and human herpesvirus 6 (HHV-6); or posaconazole or voriconazole or fluconazole or itraconazole for prophylaxis of invasive fungal infections (IFI). Primary outcome measures were breakthrough infections, adverse events leading to stopping of treatment, switching medication or dose reduction. Secondary outcome measures were acquired resistance to antimicrobials, antifungals or antivirals and death.

To identify studies for this systematic review the following databases were searched: MEDLINE (PubMed), EMBASE and Cochrane Central Register of Controlled Trials (CENTRAL). The search was performed on October 1st 2018. The search strategy included MeSH terms and variations of transplant types, medications and prophylaxis ("Antibiotic Prophylaxis"[Mesh] OR

For peer review only - http://bmjopen.bmj.com/site/about/guidelines.xhtml

antimicrobial OR anti-microbial) AND (Host OR Transplants OR Transplantation OR Transplant Recipients OR immunocompromised OR transplant* OR kidney transplant*). The full search strategies of all databases can be found in **Appendix 3.** The searches were done by AGM and MB. For screening we used Covidence software (www.covidence.org).

The literature search and data extraction for inclusion and eligibility for this systematic review were done according to the inclusion criteria by AGM and MB independently. If there were discrepancies between the results these were discussed and resolved with JWCA or with consensus. If there was data missing or additional questions from the selected studies, then the authors of these studies were contacted. We excluded conference proceedings, retrospective studies, reviews, editorials and letters to the editor.

Outcomes, data extraction and quality

Data was extracted by AGM and independently checked by MB. Disagreement between reviewers was resolved by discussion with a third reviewer JWCA. Data was extracted from each included trial on: characteristics of patients, type of intervention, study design, study population, outcome measurement, reasons for failure of prophylaxis or pre-emptive therapy (stopping of prophylaxis/pre-emptive therapy), main conclusions by authors, strengths and limitations (see **Appendix 4**).

We considered performing a meta-analysis for our systematic review, however because the patient cohorts include different transplantations and varied interventions the studies were too heterogeneous, we decided to do a qualitative systematic review. The studies were divided into CMV and IFI prophylaxis and pre-emptive treatment groups.

Risk of bias in individual studies was assessed independently by AGM and MB. For assessing bias in individual studies Revised Cochrane risk-of-bias tool for randomized trials (RoB 2) was used for RCTs and Risk of Bias in Non-randomized Studies of Interventions (ROBINS-I) was used for prospective single-arm studies [22,23].

RESULTS

The search identified 3318 records for inclusion in the review. In total 603 duplicate records were removed, and 2543 records were excluded after screening of title and abstract. Full text screening of 171 articles resulted in 24 studies (in 30 articles) (PRISMA flow diagram in **Figure**

For peer review only - http://bmjopen.bmj.com/site/about/guidelines.xhtml

1) to be included in the final review. We decided to exclude one full text in Japanese as it would not likely change the outcome of our systematic review. We contacted corresponding authors of 24 studies (30 articles) for further information about reasons for preliminary stopping of prophylaxis and overall adherence to treatment, 7 responded and 3 of those sent pre-specified protocols, however none provided additional information about failure of prophylaxis or pre-emptive treatment. The individual study characteristics and risk of bias are presented in **Table 1**.

We identified 24 studies including 2851 patients. Seventeen studies (11 RCTs [15,24–38] and 6 single-arm [39–46]) with 1952 subjects focused on CMV prophylaxis and 7 studies (5 RCTs [17,19,47–49] and 2 single-arm [44,50]) with 899 subjects focused on IFI prophylaxis.

Of the 17 CMV studies 8 included only valganciclovir, 2 only ganciclovir, 5 included both valganciclovir and ganciclovir, 2 valaciclovir and valganciclovir. From all 17 CMV studies 7 focused only on prophylaxis, 6 only on preemptive therapy, 3 both on prophylaxis and pre-emptive therapy and 1 on prophylaxis and therapy.

The IFI studies varied with regards to study medication and patient group. Three studies evaluated fluconazole (comparison amphotericin B, anidulafungin and micafungin), 1 posaconazole (comparison amphotericin B lipid complex), 1 itraconazole (comparison micafungin) and 2 single-arm studies had voriconazole as study medication.

<u>Failure of prophylaxis and pre-emptive therapy</u>

No specific information about failure during prophylaxis or pre-emptive therapy was given in 25% (6 studies - 4 being RCTs) of the included studies [19,25,28,40,42,47,51]. Four of these studies did record follow-up infections or long-term failure of prophylaxis therapy after cessation of prophylaxis [19,40,42,47] and for 1 RCT the time-point was not specified[46].

The most common reasons for failure of CMV prophylaxis (1524 study subjects) and CMV pre-emptive therapy (428 study subjects) were adverse events [15,25,27,29,30,36,42,46] and breakthrough CMV [15,29,41,42,46]. For IFI prophylaxis (899 study subjects), it was adverse events [19,44,48–50] and IFI [17,19,44,49]. Overall the adverse events and breakthrough infections were described in 54% (13 studies) and 38% (9 studies) of the studies respectively. In **Table 2** the reasons for stopping

For peer review only

prophylaxis are described in detail. The detailed information about failure in the CMV (1506 study subjects) and IFI (761 study subjects) RCT groups are summarized in **Figure 2** and **3**.

From the secondary outcomes death was reported more frequently – in 33% (n=8) of the studies death was the reason for failure[17,27,30,31,35,36,44,46]. The secondary outcome resistance to antimicrobials, antivirals and antifungals was addressed in the introductions and discussions of the included studies, however not regarded as failure of therapy. Moreover, the presence and/or measurement of resistance to the study drug was described in 2 studies[27,29]. Boeckh et al report no resistance genes in the investigated patients and Palmer et al report one patient with known resistance to ganciclovir.

Not all identified cases of failure could be clarified even after contact with the authors of the studies. The reasons for stopping prophylaxis and pre-emptive therapy, *patient reasons* or *physician or sponsor decision* [27,29], were not explained in any of these studies. Moreover, in 2 studies[24,27] it was stated that prophylaxis was stopped because of *other* reasons. Adverse events, breakthrough infections and cause of death were mostly described in further detail in the included studies [15–17,27,29–31,35,42,44,46,48–50]. However, reasons 'other'[15,27], physician/investigator decision [17,27,29,49] and patients discretion [15] were grouped together in studies and not described in detail.

Risk of bias across studies

Risk of bias was assessed using 5 domains for RCTs: randomization, assignment and adherence to intervention, measurement of outcome, missing data, and selection of the reported results. For single-arm/observational studies domains were used: confounding, selection of participants, classification of interventions, missing data, measurement of outcomes and selection of the reported result. We concluded that all 16 RCTs and 6 single-arm had low risk of bias, 3 had some concerns and 2 had high risk of bias. Risk of bias in RCTs and single-arm studies is presented in **Tables 3** and **4**.

DISCUSSION

We aimed to explore the reasons why prophylaxis and pre-emptive therapy failed in transplant recipients. Twenty-four studies were included into this systematic review. We concluded that the main reason for stopping prophylaxis was adverse events for both CMV

For peer review only

and IFI prophylaxis and CMV pre-emptive therapy. We did not observe notable differences between the prophylaxis and pre-emptive therapy groups. This result was expected as cessation of ganciclovir therapy is often described due to debilitating side-effects, especially bone marrow suppression [15], which is even more problematic in hematological patients [28]. Different antifungal agents have a diverse safety profile. Our results were also in line with the common side-effects of antifungals as we observed nausea and vomiting in azoles and nephrotoxicity in Amphotericin B as reasons for discontinuation of therapy [43,46,48].

Adverse events are known to be underreported so the numbers that we summarized in this systematic review might be underestimated [52,53]. A review by Golder et al [53] looked into different study designs and reporting of adverse events. The authors outlined that there is wide underreporting of adverse events across different study designs and even more in the unpublished data. Unfortunately contacting the authors of the studies included in this systematic review did not result in additional information about the reported adverse events. Meta-analysis was not done as the studies were too heterogeneous.

Surprisingly, reporting of failure was insufficient in 25% of RCTs and 50% of prospective single-arm studies. One fourth of the studies did not report on preliminarily cessation of prophylaxis nor underlying reason. More worrisome was the fact that discontinuation of prophylaxis was mostly explained as adverse events and breakthrough infections, while patient and physicians' discretion as reasons were grouped together. On the other hand, we observed that only 5 studies did record infections that occurred after the prophylaxis had been stopped. This is concerning, as in a clinical setting and in developing guidelines it is important to make a distinction of breakthrough infections during and after prophylaxis and pre-emptive therapy [54]. Also, it makes it more difficult to compare the efficacy of different medications, for example when using ganciclovir and letermovir [54]. Furthermore, one of the included RCTs [17] reported early discharge from hospital as the main argument for stopping of prophylaxis. In addition, some reasons were not clarified, for example physician and patient discretion were combined in two of the included studies. A systematic review published in 2018 explored the efficacy and safety of CMV prophylaxis; adverse events and breakthrough infections were addressed, however the authors did not explicitly report additional information why patients stopped within these studies [55]. Similarly, in a PCP prophylaxis systematic review the authors described

For peer review only

adverse events as reason for discontinuation while not mentioning adherence, resistance or patients' choice [14].

A systematic review about quality of reporting RCTs in medical oncology described that 79% of the adverse events in studies are reported according to CONSORT criteria, although the description of participants and preliminary stopping in each stage of the study was done correctly in only 59% of the studies [56]. It has been argued that perhaps, poor reporting is deliberate to mask the shortcomings in study design [56,57]. One may expect that in a prospective study the patients are recruited and analyzed prospectively thus the data about failure of prophylaxis like adverse events should be readily obtainable. Thus, registering study protocols in clinical trial registers and following reporting guidelines helps consistent and more straightforward reporting of results [58,59]. Goldacre and colleagues have looked into reporting of clinical trials specifically with regards to outcomes. They concluded that most of the studies did not have sufficient reporting – either outcomes were added or prespecified outcomes were not reported. [60] These results raise concern to also whether failure is reported as per protocol. In our case, we faced a substantial quantity of missing data on failure and it is not always clear to us how studies were conducted with respect to exclusion of patients. An additional statement to the CONSORT criteria regarding better reporting of harms in randomized trials is a useful guidance document to improve conduct of studies and address shortcomings[61].

According to our search criteria we also planned to include studies looking into prophylaxis and pre-emptive therapy of PCP, HHV-6 virus and febrile neutropenia. Surprisingly, we were not able to identify suitable studies looking into prophylaxis of these infections for our systematic review. Current guidelines for the treatment of these infections use case reports, retrospective studies, surveillance studies, and outdated literature to give recommendations [5,62,63]. For instance, a systematic review focusing on PCP prophylaxis in non-HIV immunocompromised patients is used as main guidance of prophylaxis in this patient group, the studies included in this review date back from 1974 to 2008 (13 in the range 1977-1990) [14]. Moreover, the prophylaxis of febrile neutropenia is widely supported by a systematic review that included studies with quinolones published from 1980-2010 (10 in the range 1980-1997) [13]. Half of the studies in these reviews were published more than 20 years ago. Certainly, the evidence from these reviews are relevant to the field, however the landscape of treatment

For peer review only

of transplant recipients has changed – notably, with the emergence of resistant pathogens [64–67] and new data in todays' setting is needed to aid the update of clinical guidelines [63].

As mentioned before, detailed data about failure of prophylaxis and pre-emptive therapy was lacking in some studies and no additional information was obtained when reaching out to the authors. Furthermore, a limitation of our study was the restriction of start date (1st January 2010). This was done to avoid the effect of the significant change in management of infections and focus on the most recent evidence [11,12]. Another limitation is that we did not include studies about children. On the other hand, included studies already showed a heterogeneous variety of patient populations (e.g. autologous and allogeneic SCT recipients [19,48]), various types of infections and prophylaxis and pre-emptive therapy studies; therefore, we believe that adding paediatric studies would have further increased variability of our results.

There remains a variety of different practices between centers complicating patient transfers between hospitals [9,68]. We believe that having information about the discontinuation of failure of prophylaxis and pre-emptive therapy could provide valuable information for guideline committees, medical practitioners and researchers conducting studies with these medications. Without this information similar errors could be repeated in different studies.

There are several ways to predict failure in a clinical setting. Detecting adverse events and avoiding breakthrough infections can be done by approaching the patient or the treating physician. For example using mobile applications for reporting adverse events[69], defining the high-risk patients [70,71] and therapeutic drug monitoring of potentially toxic medications [72].

In **Figure 4** we have summarized guidance on how to detect failure early [69–77]. As mentioned above there are multiple studies that do not report the true breakthrough infections, only post-prophylaxis infections are reported. In addition, we believe that defining the potential adverse events before and describing the method of measurement of these could increase adherence. Other supporting background from the current systematic review for this figure is presented in **Appendix 5**.

Conclusions

In general, RCTs and prospective single-arm studies about prophylaxis and pre-emptive therapy of opportunistic infections should provide more in-depth information about failure.

For peer review only - http://bmjopen.bmj.com/site/about/guidelines.xhtml

The main reason why prophylaxis or pre-emptive therapy is stopped are adverse events, however these may well be underreported. Thus, the management and reporting of adverse events is critically important and should be improved in clinical studies. In addition, our results suggest partially biased approach in the publication of clinical studies and therefore there are insufficient data to support evidence-based decision-making in prophylaxis of PCP, HHV-6 and febrile neutropenia.

Competing interests

AGM was funded by Marie Skłodowska-Curie Actions, Grant Agreement number: 713660 — PRONKJEWAIL — H2020-MSCA-COFUND-2015.

Acknowledgements

The authors would like to thank Mariska M.G. Leeflang for her suggestions and help while analyzing and compiling the data of this systematic review.

Author statement

AG Märtson did the screening, writing, data analysis, risk of bias analysis, searches and planning, M Bakker did the screening, searches, data analysis, writing, H Blokzijl, EAM Verschuuren, SP, Berger, LFR Span, TS van der Werf did the planning, writing, reviewing of manuscript, JWC Alffenaar did the writing, analysis, planning.

Data availability statement

Unpublished data is available on request from the authors.

Patient and Public Involvement

This research was done without patient involvement. Patients were not invited to comment on the study design and were not consulted to develop patient relevant outcomes or interpret the results. Patients were not invited to contribute to the writing or editing of this document for readability or accuracy.

For peer review only

References

1. Ullmann AJ, Schmidt-Hieber M, Bertz H, et al. Infectious diseases in allogeneic haematopoietic stem cell transplantation: prevention and prophylaxis strategy guidelines 2016. *Ann Hematol*. 2016;95(9):1435-1455. doi:10.1007/s00277-016-2711-1

2. Menzin J, Meyers JL, Friedman M, et al. The economic costs to United States hospitals of invasive fungal infections in transplant patients. *Am J Infect Control*. 2011;39(4):e15-e20. doi:https://doi.org/10.1016/j.ajic.2010.06.009

3. Hakimi Z, Aballea S, Ferchichi S, et al. Burden of cytomegalovirus disease in solid organ transplant recipients: a national matched cohort study in an inpatient setting. *Transpl Infect Dis*. 2017;19(5). doi:10.1111/tid.12732

4. Iriart X, Bouar M Le, Kamar N, Berry A. Pneumocystis Pneumonia in Solid-Organ Transplant Recipients. *J fungi (Basel, Switzerland)*. 2015;1(3):293-331. doi:10.3390/jof1030293

5. Taplitz RA, Kennedy EB, Bow EJ, et al. Antimicrobial Prophylaxis for Adult Patients With Cancer-Related Immunosuppression: ASCO and IDSA Clinical Practice Guideline Update. *J Clin Oncol*. September 2018:JCO1800374. doi:10.1200/JCO.18.00374

6. Alsaeed M, Husain S. Infections in Heart and Lung Transplant Recipients. *Crit Care Clin*. 2019;35(1):75-93. doi:10.1016/j.ccc.2018.08.010

7. Freeman RBJ. The “indirect” effects of cytomegalovirus infection. *Am J Transplant*. 2009;9(11):2453-2458. doi:10.1111/j.1600-6143.2009.02824.x

8. Fishman JA. Infection in solid-organ transplant recipients. *N Engl J Med*. 2007;357(25):2601-2614. doi:10.1056/NEJMra064928

9. Manuel O, Kralidis G, Mueller NJ, et al. Impact of antiviral preventive strategies on the incidence and outcomes of cytomegalovirus disease in solid organ transplant recipients. *Am J Transplant*. 2013;13(9):2402-2410. doi:10.1111/ajt.12388

10. Navarro D, San-Juan R, Manuel O, et al. Cytomegalovirus infection management in

For peer review only

For peer review only - http://bmjopen.bmj.com/site/about/guidelines.xhtml

solid organ transplant recipients across European centers in the time of molecular diagnostics: An ESGICH survey. *Transpl Infect Dis*. 2017;19(6). doi:10.1111/tid.12773

11. Patterson TF, Thompson GR 3rd, Denning DW, et al. Practice Guidelines for the Diagnosis and Management of Aspergillosis: 2016 Update by the Infectious Diseases Society of America. *Clin Infect Dis*. 2016;63(4):e1-e60. doi:10.1093/cid/ciw326

12. Kotton CN, Kumar D, Caliendo AM, et al. The Third International Consensus Guidelines on the Management of Cytomegalovirus in Solid Organ Transplantation. *Transplantation*. March 2018. doi:10.1097/TP.0000000000002191

13. Gafter-Gvili A, Fraser A, Paul M, et al. Antibiotic prophylaxis for bacterial infections in afebrile neutropenic patients following chemotherapy. *Cochrane database Syst Rev*. 2012;1:CD004386. doi:10.1002/14651858.CD004386.pub3

14. Stern A, Green H, Paul M, Vidal L, Leibovici L. Prophylaxis for Pneumocystis pneumonia (PCP) in non-HIV immunocompromised patients. *Cochrane database Syst Rev*. 2014;(10):CD005590. doi:10.1002/14651858.CD005590.pub3

15. Humar A, Lebranchu Y, Vincenti F, et al. The efficacy and safety of 200 days valganciclovir cytomegalovirus prophylaxis in high-risk kidney transplant recipients. *Am J Transplant*. 2010;10(5):1228-1237. doi:10.1111/j.1600-6143.2010.03074.x

16. Liu, Amy W, Jutivorakool, Kamonwan, Fisher, Cynthia E., Rakita, Robert M., Reyes, Jorge D., Bhattacharya, Renuka B., Jerome, Keith R., Limaye, Ajit P. Comparison of preemptive therapy and antiviral prophylaxis for prevention of cytomegalovirus in seropositive liver transplant recipients. *Transplantation*. 2018;102(4):632-639. doi:10.1097/TP.0000000000002029 LK - http://rug.on.worldcat.org/atoztitles/link/?sid=EMBASE&issn=00411337&id=doi:10.1097%2FTP.0000000000002029&atitle=Comparison+of+preemptive+therapy+and+antiviral+prophylaxis+for+prevention+of+cytomegalovirus+in+seropositive+liver+transplant+recipients&stitle=Transplantation&title=Transplantation&volume=102&issue=4&spage=632&epage=639&aulast=Liu&aufirst=Amy+W.&auinit=A.W.&aufull=Liu+A.W.&coden=TRPLA&isbn=&pages=632-639&date=2018&auinit1=A&auinitm=W.

17. Winston DJ, Limaye AP, Pelletier S, et al. Randomized, double-blind trial of

For peer review only - http://bmjopen.bmj.com/site/about/guidelines.xhtml

anidulafungin versus fluconazole for prophylaxis of invasive fungal infections in high-risk liver transplant recipients. *Am J*. 2014;14(12):2758-2764. doi:10.1111/ajt.12963

18. van der Beek MT, Berger SP, Vossen ACTM, et al. Preemptive versus sequential prophylactic-preemptive treatment regimens for cytomegalovirus in renal transplantation: comparison of treatment failure and antiviral resistance. *Transplantation*. 2010;89(3):320-326. doi:10.1097/TP.0b013e3181bc0301

19. Huang X, Chen H, Han M, et al. Multicenter, Randomized, Open-Label Study Comparing the Efficacy and Safety of Micafungin versus Itraconazole for Prophylaxis of Invasive Fungal Infections in Patients undergoing Hematopoietic Stem Cell Transplant. *Biol Blood Marrow Transplant*. 2012;18(10):1509-1516. doi:10.1016/j.bbmt.2012.03.014

20. Helfrich M, Dorschner P, Thomas K, Stosor V, Ison MG. A retrospective study to describe the epidemiology and outcomes of opportunistic infections after abdominal organ transplantation. *Transpl Infect Dis*. 2017;19(3):e12691. doi:10.1111/tid.12691

21. Liberati A, Altman DG, Tetzlaff J, et al. The PRISMA Statement for Reporting Systematic Reviews and Meta-Analyses of Studies That Evaluate Health Care Interventions: Explanation and ElaborationPRISMA: Explanation and Elaboration. *Ann Intern Med*. 2009;151(4):W-65-W-94. doi:10.7326/0003-4819-151-4-200908180-00136

22. Higgins JPT, Sterne JAC, Savović J, Page MJ, Hróbjartsson A, Boutron I, Reeves B ES. A revised tool for assessing risk of bias in randomized trials In: Chandler J, McKenzie J, Boutron I, Welch V (editors). Cochrane Methods. *Cochrane Database Syst Rev*. 2016;(10 (Suppl 1)).

23. Sterne JAC, Hernán MA, Reeves BC, et al. ROBINS-I: a tool for assessing risk of bias in non-randomised studies of interventions. *BMJ*. 2016;355:i4919. doi:10.1136/bmj.i4919

24. Humar A, Limaye AP, Blumberg EA, et al. Extended valganciclovir prophylaxis in D+/R- kidney transplant recipients is associated with long-term reduction in cytomegalovirus disease: two-year results of the IMPACT study. *Transplantation*. 2010;90(12):1427-

For peer review only - http://bmjopen.bmj.com/site/about/guidelines.xhtml

1431.

25. Padulles A, Colom H, Bestard O, et al. Contribution of population pharmacokinetics to dose optimization of ganciclovir-valganciclovir in solid-organ transplant patients. *Antimicrob Agents Chemother*. 2016;60(4):1992-2002. doi:10.1128/AAC.02130-15

26. Finlen CC, Davis W, Snyder L, et al. Long-term efficacy and safety of 12 months of valganciclovir prophylaxis compared with 3 months after lung transplantation: A single-center, long-term follow-up analysis from a randomized, controlled cytomegalovirus prevention trial. *J Hear lung Transplant*. 2011;30(9):990-996. doi:10.1016/j.healun.2011.02.017

27. Palmer S, Limaye A, Banks M, et al. Extended valganciclovir prophylaxis to prevent cytomegalovirus after lung transplantation: a randomized, controlled trial. *Ann Intern Med*. 2010;152(12):761-769. doi:10.7326/0003-4819-152-12-201006150-00003

28. Chawla J, Ghobadi A, Mosley J, et al. Oral valganciclovir versus ganciclovir as delayed pre-emptive therapy for patients after allogeneic hematopoietic stem cell transplant: a pilot trial (04-0274) and review of the literature. *Transpl Infect Dis*. 2012;14(3):259-267. doi:10.1111/j.1399-3062.2011.00689.x

29. Boeckh M, Nichols W, Chemaly R, et al. Valganciclovir for the prevention of complications of late cytomegalovirus infection after allogeneic hematopoietic cell transplantation: A randomized trial. *Ann Intern Med*. 2015;162(1):1-10. doi:10.7326/M13-2729

30. Kim S, Lee M, Kim S, et al. A randomized trial of preemptive therapy for prevention of cytomegalovirus disease after allogeneic hematopoietic stem cell transplantation. *Int J Hematol*. 2010;91(5):886-891. doi:10.1007/s12185-010-0580-2

31. Halim M, Al-Otaibi T, Gheith O, et al. Efficacy and safety of low-dose versus standard-dose valganciclovir for prevention of cytomegalovirus disease in intermediate-risk kidney transplant recipients. *Exp Clin Transplant*. 2016;14(5):526-534. doi:10.6002/ect.2015.0305

32. Gheith O, Halim MA, Al-Otaibi T, et al. Successful cost-effective prevention of

For peer review only

For peer review only - http://bmjopen.bmj.com/site/about/guidelines.xhtml

cytomegalovirus disease in kidney transplant recipients using low-dose valganciclovir. *Exp Clin Transplant*. 2017;15:156-163. doi:10.6002/ect.mesot2016.P34

33. Witzke O, Hauser I, Bartels M, Wolf G, Wolters H, Nitschke M. Valganciclovir Prophylaxis Versus Preemptive Therapy in Cytomegalovirus-Positive Renal Allograft Recipients: 1-Year Results of a Randomized Clinical Trial. *Transplantation*. 2012;93(1):61-68. doi:10.1097/TP.0b013e318238dab3

34. Witzke O, Nitschke M, Bartels M, et al. Valganciclovir Prophylaxis Versus Preemptive Therapy in Cytomegalovirus-Positive Renal Allograft Recipients: long-term Results after 7 Years of a Randomized Clinical Trial. *Transplantation*. 2018;102(5):876-882. doi:10.1097/TP.0000000000002024

35. Reischig T, Hribova P, Jindra P, et al. Long-term outcomes of pre-emptive valganciclovir compared with valacyclovir prophylaxis for prevention of cytomegalovirus in renal transplantation. *J Am Soc Nephrol*. 2012;23(9):1588-1597. doi:10.1681/ASN.2012010100

36. Reischig T, Kacer M, Jindra P, Hes O, Lysak D, Bouda M. Randomized trial of valganciclovir versus valacyclovir prophylaxis for prevention of cytomegalovirus in renal transplantation. *Clin J Am Soc Nephrol*. 2015;10(2):294-304. doi:10.2215/CJN.07020714

37. Reischig T, Prucha M, Sedlackova L, et al. Valganciclovir prophylaxis against cytomegalovirus impairs lymphocyte proliferation and activation in renal transplant recipients. *Antivir Ther*. 2011;16(8):1227-1235. doi:10.3851/IMP1879

38. Togashi J, Sugawara Y, Hashimoto M, et al. Oral valganciclovir versus intravenous ganciclovir as preemptive treatment for cytomegalovirus infection after living donor liver transplantation: a randomized trial. *Biosci Trends*. 2011;5(5):217-222. http://onlinelibrary.wiley.com/o/cochrane/clcentral/articles/974/CN-00881974/frame.html.

39. Montejo, M, Porto, M, Carmon, O, Zárraga, S, Gainza, J, Amenabar, JJ, Goikoetxea, J, Bereciartua E LI. Prophylactic therapy with valganciclovir in high-risk (cytomegalovirus D+/R-) kidney transplant recipients: A single-center experience. *Proceedings*.

For peer review only

2010;42(8):2947-2949. doi:10.1016/j.transproceed.2010.08.017

40. Nanmoku, K, Shinzato, T, Kubo, T, Shimizu, T, Kimura, T, Yagisawa T. Prevention of Late-Onset Cytomegalovirus Infection and Disease in Donor-Positive/Recipient-Negative Kidney Transplant Recipients Using Low-Dose Valganciclovir. *Transplant Proc*. 2018;50(1):124-129. doi:10.1016/j.transproceed.2017.12.025 LK - http://rug.on.worldcat.org/atoztitles/link/?sid=EMBASE&issn=18732623&id=doi:10.1016%2Fj.transproceed.2017.12.025&atitle=Prevention+of+Late-Onset+Cytomegalovirus+Infection+and+Disease+in+Donor-Positive%2FRecipient-Negative+Kidney+Transplant+Recipients+Using+Low-Dose+Valganciclovir&stitle=Transplant.+Proc.&title=Transplantation+Proceedings&volume=50&issue=1&spage=124&epage=129&aulast=Nanmoku&aufirst=K.&auinit=K.&aufull=Nanmoku+K.&coden=TRPPA&isbn=&pages=124

41. Perrottet N, Csajka C, Pascual M, et al. Population Pharmacokinetics of Ganciclovir in Solid-Organ Transplant Recipients Receiving Oral Valganciclovir. *Antimicrob Agents Chemother*. 2009;53(7):3017 LP - 3023. doi:10.1128/AAC.00836-08

42. McGillicuddy JW, Weimert NA, Taber DJ, et al. Can preemptive cytomegalovirus monitoring be as effective as universal prophylaxis when implemented as the standard of care in patients at moderate risk? *Transplantation*. 2010;89(10):1218-1223. doi:10.1097/TP.0b013e3181d54ba6

43. Mitsani D, Nguyen MH, Shields RK, et al. A prospective, observational study of voriconazole therapeutic drug monitoring among lung transplant recipients receiving prophylaxis: Factors impacting levels and associations between serum troughs, efficacy and toxicity. *Antimicrob Agents Chemother*. 2012;56. doi:10.1128/AAC.05219-11

44. Cordonnier C, Rovira M, Maertens J, et al. Voriconazole for secondary prophylaxis of invasive fungal infections in allogeneic stem cell transplant recipients: results of the VOSIFI study. *Haematologica*. 2010;95(10):1762-1768. doi:10.3324/haematol.2009.020073

45. Takenaka, K, Nagafuji, K, Takase, K, Kamimura, T, Mori, Y, Ito, Y, Nishi, Y, Henzan, H,

For peer review only

For peer review only - http://bmjopen.bmj.com/site/about/guidelines.xhtml

Kato, K, Harada, N, Eto, T, Miyamoto, T, Teshima, T, Akashi K. Initial low-dose valganciclovir as a preemptive therapy is effective for cytomegalovirus infection in allogeneic hematopoietic stem cell transplant recipients. *Int J Hematol*. 2012;96(1):94-100. doi:10.1007/s12185-012-1087-9

46. Park S-Y, Lee S-O, Choi S-H, et al. Efficacy and safety of low-dose ganciclovir preemptive therapy in allogeneic haematopoietic stem cell transplant recipients compared with conventional-dose ganciclovir: a prospective observational study. *J Antimicrob Chemother*. 2012;67(6):1486-1492. doi:10.1093/jac/dks043

47. Perrella A, Esposito C, Pisaniello D, et al. Role of liposomal amphotericin B prophylaxis after liver transplantation compared with fluconazole for high-risk patients. impact on infections and mortality within one year. *Transplant Proc*. 2012;44(7):1977-1981. doi:10.1016/j.transproceed.2012.06.013

48. Chaftari A-M, Hachem RY, Ramos E, et al. Comparison of posaconazole versus weekly amphotericin B lipid complex for the prevention of invasive fungal infections in hematopoietic stem-cell transplantation. *Transplantation*. 2012;94(3):302-308. doi:10.1097/TP.0b013e3182577485

49. Park S, Kim K, Jang JH, et al. Randomized trial of micafungin versus fluconazole as prophylaxis against invasive fungal infections in hematopoietic stem cell transplant recipients. *J Infect*. 2016;73(5):496-505. doi:10.1016/j.jinf.2016.06.011

50. Mitsani D, Nguyen MH, Shields RK, et al. Prospective, observational study of voriconazole therapeutic drug monitoring among lung transplant recipients receiving prophylaxis: Factors impacting levels of and associations between serum troughs, efficacy, and toxicity. *Antimicrob Agents Chemother*. 2012;56(5):2371-2377. doi:10.1128/AAC.05219-11

51. Manuel O, Kralidis G, Mueller NJ, et al. Impact of antiviral preventive strategies on the incidence and outcomes of cytomegalovirus disease in solid organ transplant recipients. *Am J Transplant*. 2013;13(9):2402-2410. doi:10.1111/ajt.12388

52. Hazell L, Shakir SAW. Under-Reporting of Adverse Drug Reactions. *Drug Saf*. 2006;29(5):385-396. doi:10.2165/00002018-200629050-00003

For peer review only

For peer review only - http://bmjopen.bmj.com/site/about/guidelines.xhtml

53. Golder S, Loke YK, Wright K, Norman G. Reporting of Adverse Events in Published and Unpublished Studies of Health Care Interventions: A Systematic Review. *PLoS Med*. 2016;13(9):e1002127-e1002127. doi:10.1371/journal.pmed.1002127

54. Chen K, Cheng MP, Hammond SP, Einsele H, Marty FM. Antiviral prophylaxis for cytomegalovirus infection in allogeneic hematopoietic cell transplantation. *Blood Adv*. 2018;2(16):2159-2175. doi:10.1182/bloodadvances.2018016493

55. Gagelmann N, Ljungman P, Styczynski J, Kroger N. Comparative Efficacy and Safety of Different Antiviral Agents for Cytomegalovirus Prophylaxis in Allogeneic Hematopoietic Cell Transplantation: A Systematic Review and Meta-Analysis. *Biol Blood Marrow Transplant*. May 2018. doi:10.1016/j.bbmt.2018.05.017

56. You B, Maillet D, Chen EX, et al. Quality of Reporting of Modern Randomized Controlled Trials in Medical Oncology: A Systematic Review. *JNCI J Natl Cancer Inst*. 2012;104(13):982-989. doi:10.1093/jnci/djs259

57. Huwiler-Muntener K, Juni P, Junker C, Egger M. Quality of reporting of randomized trials as a measure of methodologic quality. *JAMA*. 2002;287(21):2801-2804.

58. Alley AB, Seo J-W, Hong S-T. Reporting results of research involving human subjects: an ethical obligation. *J Korean Med Sci*. 2015;30(6):673-675. doi:10.3346/jkms.2015.30.6.673

59. Schulz KF, Altman DG, Moher D. CONSORT 2010 Statement: updated guidelines for reporting parallel group randomised trials. *BMJ*. 2010;340:c332. doi:10.1136/bmj.c332

60. Ben Goldacre, Henry Drysdale, Anna Powell-Smith, Aaron Dale, Ioan Milosevic, Eirion Slade, Philip Hartley, Cicely Marston, Kamal Mahtani CH. The COMPare Trials Project.

61. Ioannidis JPA, Evans SJW, Gøtzsche PC, et al. Better reporting of harms in randomized trials: an extension of the CONSORT statement. *Ann Intern Med*. 2004;141(10):781-788. doi:10.7326/0003-4819-141-10-200411160-00009

62. Betts BC, Young J-AH, Ustun C, Cao Q, Weisdorf DJ. Human Herpesvirus 6 Infection after Hematopoietic Cell Transplantation: Is Routine Surveillance Necessary? *Biol*

For peer review only

Blood Marrow Transplant. 2011;17(10):1562-1568. doi:10.1016/j.bbmt.2011.04.004

63. Cooley L, Dendle C, Wolf J, et al. Consensus guidelines for diagnosis, prophylaxis and management of Pneumocystis jirovecii pneumonia in patients with haematological and solid malignancies, 2014. *Intern Med J*. 2014;44(12b):1350-1363. doi:10.1111/imj.12599

64. Ivulich S, Westall G, Dooley M, Snell G. The Evolution of Lung Transplant Immunosuppression. *Drugs*. 2018;78(10):965-982. doi:10.1007/s40265-018-0930-6

65. Krisl JC, Doan VP. Chemotherapy and Transplantation: The Role of Immunosuppression in Malignancy and a Review of Antineoplastic Agents in Solid Organ Transplant Recipients. *Am J Transplant*. 2017;17(8):1974-1991. doi:10.1111/ajt.14238

66. Soderlund C, Radegran G. Immunosuppressive therapies after heart transplantation--The balance between under- and over-immunosuppression. *Transplant Rev (Orlando)*. 2015;29(3):181-189. doi:10.1016/j.trre.2015.02.005

67. Baron D, Giral M, Brouard S. Reconsidering the detection of tolerance to individualize immunosuppression minimization and to improve long-term kidney graft outcomes. *Transpl Int*. 2015;28(8):938-959. doi:10.1111/tri.12578

68. Hand J. Strategies for Antimicrobial Stewardship in Solid Organ Transplant Recipients. *Infect Dis Clin North Am*. 2018;32(3):535-550. doi:10.1016/j.idc.2018.04.003

69. de Vries ST, Denig P, Lasheras Ruiz C, et al. Interest in a Mobile App for Two-Way Risk Communication: A Survey Study Among European Healthcare Professionals and Patients. *Drug Saf*. 2018;41(7):697-712. doi:10.1007/s40264-018-0648-0

70. Vallejo C, Vazquez L, Cabrera Martin JR, et al. Treatment of invasive fungal infections in high-risk haematological patients: what have we learnt in the past 10 years? *Rev Esp Quimioter*. 2013;26(4):378-386.

71. Freeman RB, Cohen JT. Transplantation Risks and the Real World: What Does ‘High Risk’ Really Mean? *Am J Transplant*. 2009;9(1):23-30. doi:10.1111/j.1600-6143.2008.02476.x

For peer review only

608 72. Roberts JA, Abdul-Aziz MH, Lipman J, et al. Individualised antibiotic dosing for patients who are critically ill: challenges and potential solutions. *Lancet Infect Dis*. 2014;14(6):498-509. doi:https://doi.org/10.1016/S1473-3099(14)70036-2

611 73. Larizgoitia I, Bouesseau M-C, Kelley E. WHO Efforts to Promote Reporting of Adverse Events and Global Learning. *J Public health Res*. 2013;2(3):e29-e29. doi:10.4081/jphr.2013.e29

614 74. Razonable RR, Hayden RT. Clinical utility of viral load in management of cytomegalovirus infection after solid organ transplantation. *Clin Microbiol Rev*. 2013;26(4):703-727. doi:10.1128/CMR.00015-13

617 75. Boch T, Spiess B, Cornely O, et al. Diagnosis of invasive fungal infections in haematological patients by combined use of galactomannan, 1,3-beta-D-glucan, Aspergillus PCR, multifungal DNA-microarray, and Aspergillus azole resistance PCRs in blood and bronchoalveolar lavage samples: results. *Clin Microbiol Infect*. 2016;22(10):862-868. doi:10.1016/j.cmi.2016.06.021

622 76. Patzer RE, Serper M, Reese PP, et al. Medication understanding, non-adherence, and clinical outcomes among adult kidney transplant recipients. *Clin Transplant*. 2016;30(10):1294-1305. doi:10.1111/ctr.12821

625 77. Zanetti-Yabur A, Rizzo A, Hayde N, Watkins AC, Rocca JP, Graham JA. Exploring the usage of a mobile phone application in transplanted patients to encourage medication compliance and education. *Am J Surg*. 2017;214(4):743-747. doi:https://doi.org/10.1016/j.amjsurg.2017.01.026

For peer review only

Figure 1. PRISMA diagram

Figure 2. Failure of CMV prophylaxis and pre-emptive therapy in 11 RCTs (y-axis represents the proportion of the transplant group in percentages and x-axis represents transplant).

Figure 3. Failure of IFI prophylaxis in 5 RCTs (y-axis represents the proportion of the transplant group in percentages and x-axis represents transplant). For one study, 15% of failure was caused by patients discharged early, this has not been included in this figure.

Figure 4. Detecting failure in a clinical setting.

For peer review only

Table 1. Study characteristics

Author, year	Country	Study design	Population	Preemptive therapy/ prophylaxis	Prophylaxis/preemptive medication	Comparison	Sample size	Duration	Follow-up	Summary assessment of risk of bias
**CYTOMEGALVIRUS PROPHYLAXIS/PREEMPTIVE THERAPY**										
**Humar et al, 2010[1], 2010[2]**	Australia, Belgium, Canada, Brazil, France, Germany, the UK, Italy, Poland, Romania, Spain, USA	RCT	Kidney transplant recipients aged 17-77 years	Prophylaxis	Valganciclovir	Valganciclovir different duration and placebo	320 patients	100 and 200 days	12 months	Low risk
**Halim et al, 2016**<br>**Gheith et al, 2017**	Kuwait, Egypt	RCT	Kidney transplant recipients with mean age of 42.4 years	Prophylaxis	Valganciclovir	Valganciclovir different duration	201 patients	98.1 days (mean)	12 months	Some concerns
**Witzke et al 2012, 2018**	Germany, Austria	RCT	Kidney transplant recipients with	Prophylaxis and pre-	Valganciclovir	Valganciclovir as	296 patients	100 days (14 days preemptive	7 years	Some concerns

For peer review only - http://bmjopen.bmj.com/site/about/guidelines.xhtml

			mean age of 52.7 years	emptive therapy		preemptive treatment		until 100 days)		
**Reischig et al 2012**	Czeck Republic	RCT	Kidney transplant recipients with mean age of 49 years	Prophylaxis and pre-emptive therapy	Valganciclovir	Valacyclovir	70 patients	3 months	2 years	Some concerns
**Reischig et al 2011, 2015**	Czeck Republic	RCT	Kidney transplant recipients with mean age of 49 years	Prophylaxis	Valganciclovir	Valacyclovir	119 patients	3 months	1 year	Some concerns
**Togashi et al 2011**	Japan	RCT	Liver transplant recipients with mean age of 52.1 years	Preemptive therapy	Valganciclovir	Ganciclovir	22 patients	2 weeks	1 year	Some concerns
**Padulles et al 2016**	Spain	RCT	Kidney, liver and heart transplant recipients with mean age of 53.9 years	Prophylaxis and treatment	Valganciclovir/ Ganciclovir	Valganciclovir /Ganciclovir different dosing	53 patients	NA (not reported)	6 months	Low risk
**Palmer et al 2010**	USA	RCT	Lung transplant recipients with	Prophylaxis	Valganciclovir	Placebo	136 patients	12 months	3.9 years (mean)	Some concerns

For peer review only - http://bmjopen.bmj.com/site/about/guidelines.xhtml

**Finlen Copeland et al 2011**			median age of 55.5 years							
**Chawla et al 2011**	USA	RCT	Allogeneic transplant recipients aged 18-64	Preemptive therapy	Valganciclovir	Ganciclovir	37 patients	28 days	4 weeks	High risk
**Boeckh et al 2015**	USA	RCT	Allogeneic transplant recipients aged 16-70	Preemptive therapy	Valganciclovir	Placebo	184 patients	150/120 days valganciclovir/placebo (medians)	640 days	Some concerns
**Kim et al 2010**	South Korea	RCT	Allogeneic transplant recipients aged 16-49	Preemptive therapy	Ganciclovir	Ganciclovir different dosing	68 patients	14 days (median)	42 months (median)	Some concerns
**Montejo et al 2010**	Spain	Single-arm	Kidney transplant recipients with median age 47 years	Prophylaxis	Valganciclovir	NA	34 patients	3 months	1 year	Critical risk
**Nanmoku et al 2018**	Japan	Single-arm	Kidney transplant recipients with	Prophylaxis	Valganciclovir	NA	100 patients	6 months	1 year	Serious risk

For peer review only - http://bmjopen.bmj.com/site/about/guidelines.xhtml

			mean age 48.7 years							
**Perrottet et al 2009**<br>**Manuel et al 2010**	France	Single-arm	Kidney , heart, lung, liver transplant recipients aged 18-70	Prophylaxis	Valganciclovir and Ganciclovir	NA	65 patients	3 months	1 year	Serious risk
**McGillicudy et al 2010**	USA	Single-arm	Kidney and pancreas transplant recipients mean age 53.5 years	Preemptive therapy and prophylaxis	Valganciclovir/ ganciclovir	Valganciclovir /ganciclovir preemptive therapy	130 patients	90 days	1 year	Serious risk
**Takenaka et al 2012**	Japan	Single-arm	Allogeneic transplant recipients aged 25-68	Preemptive therapy	Valganciclovir	NA	20 patients	2 weeks	10 weeks	Serious risk
**Park et al 2012**	South Korea	Single-arm	Allogeneic transplant recipients aged 16-70	Preemptive therapy	Ganciclovir	Ganciclovir different dosing	97 patients	14 days (median)	Not same for all patients	Moderate risk
**INVASIVE FUNGAL INFECTION PROPHYLAXIS**										
**Perrella et al 2012**	Italy	RCT	Liver transplant	Prophylaxis	Fluconazole	Amphotericin B	43 patients	7-14 days	1 year	Some concerns

For peer review only - http://bmjopen.bmj.com/site/about/guidelines.xhtml

			recipients (age NI)							
**Winston et al 2014**	USA	RCT	Liver transplant recipients aged 19-75 years	Prophylaxis	Fluconazole	Anidulafungin	200 patients	21 days (median)	90 days	Low risk
**Chaftari et al 2012**	USA	RCT	Allogeneic transplant recipients aged 20-69	Prophylaxis	Posaconazole	Amphotericin B lipid complex	40 patients	6 weeks	2 weeks	Some concerns
**Huang et al 2012**	China	RCT	Allogeneic and autologous transplant recipients with mean age 32.7 years	Prophylaxis	Itraconazole	Micafungin	228 patients	42 days	4 weeks	Some concerns
**Park et al 2016**	South Korea	RCT	Allogeneic and autologous transplant recipients aged 20-64	Prophylaxis	Fluconazole	Micafungin	250 patients	21 days	100 days	Some concerns
**Mitsani et al 2012**	USA	Single-arm	Lung transplant recipients	Prophylaxis	Voriconazole	NA	93 patients	3 months	NI	Moderate risk

For peer review only - http://bmjopen.bmj.com/site/about/guidelines.xhtml

			aged 20-74 years							
**Cordonnier et al 2010**	France, Spain, Belgium, UK, Germany, Switzerland, Portugal, Sweden	Single-arm	Allogeneic transplant recipients aged 22-72 years	Prophylaxis	Voriconazole	NA	45 patients	94 days (median)	1 year	Critical risk

RCT – randomized controlled trial, NA – not applicable, NI – no information

Summary assessment of risk of bias grades for:

RCTs using RoB tool:

Low risk of bias: The study is judged to be at low risk of bias for all domains for this result.

Some concerns: The study is judged to raise some concerns in at least one domain for this result, but not to be at high risk of bias for any domain.

High risk of bias: The study is judged to be at high risk of bias in at least one domain for this result.

Single-arm prospective studies using ROBINS tool:

Low risk of bias: = The study is judged to be at low risk of bias for all domains.

Moderate risk of bias: The study is judged to be at low or moderate risk of bias for all domains.

Serious risk of bias: The study is judged to be at serious risk of bias in at least one domain, but not at critical risk of bias in any domain.

Critical risk of bias: The study is judged to be at critical risk of bias in at least one domain.

For peer review only - http://bmjopen.bmj.com/site/about/guidelines.xhtml

For peer review only

For peer review only - http://bmjopen.bmj.com/site/about/guidelines.xhtml

Table 2. Reasons for stopping prophylaxis or pre-emptive therapy

Cytomegalovirus prophylaxis/pre-emptive therapy

**Study**	AE	Breakthrough infection	Graft loss	Death	Patient decision	Physician/ investigator decision	Other	Total number subjects
**Humar et al, 2010**	27 (8%)	51 (16%)			11 (3%)		14 (4%)	320
**Halim et al, 2016**			2 (1%)	1 (0.5%)				201
**Reischig et al 2012**			10 (14%)	2 (3%)				70
**Reischig et al 2015[1]**	13 (11%)		4 (3%)	3 (2.5%)				119
**Padulles et al 2016**	1 (2%)							53
**Palmer et al 2010**	17 (12.5%)			7 (5%)	3 (2%)	15 (11%)	3 (2%)	136
**Boeckh et al 2015**	44 (24%)	35 (19%)				20 (11%)		184
**Kim et al 2010**	1 (1.5%)			2 (3%)				68
**Montejo et al 2010**		3 (9%)						34
**Perrottet et al 2009**		3 (viremia)						65

For peer review only

For peer review only - http://bmjopen.bmj.com/site/about/guidelines.xhtml

**McGillicudy et al 2010**	3 (2%)	1 (1%) [2]			130
**Park et al 2012 [3]**	8 (8%)	6 (6%)	37 (38%)		97
**Invasive fungal infection prophylaxis**					
**Winston et al 2014 [4]**<br>**Fluconazole**		2 (2%)	1 (1%)	2 (2%)	100 (fluconazole)<br>200 (total)
**Chaftari et al 2012**<br>**Posaconazole**	8 (38%)				21 (posaconazole)<br>40 (total)
**Huang et al 2012**<br>**Itraconazole**	29 (20%)	12 (8%)	1 (1%)	4 (3%)	147 (itraconazole)<br>228 (total)
**Park et al 2016**<br>**Fluconazole**	2 (2%)	3 (3%)		3 (3%)	89 (fluconazole)<br>250 (total)
**Mitsani et al 2012**	25 (27%)				93
**Cordonnier et al 2010**	2 (4%)	3* (7%)	11* (2%)		45
**NI in the following trials (timepoints not specified)**					
Nanmoku et al 2018					100
Chawla et al 2011					37
Witzke et al 2012					296

For peer review only - http://bmjopen.bmj.com/site/about/guidelines.xhtml

**Perrella et al 2012**	43
**All patients completed treatment protocol in the following trials**	
**Togashi et al 2011**	22
**Takenaka et al 2012**	20

AE – adverse event, NI – no information (not reported), IFI – invasive fungal infection, CMV – cytomegalovirus

* time-point not confirmed

1. 1 patient moved to another country

2. No information on breakthrough infections during prophylaxis

3. 12 patients had dosage reductions

4. 36 patients were preliminarily stopped due to discharge from hospital

For peer review only

Table 3. Risk of bias in RCTs using RoB tool

	Randomization process	Assignment of intervention	Adhering to intervention	Measurement of outcomes	Missing outcome data	Selection of the reported result
Humar et al 2010	+	+	+	+	+	?
Halim et al 2016	?	+	+	?	+	?
Witzke et al 2012	+	+	+	?	+	?
Reischig et al 2012	+	+	+	?	+	?
Reischig et al 2015	+	+	+	?	+	?
Togashi et al 2011	?	+	+	?	+	?
Padulles et al 2016	+	+	+	+	+	?
Palmer et al 2010	+	+	?	+	+	?
Chawla et al 2011	?	?	?	?	+	?
Boeckh et al 2015	+	+	?	+	+	?
Kim et al 2010	?	+	+	?	+	?
Perrella et al 2012	?	?	?	?	+	?
Winston et al 2014	+	+	+	+	+	?
Chaftari et al 2012	+	?	?	?	+	?
Huang et al 2012	+	+	?	?	+	?
Park et al 2016	?	+	+	?	+	?

+ low risk, - high risk, ? some concerns

For peer review only

Table 4. Risk of bias in single-arm studies using ROBINs tool

	Confounding	Selection of participants	Classification of interventions	Deviations from intended interventions	Missing data	Measurement of outcomes	Selection of the reported result
Montejo et al 2010	-	?	+	NI	-	?	NI
Nanmoku et al 2018	*	+	+	+	+	?	NI
Perrottett et al 2009	*	?	+	?	*	?	NI
McGillicuddy et al 2010	*	+	+	*	*	?	NI
Takenaka et al 2012	*	?	+	?	*	?	NI
Park et al 2012	?	+	+	?	+	?	NI
Mitsani et al 2012	*	?	+	?	?	?	NI
Cordonnier et al 2010	*	*	+	?	?	?	NI

+ low risk,? moderate risk, *serious risk, - critical risk

NI = no information

For peer review only

For peer review only - http://bmjopen.bmj.com/site/about/guidelines.xhtml

For peer review only

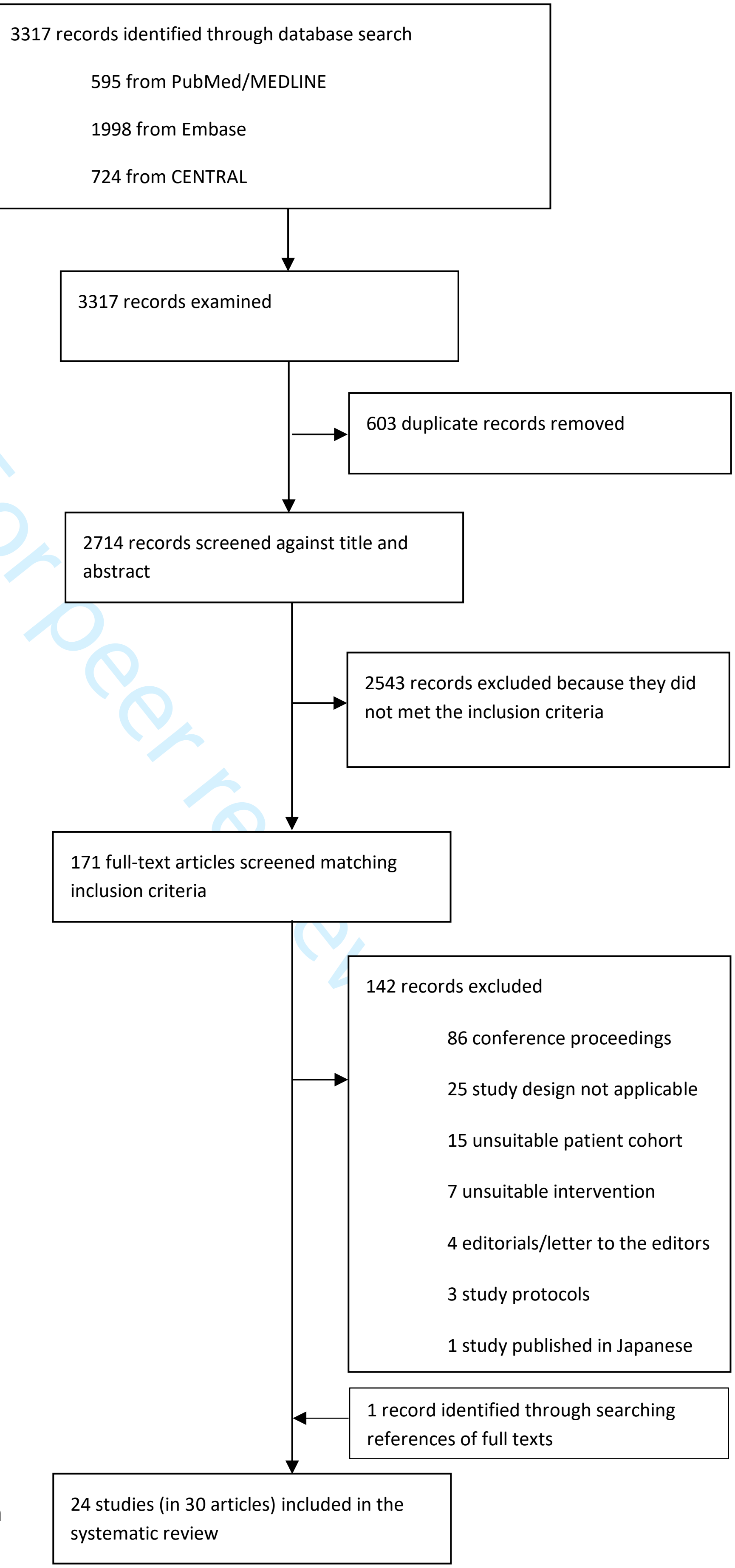

Figure 1. PRISMA diagram

For peer review only - http://bmjopen.bmj.com/site/about/guidelines.xhtml

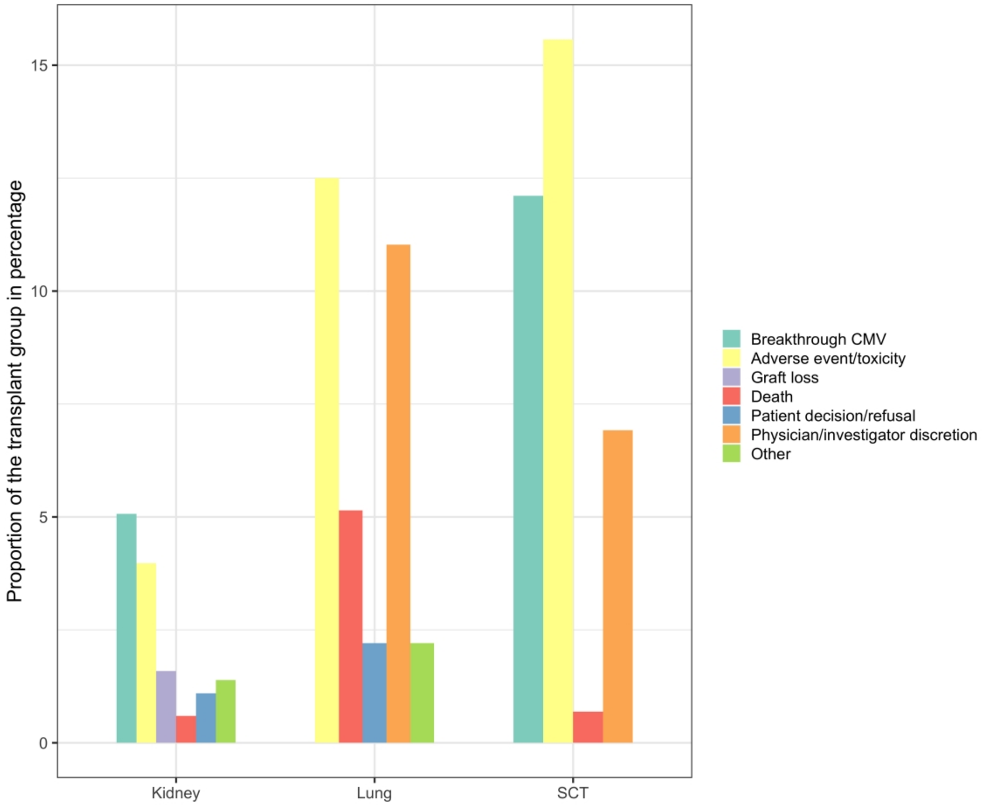

Figure 2. Failure of CMV prophylaxis and pre-emptive therapy in 11 RCTs (y-axis represents the proportion of the transplant group in percentages and x-axis represents transplant).

1328x1116mm (72 x 72 DPI)

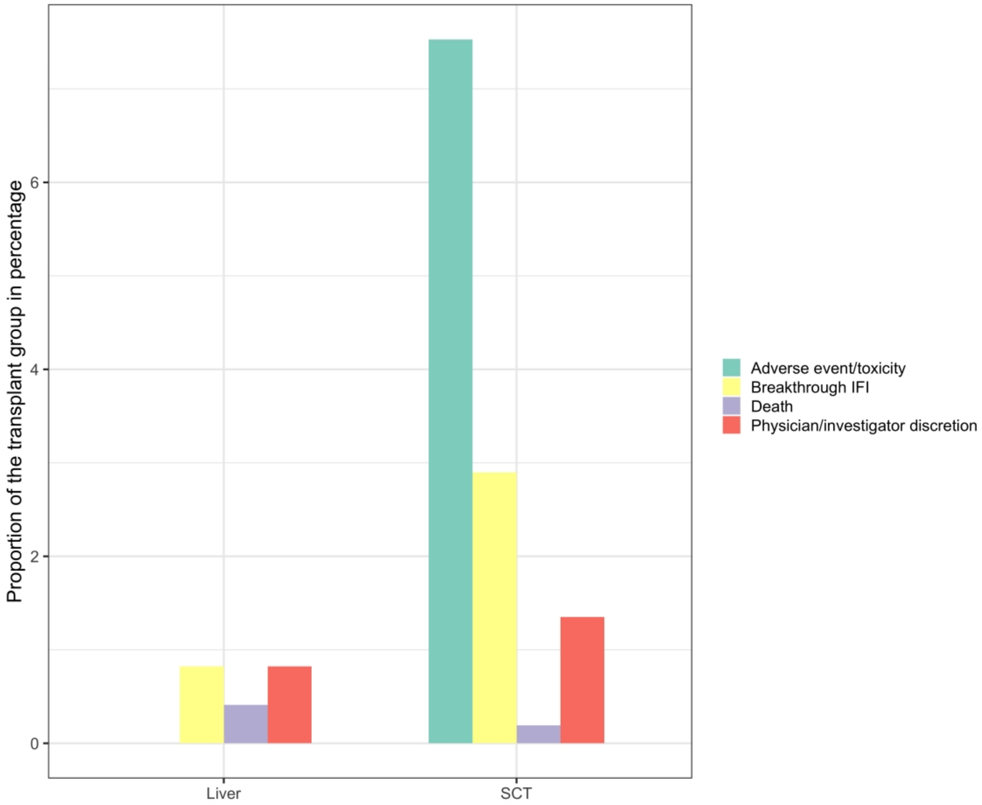

Figure 3. Failure of IFI prophylaxis in 5 RCTs (y-axis represents the proportion of the transplant group in percentages and x-axis represents transplant). For one study, 15% of failure was caused by patients discharged early, this has not been included in this figure.

1328x1116mm (72 x 72 DPI)

Figure 4. Detecting failure in a clinical setting

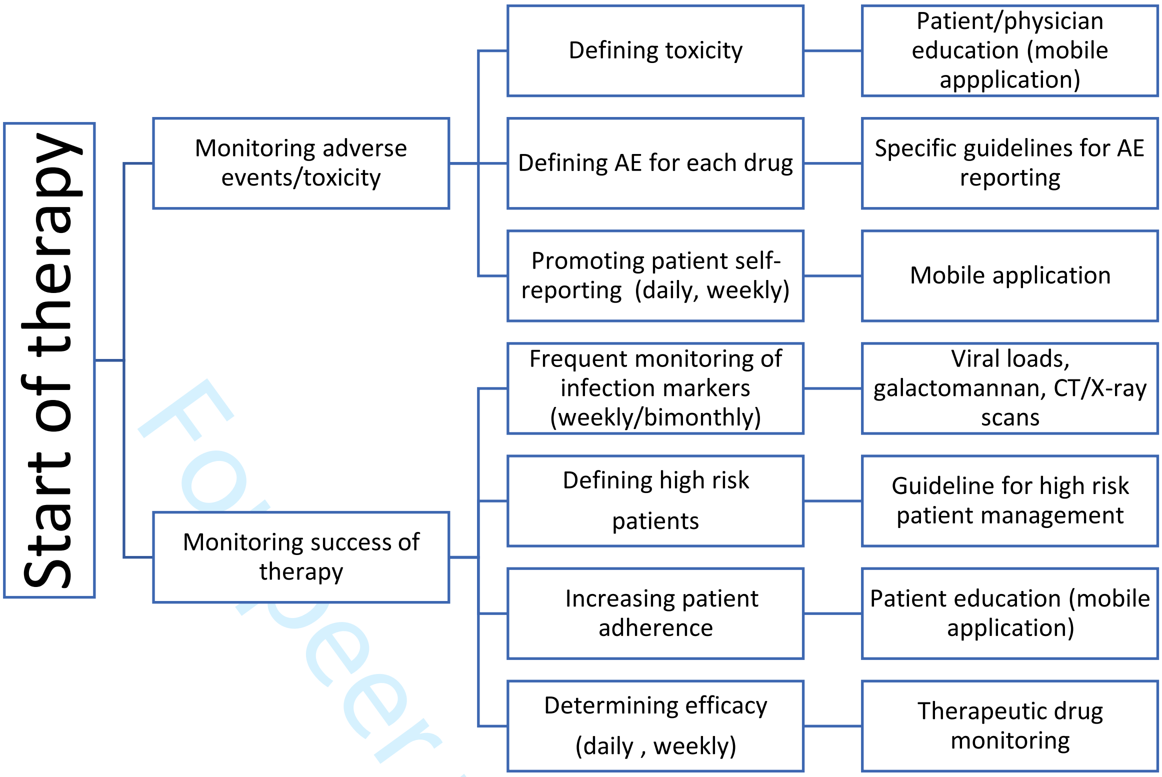

For peer review only

For peer review only

Appendix 1. Study protocol (PROSPERO CRD42017077606)

PROSPERO International prospective register of systematic reviews

Review title and timescale

1 Review title

Exploring failure of antimicrobial prophylaxis and pre-emptive therapy for transplant recipients: a systematic review

2 Original language title

Not applicable

3 Anticipated or actual start date

01 October 2017

4 Anticipated completion date

09 February 2019

Review team details

5 Named contact

Anne-Grete Märtson

6 Named contact email

a.martson@umcg.nl

7 Named contact address

Dept. of Clinical Pharmacy and Pharmacology; EB 70 UMCG Hanzeplein 1, PO BOX 30001, 9700 RB Groningen the Netherlands

8 Named contact phone number

+37253325121

9 Organisational affiliation of the review

University Medical Center Groningen

Website address: umcg.nl

10 Review team members and their organisational affiliations

	Anne-Grete	Märtson	UMCG, the Netherlands
Dr	Martijn	Bakker	UMCG, the Netherlands
Prof.Dr.	Tjip.S.	van der Werf	UMCG, the Netherlands

For peer review only

Dr	Jan-Willem	Alffenaar	UMCG, the Netherlands

11 Funding sources/sponsors

This systematic review will be part of a PhD thesis that is funded by University Medical Centre Groningen and the European Union's Horizon 2020 research and innovation programme Under the Marie Skłodowska-Curie grant agreement 713660.

12 Conflicts of interest

There are no actual or potential conflicts of interest.

13 Collaborators

Title	First name	Last name	Organisation details
Dr	Hans	Blokzijl	UMCG, the Netherlands
Dr	Erik A.M.	Verschuuren	UMCG, the Netherlands
Dr	L.F.R.	Span	UMCG, the Netherlands
Dr	Stefan P.	Berger	UMCG, the Netherlands

Review methods

14 Review question(s)

It is widely known that solid organ and allogenic stem cell transplant patients are especially susceptible to opportunistic infections like Cytomegalovirus (CMV), *Pneumocystis jirovecii* pneumonia (PCP) and invasive fungal infections. In recent years, organ transplantation has developed greatly. Transplantation has become available for a bigger patient population and this has led to a wider range of infections after transplantation. The concern with appropriate prophylactic regimens remain as antifungal prophylaxis varies exceptionally among different transplant centres.

To prevent these infections from occurring different prophylactic strategies are used before and after transplantation like ganciclovir and valganciclovir for CMV and trimethoprim-sulfamethoxazole (TMP+SMX) for PCP. This has proven to be a beneficial approach, despite that there is still a proportion of patients whose prophylactic treatment fails as they develop breakthrough infections, suffer from adverse events or

For peer review only - http://bmjopen.bmj.com/site/about/guidelines.xhtml

simply do not tolerate medications.

This systematic review is aiming to summarise the main reasons why prophylaxis is failed for non-HIV immunocompromised patients looking at prospective studies done on this topic.

The objectives of this study are to review and analyse literature to determine which factors might contribute to failure of prophylaxis of opportunistic infections in transplant recipients.

The potential factors for failure are drug-drug interactions, microbiological susceptibility, drug pharmacokinetics, the dose of the medication, route of administration, transplant (specific organ or allogenic stem cell), status of the immunocompromised patient and medication compliance.

15 Searches

This systematic review will include prospective randomized controlled trials and prospective single-arm studies. There are no restrictions for the setting or language. Studies published from 1.01.2010 to present will be included to the systematic review.

To identify studies for this systematic review these databases will be searched: MEDLINE, EMBASE, Cochrane Central Register of Controlled Trials (CENTRAL) and PubMed.

16 URL to search strategy

PubMed search strategy

("Immunity, Cellular"[Mesh] OR "Immunocompromised Host"[Mesh] OR "Transplants"[Mesh] OR "Transplantation"[Mesh] OR immunocompromised [tiab] OR transplant*[tiab])

AND

("valganciclovir" [Supplementary Concept] OR "Ganciclovir"[Mesh] OR "Trimethoprim, Sulfamethoxazole Drug Combination"[Mesh] OR "Ciprofloxacin"[Mesh:NoExp] OR "posaconazole" [Supplementary Concept] OR "Voriconazole"[Mesh] OR "Fluconazole"[Mesh] OR valganciclovir [tiab] OR Ganciclovir [tiab] OR Trimethoprim [tiab] OR Sulfamethoxazole [tiab] OR Ciprofloxacin [tiab] OR posaconazole [tiab] OR Voriconazole [tiab] OR Fluconazole [tiab] OR cotrimoxazole [tiab)

NOT (HIV OR Human immunodeficiency virus)

AND

("Randomized Controlled Trial" [Publication Type] OR randomi*[tiab] OR randomly[tiab] OR trial[ti] OR prospective [tiab] OR single arm [tiab])

For peer review only - http://bmjopen.bmj.com/site/about/guidelines.xhtml

17 Condition or domain being studied

Prophylaxis for opportunistic infections in transplant recipients.

18 Participants/population

The study will include adult patients (16 years and older) who have received:

- allogeneic stem cell transplant
- lung transplant
- kidney transplant
- liver transplant
- heart transplant
- pancreas transplant
- small bowel transplant

19 Intervention(s), exposure(s)

All patients who have received prophylaxis or preemptive therapy for:

- PCP with TMP+SMX
- febrile neutropenia with ciprofloxacin
- cytomegalovirus and human herpesvirus type 6 with ganciclovir and/or valganciclovir
- invasive fungal infections with posaconazole and/or voriconazole and/or fluconazole and/or itraconazole

20 Comparator(s)/control

Placebo or active comparator.

21 Types of study to be included

This systematic review will include prospective randomized controlled trials and prospective single-arm studies.

22 Context

There are no restrictions for the setting.

23 Primary outcome(s)

Primary outcomes:

- adverse effects leading to stopping of treatment or switching medication or dose reduction

For peer review only

- breakthrough infection

24 **Secondary outcomes**

Secondary outcomes:

- resistance to antimicrobials/antifungals/antivirals
- death

25 **Data extraction (selection and coding)**

The literature search and data extraction for inclusion and eligibility for this systematic review will be done according to the inclusion criteria by two authors independently. If there are discrepancies between the results these will be discussed and resolved with a third author.

If there is data missing or additional questions from the selected studies then the authors of these studies will be contacted directly.

A form will be developed to document the characteristics and quality of the included studies.

26 **Risk of bias (quality) assessment**

Risk of bias in individual studies will be assessed independently by the two researchers who did the study selection and data extraction. For assessing bias in individual studies Revised Cochrane risk-of-bias tool for randomized trials (RoB 2) will be used for RCTs and Risk of Bias in Non-randomized Studies of Interventions (ROBINS-I) will be used for prospective single-arm studies

If there are discrepancies between the authors these will be discussed and resolved with a third author.

27 **Strategy for data synthesis**

The criteria how the data will be quantitatively synthesised and if and how subgroup analysis will be done can be determined after the data is available.

The possibility of performing a meta-analysis and summary measures can also be determined after literature search and when we have done the data extraction.
After the preliminary searches we expect that there will not be sufficient data to conduct a meta-analysis.

28 **Analysis of subgroups or subsets**

The possibility and method for subgroup analysis can be determined after the data is available and

For peer review only - http://bmjopen.bmj.com/site/about/guidelines.xhtml

extracted.

If subgroup analysis is applicable then the subgroups could be different transplant types.

Review general information

29 Type and method of review

Systematic review

30 Language

English

31 Country

The Netherlands

32 Other registration details

Not applicable

33 Reference and/or URL for published protocol

Not applicable

34 Dissemination plans

This systematic review will be submitted to a journal specific to this field.

35 Keywords

systematic review; immunocompromised patients; prophylaxis; antimicrobials; antifungals; antivirals; PCP; CMV; febrile neutropenia; invasive fungal infection; opportunistic infection; transplantation

36 Details of any existing review of the same topic by the same authors

There has not been a previous systematic review done on this topic before.

37 Current review status

Conpleted (not published)

38 Any additional information

This review is part of planning further retrospective or prospective studies on prophylactic regimens for immunocompromised patients.

39 Details of final report/publication(s)

Not applicable

726

For peer review only

Appendix 2. PRISMA 2009 Checklist

Section/topic	#	Checklist item	Reported on page #
**TITLE**			
Title	1	Identify the report as a systematic review, meta-analysis, or both.	1
**ABSTRACT**			
Structured summary	2	Provide a structured summary including, as applicable: background; objectives; data sources; study eligibility criteria, participants, and interventions; study appraisal and synthesis methods; results; limitations; conclusions and implications of key findings; systematic review registration number.	2
**INTRODUCTION**			
Rationale	3	Describe the rationale for the review in the context of what is already known.	3
Objectives	4	Provide an explicit statement of questions being addressed with reference to participants, interventions, comparisons, outcomes, and study design (PICOS).	3
**METHODS**			
Protocol and registration	5	Indicate if a review protocol exists, if and where it can be accessed (e.g., Web address), and, if available, provide registration information including registration number.	3
Eligibility criteria	6	Specify study characteristics (e.g., PICOS, length of follow-up) and report characteristics (e.g., years considered, language, publication status) used as criteria for eligibility, giving rationale.	4
Information sources	7	Describe all information sources (e.g., databases with dates of coverage, contact with study authors to identify additional studies) in the search and date last searched.	4
Search	8	Present full electronic search strategy for at least one database, including any limits used, such that it could be repeated.	4, 46-47
Study selection	9	State the process for selecting studies (i.e., screening, eligibility, included in systematic review, and, if applicable, included in the meta-analysis).	4
Data collection process	10	Describe method of data extraction from reports (e.g., piloted forms, independently, in duplicate) and any processes for obtaining and confirming data from investigators.	4-5
Data items	11	List and define all variables for which data were sought (e.g., PICOS, funding sources) and any assumptions and simplifications made.	4

For peer review only

Risk of bias in individual studies	12	Describe methods used for assessing risk of bias of individual studies (including specification of whether this was done at the study or outcome level), and how this information is to be used in any data synthesis.	5
Summary measures	13	State the principal summary measures (e.g., risk ratio, difference in means).	5
Synthesis of results	14	Describe the methods of handling data and combining results of studies, if done, including measures of consistency (e.g., I^2) for each meta-analysis.	NA

Section/topic	#	Checklist item	Reported on page #
Risk of bias across studies	15	Specify any assessment of risk of bias that may affect the cumulative evidence (e.g., publication bias, selective reporting within studies).	5
Additional analyses	16	Describe methods of additional analyses (e.g., sensitivity or subgroup analyses, meta-regression), if done, indicating which were pre-specified.	5
**RESULTS**			
Study selection	17	Give numbers of studies screened, assessed for eligibility, and included in the review, with reasons for exclusions at each stage, ideally with a flow diagram.	23
Study characteristics	18	For each study, present characteristics for which data were extracted (e.g., study size, PICOS, follow-up period) and provide the citations.	27-31
Risk of bias within studies	19	Present data on risk of bias of each study and, if available, any outcome level assessment (see item 12).	7, 35-36
Results of individual studies	20	For all outcomes considered (benefits or harms), present, for each study: (a) simple summary data for each intervention group (b) effect estimates and confidence intervals, ideally with a forest plot.	33-36
Synthesis of results	21	Present results of each meta-analysis done, including confidence intervals and measures of consistency.	NA
Risk of bias across studies	22	Present results of any assessment of risk of bias across studies (see Item 15).	35-36
Additional analysis	23	Give results of additional analyses, if done (e.g., sensitivity or subgroup analyses, meta-regression [see Item 16]).	5-7
**DISCUSSION**			
Summary of evidence	24	Summarize the main findings including the strength of evidence for each main outcome; consider their relevance to key groups (e.g., healthcare providers, users, and policy makers).	7-10
Limitations	25	Discuss limitations at study and outcome level (e.g., risk of bias), and at review-level (e.g., incomplete retrieval of identified research, reporting bias).	9-10
Conclusions	26	Provide a general interpretation of the results in the context of other evidence, and implications for future research.	10

For peer review only

FUNDING			
Funding	27	Describe sources of funding for the systematic review and other support (e.g., supply of data); role of funders for the systematic review.	10

From: Moher D, Liberati A, Tetzlaff J, Altman DG, The PRISMA Group (2009). Preferred Reporting Items for Systematic Reviews and Meta-Analyses: The PRISMA Statement. PLoS Med 6(7): e1000097. doi:10.1371/journal.pmed1000097

For more information, visit: **www.prisma-statement.org**.

For peer review only

For peer review only - http://bmjopen.bmj.com/site/about/guidelines.xhtml

Appendix 3. Search strategy of EMBASE and CENTRAL

MEDLINE (PubMed) search strategy

("Immunity, Cellular"[Mesh] OR "Immunocompromised Host"[Mesh] OR "Transplants"[Mesh] OR "Transplantation"[Mesh] OR "Transplant Recipients"[Mesh] OR immunocompromised [tiab] OR kidney transplant* [tiab] OR heart transplant* [tiab] OR pancreas transplant* [tiab] OR lung transplant* [tiab] OR stem cell transplant* [tiab] OR small bowel transplant* [tiab] OR liver transplant* [tiab] OR small-bowel transplant* [tiab] OR allogeneic stem cell transplant* [tiab] OR transplant*[tiab])

AND

("valganciclovir" [Supplementary Concept] OR "Antibiotic Prophylaxis"[Mesh] OR "Ganciclovir"[Mesh] OR "Trimethoprim, Sulfamethoxazole Drug Combination"[Mesh] OR "Ciprofloxacin"[Mesh:NoExp] OR "posaconazole" [Supplementary Concept] OR "Voriconazole"[Mesh] OR "Fluconazole"[Mesh] OR "Anti-Infective Agents"[Mesh] OR valganciclovir [tiab] OR Ganciclovir [tiab] OR Trimethoprim [tiab] OR Sulfamethoxazole [tiab] OR Ciprofloxacin [tiab] OR posaconazole [tiab] OR Voriconazole [tiab] OR Fluconazole [tiab] OR bactrim [tiab] OR biseptol [tiab] OR ciprinol [tiab] OR antimicrobial [tiab] OR antibacterial [tiab] OR anti-microbial [tiab] anti-bacterial [tiab] OR anti-fungal [tiab] OR antifungal [tiab] OR antiviral [tiab] OR anti-viral [tiab] OR itraconazole [tiab] OR cotrimoxazole [tiab) NOT (HIV OR Human immunodeficiency virus)

AND

("Randomized Controlled Trial" [Publication Type] OR "Randomized Controlled Trials as Topic"[Mesh] OR randomi*[tiab] OR randomly[tiab] OR trial[tiab] OR prospective [tiab] OR single arm [tiab).

EMBASE search strategy

('immunocompromised patient'/exp OR 'transplantation'/exp OR transplant*:ab,ti OR 'graft recipient'/exp OR immunocompromised:ab,ti OR ‘kidney transplant*’:ab,ti OR ‘heart

For peer review only

transplant*’:ab,ti OR ‘pancreas transplant*’:ab,ti OR ‘lung transplant*’:ab,ti OR ‘small bowel transplant*’:ab,ti OR ‘liver transplant*’:ab,ti OR ‘small-bowel transplant*’:ab,ti OR ‘allogeneic stem cell transplant*’:ab,ti OR ‘stem cell transplant*’:ab,ti)

AND

('valganciclovir'/exp OR 'ganciclovir'/exp OR 'cotrimoxazole'/exp OR 'ciprofloxacin'/exp OR 'posaconazole'/exp OR 'voriconazole'/exp OR 'fluconazole'/exp OR 'itraconazole'/exp OR valganciclovir:ab,ti OR ganciclovir:ab,ti OR trimethoprim:ab,ti OR sulfamethoxazole:ab,ti OR ciprofloxacin:ab,ti OR posaconazole:ab,ti OR voriconazole:ab,ti OR itraconazole:ab,ti OR fluconazole:ab,ti OR bactrim:ab,ti OR biseptol:ab,ti OR ciprinol:ab,ti) NOT (HIV OR Human immunodeficiency virus) AND

('randomized controlled trial'/exp OR 'prospective study'/exp OR randomi*:ab,ti OR randomly:ab,ti OR trial:ab,ti OR prospective:ab,ti OR ‘single arm’:ab,ti)

CENTRAL search strategy

(Immunocompromised Host OR Transplants OR Transplantation OR Transplant Recipients OR immunocompromised OR transplant* OR kidney transplant* OR heart transplant* OR pancreas transplant* OR lung transplant* OR stem cell transplant* OR small bowel transplant* OR liver transplant* OR small-bowel transplant* OR allogeneic stem cell transplant*)

AND

(valganciclovir OR Antibiotic Prophylaxis OR Ganciclovir OR Trimethoprim, Sulfamethoxazole Drug Combination OR Ciprofloxacin OR posaconazole OR Voriconazole OR Fluconazole OR Anti-Infective Agents OR Trimethoprim OR Sulfamethoxazole OR bactrim OR biseptol OR ciprinol OR itraconazole OR antimicrobial OR antibacterial OR anti-microbial or anti-bacterial OR anti-fungal OR antifungal OR antiviral OR anti-viral OR cotrimoxazole) NOT (HIV OR Human immunodeficiency virus)

AND (Randomized Controlled Trial OR randomi* OR randomly OR trial OR prospective OR single arm)

For peer review only

Appendix 4. Data extraction form

- Reference (First author / Year / Title / Journal / Country)

- Study design
    - RCT
    - Single-arm study

- Sample size
    - Number of subjects started study
    - Number of subjects finished study

- Duration of study
    - Prophylaxis/preemptive therapy duration
    - Follow-up duration

- Study population
    - Age range
    - Transplanted organ
    - Underlying diseases

- Intervention
    - Medication used for prohylaxis
    - Comparison if applicable

- Main outcome measures
    - Primary outcomes
    - Secondary outcomes

- Failure of prophylaxis

- Risk of bias
    - RoB or ROBINS risk of bias score – low/high/some concerns

- Strengths and limitations of study

- Key conclusions of authors

For peer review only

Appendix 5. Background of detection of failure in the included studies

Table 1. RCT study reporting

							Reference	RCT								
	Humar 2010[1,2]	Halim 2016, Gheith 2017	Witzke 2012, 2018	Reischig 2012	Reichig 2011, 2015	Togashi 2011	Padulles 2016	Palmer 2010 Finlen Copeland 2011	Chawla 2011	Boeckh 2015	Kim 2010	Parrella 2012	Wintston 2014	Chaftari 2012	Huang 2012	Park 2016
**Description of high risk patients**	*	*	*	*	*		*	*	*	*	*	*	*	*		*
**Patient adherence reported**	*	*	*	*	*	*	*	*		*					*	*
**AE/toxicity described/ reported**	*	*	*	*	*	*	*	*	*	*	*		*	*#	*	
**Definition of breakthrough infection**	*	*	*	*	*	*	*	*	PT	PT	PT	*	*	*	*	*
**Breakthrough infection reported**	*	*	*	*	*	*	*	*	PT	PT	PT		*	*	*	*
**Insufficient reporting of topics above mentioned in limitations**						*										

PT – pre-emptive therapy, not applicable

# - toxicity explicitly defined, measurement described

For peer review only - http://bmjopen.bmj.com/site/about/guidelines.xhtml

Table 2. Single-arm study reporting

Topic				Reference	Single-arm			
	Montejo 2010	Nanmoku 2018	Perrottet 2009 Manuel 2010	McGillicuddy 2010	Takenaka 2012	Park 2012	Mitsani 2012	Cordonnier 2010
Description of high risk patients	*	*	*	*	*	*		
Patient adherence reported								*
AE/toxicity described/ reported	*		*#	*	*	*		*
Definition of breakthrough infection	*	*	*	PT	PT	PT	*	*
Breakthrough infection reported	*			PT	PT	PT	*	*
Insufficient reporting of failure mentioned in limitations								

PT – pre-emptive therapy, not applicable

# - toxicity explicitly defined, measurement described

PRISMA 2009 Checklist

Section/topic	#	Checklist item	Reported on page #
**TITLE**			
Title	1	Identify the report as a systematic review, meta-analysis, or both.	1
**ABSTRACT**			
Structured summary	2	Provide a structured summary including, as applicable: background; objectives; data sources; study eligibility criteria, participants, and interventions; study appraisal and synthesis methods; results; limitations; conclusions and implications of key findings; systematic review registration number.	2
**INTRODUCTION**			
Rationale	3	Describe the rationale for the review in the context of what is already known.	3
Objectives	4	Provide an explicit statement of questions being addressed with reference to participants, interventions, comparisons, outcomes, and study design (PICOS).	3
**METHODS**			
Protocol and registration	5	Indicate if a review protocol exists, if and where it can be accessed (e.g., Web address), and, if available, provide registration information including registration number.	3
Eligibility criteria	6	Specify study characteristics (e.g., PICOS, length of follow-up) and report characteristics (e.g., years considered, language, publication status) used as criteria for eligibility, giving rationale.	4
Information sources	7	Describe all information sources (e.g., databases with dates of coverage, contact with study authors to identify additional studies) in the search and date last searched.	4
Search	8	Present full electronic search strategy for at least one database, including any limits used, such that it could be repeated.	4, 45-46
Study selection	9	State the process for selecting studies (i.e., screening, eligibility, included in systematic review, and, if applicable, included in the meta-analysis).	4
Data collection process	10	Describe method of data extraction from reports (e.g., piloted forms, independently, in duplicate) and any processes for obtaining and confirming data from investigators.	4-5
Data items	11	List and define all variables for which data were sought (e.g., PICOS, funding sources) and any assumptions and simplifications made.	4
Risk of bias in individual studies	12	Describe methods used for assessing risk of bias of individual studies (including specification of whether this was done at the study or outcome level), and how this information is to be used in any data synthesis.	5
Summary measures	13	State the principal summary measures (e.g., risk ratio, difference in means).	5
Synthesis of results	14	Describe the methods of handling data and combining results of studies, if done, including measures of consistency (e.g., I^2) for each meta-analysis.	NA

For peer review only - http://bmjopen.bmj.com/site/about/guidelines.xhtml

PRISMA 2009 Checklist

Section/topic	#	Checklist item	Reported on page #
Risk of bias across studies	15	Specify any assessment of risk of bias that may affect the cumulative evidence (e.g., publication bias, selective reporting within studies).	5
Additional analyses	16	Describe methods of additional analyses (e.g., sensitivity or subgroup analyses, meta-regression), if done, indicating which were pre-specified.	5
**RESULTS**			
Study selection	17	Give numbers of studies screened, assessed for eligibility, and included in the review, with reasons for exclusions at each stage, ideally with a flow diagram.	23
Study characteristics	18	For each study, present characteristics for which data were extracted (e.g., study size, PICOS, follow-up period) and provide the citations.	27-31
Risk of bias within studies	19	Present data on risk of bias of each study and, if available, any outcome level assessment (see item 12).	7, 35-36
Results of individual studies	20	For all outcomes considered (benefits or harms), present, for each study: (a) simple summary data for each intervention group (b) effect estimates and confidence intervals, ideally with a forest plot.	33-36
Synthesis of results	21	Present results of each meta-analysis done, including confidence intervals and measures of consistency.	NA
Risk of bias across studies	22	Present results of any assessment of risk of bias across studies (see Item 15).	35-36
Additional analysis	23	Give results of additional analyses, if done (e.g., sensitivity or subgroup analyses, meta-regression [see Item 16]).	5-7
**DISCUSSION**			
Summary of evidence	24	Summarize the main findings including the strength of evidence for each main outcome; consider their relevance to key groups (e.g., healthcare providers, users, and policy makers).	7-10
Limitations	25	Discuss limitations at study and outcome level (e.g., risk of bias), and at review-level (e.g., incomplete retrieval of identified research, reporting bias).	9-10
Conclusions	26	Provide a general interpretation of the results in the context of other evidence, and implications for future research.	10
**FUNDING**			
Funding	27	Describe sources of funding for the systematic review and other support (e.g., supply of data); role of funders for the systematic review.	10

From: Moher D, Liberati A, Tetzlaff J, Altman DG, The PRISMA Group (2009). Preferred Reporting Items for Systematic Reviews and Meta-Analyses: The PRISMA Statement. PLoS Med 6(7): e1000097. doi:10.1371/journal.pmed1000097

For more information, visit: **www.prisma-statement.org**.

For peer review only - http://bmjopen.bmj.com/site/about/guidelines.xhtml
